# Microbial communities form rich extracellular metabolomes that foster metabolic interactions and promote drug tolerance

**DOI:** 10.1038/s41564-022-01072-5

**Published:** 2022-03-21

**Authors:** Jason S. L. Yu, Clara Correia-Melo, Francisco Zorrilla, Lucia Herrera-Dominguez, Mary Y. Wu, Johannes Hartl, Kate Campbell, Sonja Blasche, Marco Kreidl, Anna-Sophia Egger, Christoph B. Messner, Vadim Demichev, Anja Freiwald, Michael Mülleder, Michael Howell, Judith Berman, Kiran R. Patil, Mohammad Tauqeer Alam, Markus Ralser

**Affiliations:** 1 The Molecular Biology of Metabolism Laboratory, The Francis Crick Institute, London, UK; 2 Department of Biochemistry, University of Cambridge, Cambridge, UK; 3 Medical Research Council Toxicology Unit, University of Cambridge, Cambridge, UK; 4 Structural and Computational Biology Unit, European Molecular Biology Laboratory, Heidelberg, Germany; 5 Department of Biochemistry, Charité University Medicine, Berlin, Germany; 6 High-Throughput Screening, The Francis Crick Institute, London, UK; 7 Core Facility – High Throughput Mass Spectrometry, Charité University Medicine, Berlin, Germany; 8 Shmunis School of Biomedical and Cancer Research, George S. Wise Faculty of Life Sciences, Tel Aviv University, Ramat Aviv, Israel; 9 Department of Biology, College of Science, United Arab Emirates University, Al-Ain, UAE; 10 Warwick Medical School, University of Warwick, Coventry, UK

**Keywords:** Microbial ecology, Antimicrobials

## Abstract

Microbial communities are composed of cells of varying metabolic capacity, and regularly include auxotrophs that lack essential metabolic pathways. Through analysis of auxotrophs for amino acid biosynthesis pathways in microbiome data derived from >12,000 natural microbial communities obtained as part of the Earth Microbiome Project (EMP), and study of auxotrophic–prototrophic interactions in self-establishing metabolically cooperating yeast communities (SeMeCos), we reveal a metabolically imprinted mechanism that links the presence of auxotrophs to an increase in metabolic interactions and gains in antimicrobial drug tolerance. As a consequence of the metabolic adaptations necessary to uptake specific metabolites, auxotrophs obtain altered metabolic flux distributions, export more metabolites and, in this way, enrich community environments in metabolites. Moreover, increased efflux activities reduce intracellular drug concentrations, allowing cells to grow in the presence of drug levels above minimal inhibitory concentrations. For example, we show that the antifungal action of azoles is greatly diminished in yeast cells that uptake metabolites from a metabolically enriched environment. Our results hence provide a mechanism that explains why cells are more robust to drug exposure when they interact metabolically.

## Abstract (web summary)

Using microbiome data analysis and a self-establishing metabolically cooperating yeast community model, the authors show that the presence of auxotrophs in a microbial community increases metabolic interactions between cells and fosters antimicrobial drug tolerance.

## Main

Metabolism occurs both within and between cells. The exchange of metabolites is increasingly recognized as a critical feature for the physiology of microbial cells that are growing as part of community structures, where uptake and secretion of metabolites are defining characteristics of metabolism that lead to cross-feeding and collective survival^1–6^. Because metabolic processes are coupled to each other as part of metabolic networks, microbial metabolic interdependencies fundamentally contribute to the physiology of cells that are part of communities^7,8^. Indeed, most microbial cells have broad-ranging biosynthetic capacities and can synthesize a wide range of biomolecules that they require for growth. In the presence of the respective metabolites in the extracellular environment, however—for instance, when they are released at sufficient concentration by cogrowing cells—they inhibit the respective biosynthetic pathways and uptake metabolites rather than synthesizing them^9^. Such metabolic flexibility results in cell–cell metabolic interactions and allows communities to effectively exploit resources, to save costs and to improve biomass formation^10,11^. Evidence for a high degree of metabolite exchange is provided by the regular presence of auxotrophic species within microbial communities. Auxotrophs lack the essential metabolic pathways required to synthesize amino acids, nucleotides, vitamins, fatty acids or metabolic coenzymes at the genetic level^1,12–16^. In contrast to prototrophs that can flexibly switch between metabolite synthesis and uptake, auxotrophs are constitutively dependent on the extracellular availability of these metabolites for growth^7,17^. Auxotrophs can hence persist in communities only where the essential metabolites are consistently available at growth-supporting concentrations.

A switch from self-synthesis to the uptake of a metabolite affects the physiological parameters of microbial cells and affects their stress tolerance^18–20^. For instance, *Saccharomyces cerevisiae* cells uptake much higher concentrations of lysine than they would require for growth. This lysine harvest allows them to configure their metabolism to maintain higher concentrations of glutathione, which increases oxidant tolerances^21^. Interestingly, several recent reports have linked the metabolic properties of both bacterial and fungal microbes to their ability to mount resistance (defined as robust growth in the presence of the antimicrobial^22^), tolerance (slower growth of subpopulations in the presence of drug concentrations above minimum inhibitory concentration (MIC) drug concentrations^22^) and resilience (used herein to describe situations that involve both tolerance and resistance mechanisms) against antimicrobial substances^23,24^. In parallel, there is an active discussion regarding whether and how the exchange of metabolites between cells is influencing the evolution of resistance genes. ‘Weakest links’ in metabolite exchange chains can slow the spread of drug resistance genes if their growth is impaired by antimicrobial exposure^25,26^.

We here describe a mechanism that links the presence of auxotrophs within microbial communities to an increase in metabolite exchange interactions in general, and show that communities gain robustness against antimicrobial substances as a consequence of metabolite exchange interactions. We observed that amino acid auxotrophs are found in the vast majority of microbiomes sequenced as part of the EMP^27^, and it triggered our particular attention that these auxotrophs are particularly frequent in host-associated communities. Searching for potential physiological consequences, we mined growth data for a panel of gut microbial species^28^ of which one-third were revealed to be auxotrophic for diverse amino acid biosynthetic pathways. We observed that amino acid auxotrophs achieve better growth in the presence of a large number of drugs. To shed light on the underlying mechanism, we made use of a tractable, isogenic system (SeMeCos) in budding yeast^29^. The SeMeCos model replicated the increased drug resilience of auxotrophs. Moreover, SeMeCos revealed system-wide metabolic flux changes that cause auxotrophs to enrich the communal metabolic environment. We describe how these metabolic changes are associated with overall increased efflux activity. These effects are not specific to metabolites but also reduce intracellular drug concentrations. Studying azoles as a potent class of antifungals, we report that lower intracellular drug levels allow metabolically interacting cells to grow above the minimal inhibitory drug concentrations.

## Results

### Amino acid auxotrophs are prevalent in natural communities

**Fig. 1:**
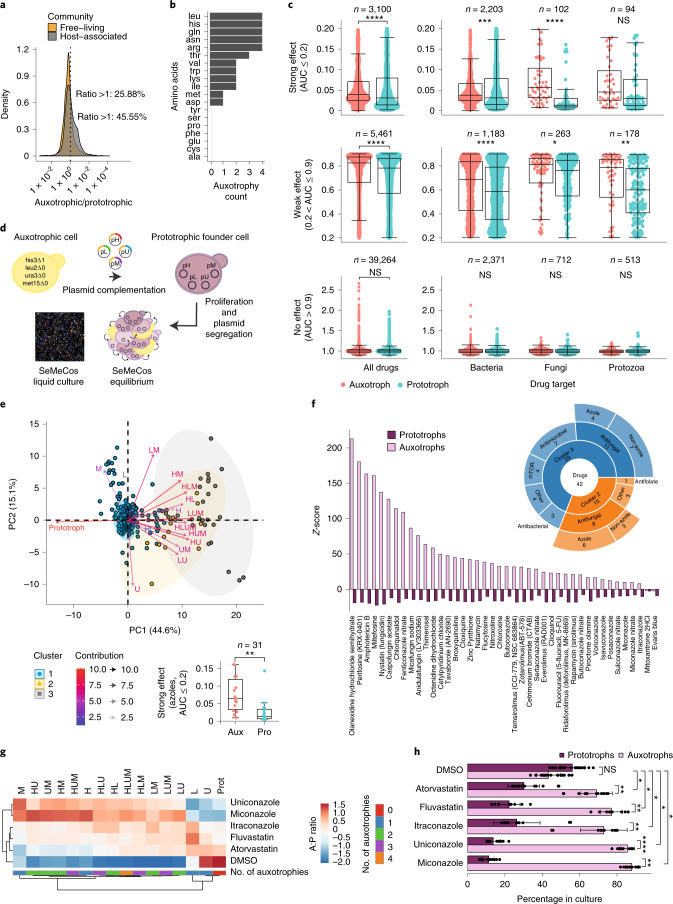
Auxotrophs are prevalent in host-associated microbial communities and are more drug resilient. **a**, Frequency of amino acid auxotrophic species in <12,000 microbial communities sequenced in the EMP^27,30^. Dotted line represents an auxotroph/prototroph (A:P) ratio of 1:1 in a given microbial community. **b**, Number of amino acid auxotrophies detected in 15/40 gut microbial species exposed to 1,197 bioactive drugs^28^. **c**, Growth, represented by AUC, between prototrophs and auxotrophs in drug-exposed microbiome species^28^. Microbe–drug pairs are binned according to strong (AUC > 0.2), weak (0.9 > AUC > 0.2) and no effect (AUC > 0.9) on growth across 40 drug-exposed microbial species. **d**, SeMeCos, a yeast-based, isogenic model for study of auxotrophic subpopulations^41^. **e**, Top, bottom left: SeMeCo colonies exposed to 900 FDA-approved drugs. PCA of *z*-scores assessing their impact on community composition. Hierarchical clustering identified two drug clusters (yellow and gray) affecting the A:P ratio. Arrows indicate variance driven by auxotrophic subpopulations. Bottom right: subset of gut microbiome AUC values for strong azoles identified by PCA. Significance was determined using a two-sided Wilcoxon rank-sum test, *P* = 5 × 10^–4^. **f**, A:P ratio within drug-treated SeMeCos based on highest *z*-scores. Classification of these drugs is based on known target/activity (sunburst plot). **g**, Composition analysis of SeMeCos treated with azoles/statins not present in **e**. Changes in A:P ratio are highlighted in red and blue.. Data are the median of *n* = 3 technical replicates within one independent experiment. Clustering based on subtraction of Pearson’s correlation from 1. **h**, Proportion of prototrophic and auxotrophic subpopulations following drug treatment. Data are median ± s.d. from 12 or 26 independent measurements for drug or DMSO, respectively, across two biologically independent experiments. Significance determined using two-sided Student’s *t*-test; *P < 0.05, **P < 0.005, ****P < 0.00005. Boxplots represent median (50% quantile (middle line)), lower (25%) and upper (75%) quantiles respectively). For **c** and **e**, significance was determined using Wilcoxon rank-sum test: *P < 0.05, **P < 0.005, ****P < 0.00005. Exact *P* values are available in Source Data 1. NS, not significant. Source data

We analyzed the frequency of auxotrophs in both free-living and host-associated natural communities using species composition data from EMP^27^. We determined the occurrence of auxotrophies using procedures described by Machado et al.^30^. Study of >12,000 communities present in the EMP dataset revealed that both free-living and host-associated communities contain a high frequency of species auxotrophic for amino acid biosynthetic pathways. Indeed, the data revealed that the presence of (amino acid) auxotrophs is an almost universal feature of microbial communities. Only six out of 12,538 communities in the dataset contained no amino acid auxotrophs, while one community contained not a single example. Moreover, many communities contained auxotrophs at high frequency, notably in host-associated communities, where we observed a particularly high abundance of auxotrophic species relative to prototrophs (45.55 versus 25.88% in free-living communities; Fig. 1a). We speculated that host-associated species are exposed to rich nutritional environments, which may explain why auxotrophs are more likely to prevail.

### Amino acid auxotrophs are less susceptible to drug effects

**Extended Data Fig. 1:**
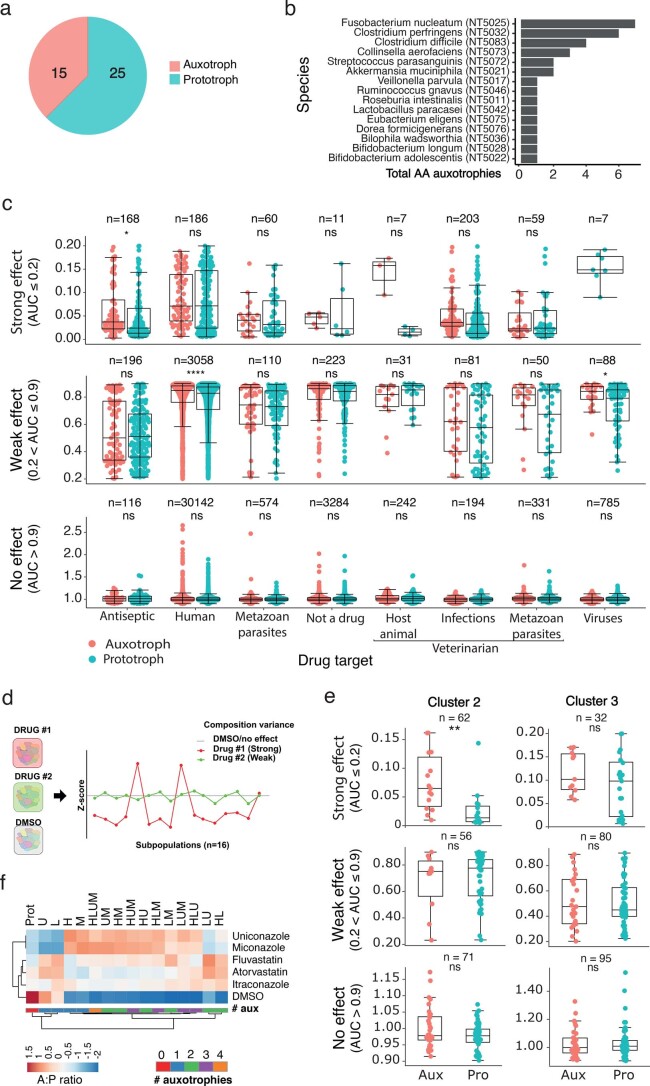
Auxotrophs are prevalent in drug-exposed natural and synthetic microbial communities and more drug resilient. (**a**) Frequency of amino acid auxotrophies across gut microbiome species, using the procedures in^1^. (**b**) Frequency of amino acid auxotrophies per gut microbiome auxotrophic species. (**c**) Growth, as measured by AUC, in drug exposed gut microbiome species across drug classes and metabolic background (auxotrophy vs prototrophy); n = number of drug-microbe pairs in each subset. (**d**) Screen setup, scoring and identification of drugs that modulate the auxotrophic composition in SeMeCos. Drug hits are identified by a high Z-score that indicates a significant shift in the SeMeCo composition compared to DMSO baseline. (**e**) Growth, as measured by AUC, in prototrophs and auxotrophs treated with drugs common to both the SeMeCo and gut microbiome drug screens, from Cluster 1 or 2 in the SeMeCo screen (e); n = number of drug-microbe pairs in each subset. (**f**) Flow cytometric analysis of the SeMeCo composition upon drug treatment, where red and blue indicate the relative increase or decrease, respectively, of a specific auxotrophic subpopulation (count of subpopulation/ total count). Median: n = 3 technical replicates within an independent experiment. Statistics by (c and f) two-sided Wilcoxon Rank Sum test, P-values are listed in the respective Source Data. Source data

Equally, host-associated communities are more frequently exposed to bioactive drugs, including those targeting human, fungal or bacterial cells, which can affect host microbiome composition. To test whether auxotrophy could have any impact in these drug responses, we made use of growth data from 40 gut microbiome members exposed to 1,197 bioactive drugs^28^. To determine the presence of auxotrophic species in these 40 gut microbiome members, we used the same predictor^30^ and found that 15 (37.5%) of the 40 species did bear auxotrophies (Extended Data Fig. 1a) in 12 amino acid biosynthetic pathways (Fig. 1b). Individual species possessed a maximum of seven amino acid auxotrophies in parallel, with the majority possessing between one and four in different combinations (Extended Data Fig. 1b). To illustrate the effect of drugs on growth in microbial species, we split these into three categories: (1) strong effect of the applied drug on growth (*n* = 3,100 drug–microbe pairs, one-sided Wilcoxon rank-sum test, false discovery rate (FDR) adjusted *P* = 5.4 × 10^–9^; (2) weak effect of drug on growth (*n* = 5,461 drug–microbe pairs, one-sided Wilcoxon rank-sum test, FDR adjusted *P* = 2.0 × 10^–15^); and (3) no effect of drug on growth (*n* = 39,264 drug–microbe pairs). We used the difference in mean area under the curve (AUC) values obtained from the growth curves, where AUC values were normalized to the value of 1 for the category “no growth effect” described in Maier et al.^28^ (Fig. 1c). Auxotrophs generally grew better in the presence of the selected drugs compared to prototrophs (expressed as higher AUC values). Enhanced growth of auxotrophs compared to prototrophs was detected in 8,561 drug–microbe combinations (Fig. 1c, top and middle). While other drugs had no effect on auxotrophs (Fig. 1c, bottom), we did not find a drug class that would disadvantage auxotrophs over prototrophs (Extended Data Fig. 1c). The effect of auxotrophy was most prevalent on the group of drugs having a suppressive effect on growth, but not in the group in which the drug had no general impact on growth, indicating that amino acid auxotrophy could buffer the impact of growth-inhibiting drug treatments (Fig. 1c). In particular, amino acid auxotrophs were more resilient to drugs directed against bacterial, fungal and protozoal targets (Wilcoxon rank-sum test; Fig. 1c).

### Metabolically cooperating auxotrophs are more drug resilient

The improved growth of auxotrophs versus prototrophs following drug exposure was observed for a broad spectrum of drug types and targets. This finding implies a general, target-independent mechanism that connects amino acid auxotrophy with microbial drug response. To shed light on this mechanism, we sought a tractable, isogenic model in which differences in drug tolerance are readily and directly attributable to auxotrophic mutations. SeMeCos represents a yeast model that allows the tracing of auxotrophic subpopulations and the dissection of auxotroph–prototroph interactions^19,29^. In SeMeCos, stochastic plasmid loss from a single prototrophic founder cell generates a community of auxotrophs and prototrophs in which auxotrophs require the exchange of amino acids (histidine, leucine, methionine) and a nucleobase (uracil) for survival and growth^19^ (Fig. 1d). Emerging auxotrophic subpopulations can be tracked because the SeMeCos colony grows exponentially, either by testing their auxotrophy through growth on appropriate media or by coupling the segregated metabolic marker to fluorescent proteins, and identifying auxotrophy through microscopy and fluorescent activated cell sorting (FACS). Ideal for our study, SeMeCos possesses a similar number of auxotrophies to gut microbial species, with similar pathways affected: 13 of the 15 auxotrophic gut species, as well as SeMeCos, had between one and four amino acid auxotrophies in different combinations (Extended Data Fig. 1b).

**Extended Data Fig. 2:**
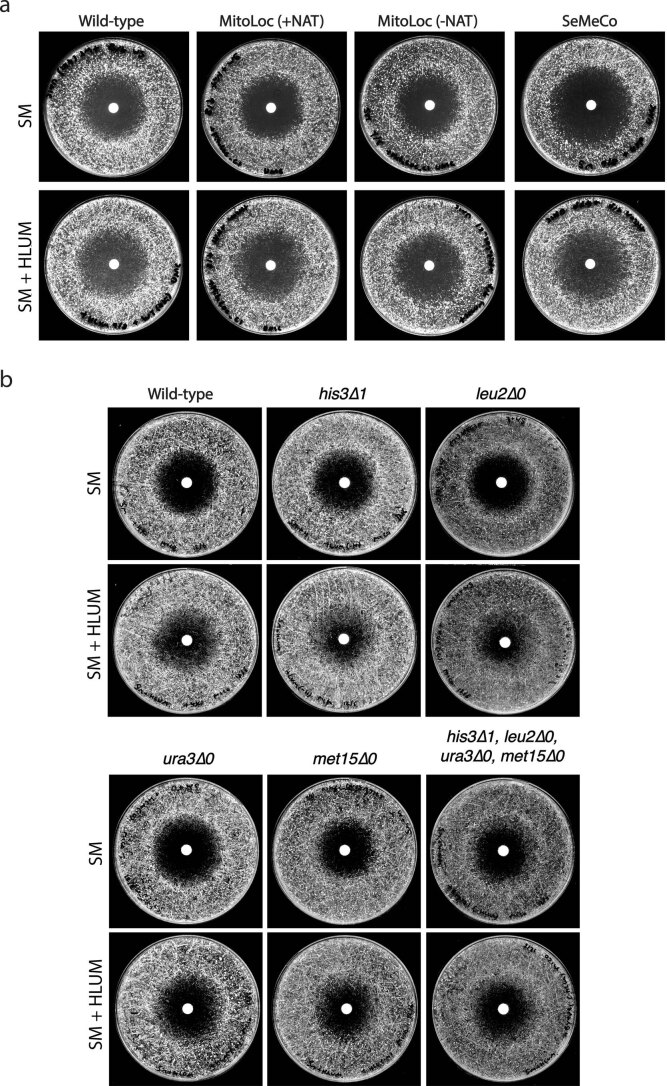
Increased proportion of auxotrophic subpopulations in SeMeCos are independent of drug effects on plasmid segregation or stability. (**a**) Drug disk diffusion assays of wild-type (WT) strains without plasmid or transformed with MitoLOC plasmid^2^ that encode for nourseothricin resistance (NAT) compared against the SeMeCo strain which carries 4 plasmids when exposed to uniconazole. (**b**) Disk diffusion assays of WT prototrophic strain compared against singly auxotrophic strains and quadruple auxotrophic parental strain when exposed to miconazole. SM indicated minimal media condition versus SM + HLUM whereby minimal media was supplemented with the 4 amino acids. Singly auxotrophic strains were supplemented with the respective amino acid to ensure their growth in the absence of genomic or plasmid complementation. Source data

To investigate whether auxotrophs within SeMeCos replicate increased robustness to drug exposure as observed in bacterial auxotrophs, we first generated a SeMeCos strain that coexpresses the prototrophic marker enzymes His3p, Leu2p, Met15p and Ura3p with fluorescent proteins that are codon optimized for expression in yeast^31^. We then established SeMeCos communities from the founder strain by serial spotting^29^, and exposed them to a compound library containing 900 diverse FDA-approved drugs at the typical concentration of 10 µM used in many pharmacological screens^32^. Of these drugs, 240 had also been tested in the gut microbiome species and, of these, 179 had a growth-inhibitory effect^28^. After cultivation of cells for 24 h in minimal synthetic medium, we used high-throughput fluorescence imaging to determine the auxotrophic composition of the cultures. For each auxotrophic subpopulation under each drug condition, a *z*-score was assigned reflecting the degree of deviation from the vehicle control (DMSO) population median (Extended Data Fig. 1d). Principal component analysis (PCA) of the raw scores and hierarchical clustering of the first two components revealed three clusters (Fig. 1e). Cluster 1 contained vehicle control (DMSO) and drug treatments with no effect on SeMeCos composition, as opposed to clusters 2 and 3, which primarily reflects an increase in auxotrophy independent of the number or type of auxotrophy (Fig. 1e, arrows). Most of the drugs contributing to clusters 2 and 3 were antimicrobial/antifungal compounds and, in a subset of cluster 2 drugs, the auxotrophs also demonstrated improved growth in the gut microbiome drug screen (Fig. 1e,f and Extended Data Fig. 1e). Notable was the robustness of auxotrophs against azole treatment (10/42 drugs in the SeMeCos drug screen hits), a class of compounds clinically used to treat fungal infections and which target the ergosterol biosynthetic pathway^33^ (Fig. 1e; Wilcoxon rank-sum test, *P* = 5.3 × 10^–4^). To test the generality of this finding in an independent experiment, we exposed SeMeCos to additional compounds belonging to the azole and statin classes, another group of compounds that affect the ergosterol biosynthetic pathway in yeast^34^. We then determined changes in the auxotrophic composition of SeMeCos by flow cytometry. SeMeCos exposed to these two drug classes showed a significant increase in the number of auxotrophic subpopulations when compared to vehicle control (DMSO) across two independent experiments (Fig. 1g,h and Extended Data Fig. 1f). To exclude the possibility that changes in drug response were due to altered segregation or stability of the plasmids that would also affect the proportion of auxotrophic subpopulations within SeMeCos, we transformed wild-type (WT) cells with an alternative centromeric plasmid (MitoLoc^35^), which allowed for selection not by auxotrophy but with the antibiotic nourseothricin. Moreover, we also tested drug tolerance in yeast strains in which the four marker genes were genomically integrated. In comparison of WT (no plasmid) and SeMeCos (four plasmids), as well as the genomically integrated strains, we observed no significant difference in growth response to uniconazole or miconazole, suggesting that the observed effects are not explained by the drug influencing either plasmid segregation or stability (Extended Data Fig. 2). In summary, together these results show that auxotrophy increases resilience to a broad range of bioactive compounds, particularly to azole antifungals and statins, not only in bacteria but also in isogenic yeast strains.

### A rich metabolic environment promotes drug resilience

**Fig. 2:**
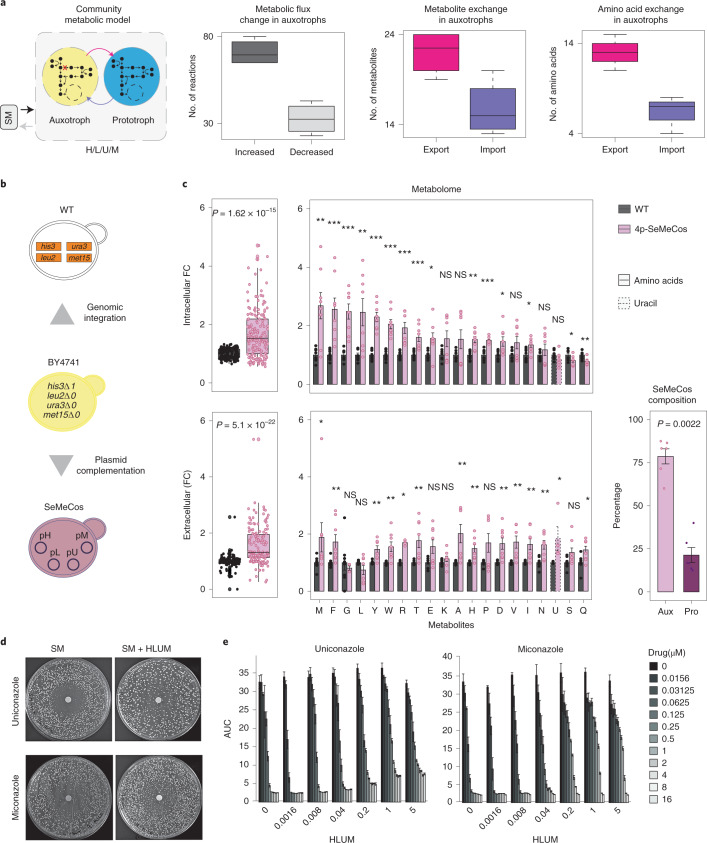
Auxotrophs promote a rich metabolic environment that increases drug tolerance in prototrophs. **a**, Left: genome-scale metabolic modeling in SeMeCos composed of auxotrophic and prototrophic subpopulations (*n* = 4, H/L/U/M community models). Significant increase (change >10%) in the number of metabolic fluxes (second from left, *P* = 7 × 10^–4^, metabolite exchange (second from right, *P* = 0.02) and exchange of amino acids (right, *P* = 0.002) in auxotrophs compared to prototrophs, shown as boxplots. Significance was calculated by two-sided Student’s *t*-test. **b**, Prototrophic community generated by genomic repair of *HIS3*, *LEU2*, *URA3* and *MET15* (WT*)*, as opposed to SeMeCos containing auxotrophs due to the stochastic segregation of plasmids containing the four auxotrophic markers. **c**, Left, middle: quantification of intra- and extracellular metabolites by mass spectroscopy^39^ in exponentially growing SeMeCos compared to WT cultures in SM medium. Metabolite concentrations were normalized to biomass, as assessed by optical density at OD_600_. Grouped metabolite comparison (box plots) significance was determined using a one-sided Kruskal–Wallis rank-sum test. Mean ± s.e.m. of *n* = 8 independent cultures per strain from two independent experiments. Individual metabolite comparison (bar plots) significance was determined using an unpaired two-sided Wilcoxon rank-sum test: *P < 0.05, **P < 0.005, ***P < 0.0005, ****P < 0.00005; exact *P* values are given in Source data 2. Right: proportion of auxotrophs and prototrophs in SeMeCos analyzed above, calculated by spotting colonies onto selective medium. Mean ± s.e.m. of *n* = 6 independent cultures from two independent experiments. **d**, Drug resistance (diameter of inhibition halo) and tolerance (growth within halo) measured by DDA^22^ in WT colonies in minimal (SM) or SM + HLUM-supplemented medium treated with uniconazole or miconazole, respectively. DDAs generated from WT cultures plated onto SM or SM + HLUM and/or azoles. One DDA per drug is illustrated. **e**, Growth of WT yeast cultures, assessed by OD_600_ and plotted as AUC, under increasing concentrations of uniconazole/miconazole and following increasing HLUM supplementation. Boxplots represent median (50% quantile (middle line)), lower (25%) and upper (75%) quantiles, respectively, of change in metabolic flux and amino acid and metabolite exchanges in auxotrophs compared to prototrophs in **a**, and pooled metabolite FC levels compared to WT in **c**. Source data

**Extended Data Fig. 3:**
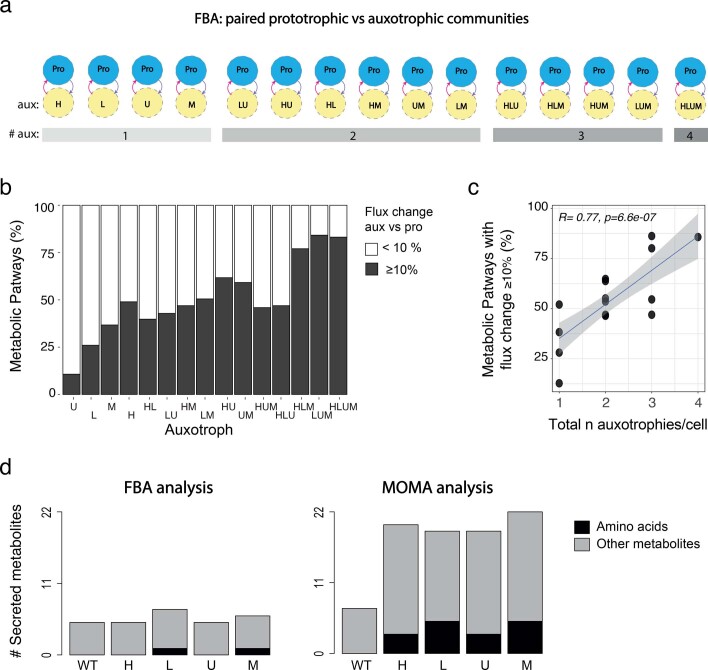
Increased metabolic flux change with increasing number of auxotrophies per cell. (**a**) Pair-wise FBA analysis assessing flux changes in communities composed of prototrophs and each of 15 auxotrophic subpopulations present in SeMeCos, relying on the exchanging of H, L, U and/or M. (**b**) Relative frequency of metabolic pathways with altered flux (flux change >10%) in the 15 different auxotrophs when interacting with a prototroph. (**c**) Pearson correlation between the proportion of metabolic pathways with altered flux (flux change >10%) and the total number of auxotrophies per cell. Error bands indicate the 95% confidence level interval for the predictions from the linear model. (**d**) Number of secreted metabolites in prototrophic (wild-type) and auxotrophic models in minimal media supplemented with required metabolites using FBA and MOMA simulation approaches^3^. The MOMA predicts an increase in metabolite excretion in single-metabolite supplemented auxotrophic yeast strain, compared to an FBA analysis. Source data

We interrogated a yeast genome-scale metabolic model using flux balance analysis (FBA)^36,37^ to map the community’s metabolic changes introduced by auxotrophy. To account for the exchange of amino acids and uracil between cells, we expanded the conventional FBA approach by including export and import reactions from a shared exometabolome, so that the model reflects metabolic interactions between the different metabotypes (metabolic backgrounds), specifically between cogrowing auxotrophs and prototrophs (Fig. 2a, left). The main objective function of the community model is the cumulative biomass of both auxotrophs and prototrophs. The analysis revealed that the change from self-synthesis to uptake of histidine (H), leucine (L), methionine (M) and/or uracil (U) not only affects the four perturbed biosynthetic pathways, but also a broad range of other metabolic fluxes coupled to them. Interestingly, comparison of the network reconstructions of auxotrophs with those of prototrophs interacting in a common metabolic environment revealed that auxotrophs had, in aggregate, more reactions with increased flux (flux change >10%). Consistently, a broader range of fluxes was reduced in prototrophs (two-sided Student’s *t*-test, *P* = 7 × 10^–4^) (Fig. 2a, second from left). Moreover, the spectrum of metabolites released from cells was increased in auxotrophs compared to prototrophs (Fig. 2a, second from right and far right). We then applied the expanded FBA approach, assessing flux changes between cells, to SeMeCos exchanging all four metabolites (H, L, U, M) and performed pairwise analysis between prototrophs and each of the 15 auxotrophic combinations (Extended Data Fig. 3a). We found that the number of auxotrophies positively correlated with the percentage of metabolic pathways with altered flux (flux change >10%; Extended Data Fig. 3b,c). In parallel, we simulated individual auxotrophic strains in minimal medium with the required metabolite supplementation using FBA and minimization of metabolic adjustment (MOMA) approaches^38^. FBA predicted a faster growth rate of auxotrophs while MOMA, similar to community-extended FBA, predicted an increase in metabolite excretion (Extended Data Fig. 3d).

**Extended Data Fig. 4:**
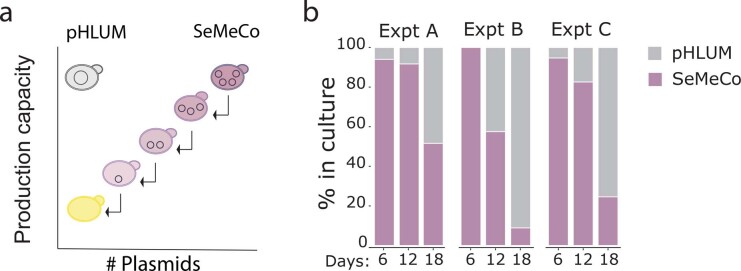
Prototrophs present growth advantage relative to co-growing auxotrophs. (**a**) pHLUM strain carrying a single plasmid complementing the 4 auxotrophies. Loss of the plasmid results in immediate reduction in production capacity for HLUM opposed to sequential loss in SeMeCos. (**b**) Competitive growth assay in which the pHLUM strain was co-cultured with the SeMeCo strain. Over 18 days, replated every 48 h, in 3 independent cultures (A, B and C), the pHLUM strain retaining full prototrophy, slowly outcompetes all SeMeCo derived subpopulations in terms of growth. Source data

To experimentally test these predictions, we tested for changes in both growth rate and exometabolome. To address the former, we engineered a SeMeCos founder strain carrying all four marker genes on a single plasmid (pHLUM). Because this strain cannot differentially segregate markers, its progeny either maintains prototrophy or becomes auxotrophic for all four metabolites simultaneously (Extended Data Fig. 4a). We then conducted a competitive growth experiment on minimal medium, by monitoring SeMeCos composition over time. The prototrophic (pHLUM) strain slowly but consistently became the dominant population, and dominated SeMeCos after ~3 weeks (~250 generations) of sequential respotting onto solid minimal medium every 2 days (Extended Data Fig. 4b). Despite bearing the synthesis costs for H, L, U and M for the entire community, it was hence the prototrophs that obtained a slightly, but substantially increased growth rate over the auxotrophs in the common metabolic environment.

Next, we employed a highly sensitive targeted liquid chromatography-selected reaction monitoring (LC-SRM)-based metabolomics method^39^ to measure the concentrations of amino acids and uracil in cell pellets, as well as in the exometabolome surrounding prototrophic WT and SeMeCos (Fig. 2b). We found that, despite communities being forced to exchange only four metabolites (H, L, U and M), they exhibited a broad spectrum of metabolite concentration changes in both the intra- and the extracellular metabolome. In SeMeCos, in which two-thirds of the cells were auxotrophic for H, L, U or M (Fig. 2c), 14 out of 20 extracellular metabolite concentrations (amino acids and uracil) were significantly increased (Fig. 2c). Together, these results revealed that the presence of auxotrophs broadly changes metabolism in these communities and results in higher extracellular metabolite levels (Fig. 2c).

**Extended Data Fig. 5:**
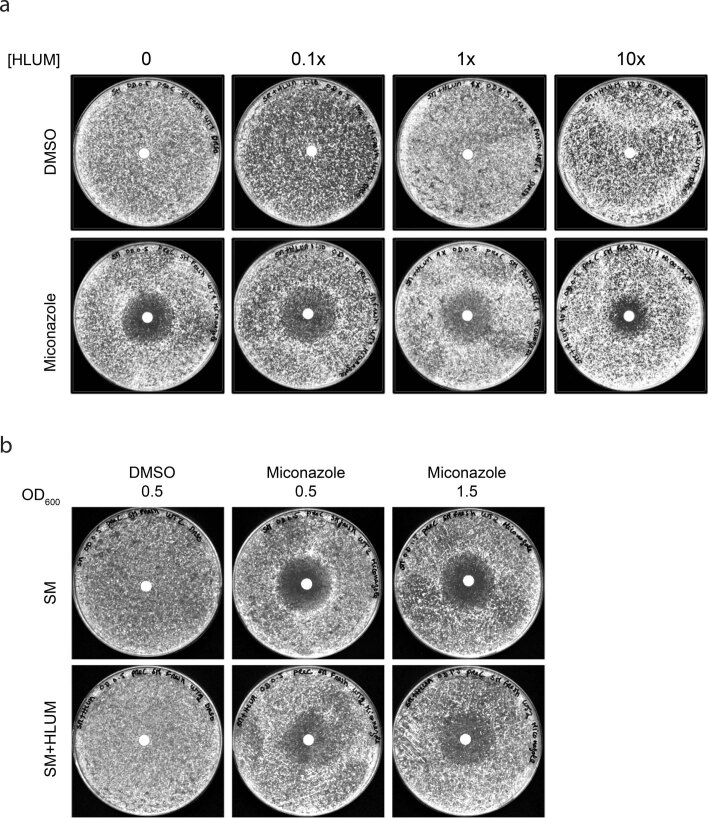
Rich extracellular metabolic environments protect growth of prototrophic yeast against antifungals independently of growth rate. (**a**) Drug response, as measured with a disk diffusion assay (DDA), in prototrophic yeast communities in synthetic minimal media (SM) supplemented with increasing concentrations of HLUM and treated with miconazole. DDAs were generated from wild-type cultures plated onto the respective media and then exposed to the azoles. Increasing HLUM supplementation reduced the inhibition zone (IZ) and increased cellular growth in the IZ in response to azole treatment. Data is n = 1 culture. **b**) Growth response to antifungals, as measured with a disk diffusion assay (DDA) in wild-type colonies in minimal (SM) media supplemented at different growth phases: initial exponential phase (OD600 = 0.5) and late exponential phase (OD600 = 1.5) treated with miconazole. Data are n = 1 wild-type culture. Source data

Our previous work has shown that cells import at least some extracellular amino acids at much higher concentrations than that required for growth. Such harvesting of metabolites can promote stress tolerance^18,40^. This situation suggested that the observed changes in the exometabolome could be associated with the observed increase in drug robustness. To test this hypothesis, we exposed WT cells to H, L, U and M under drug treatment. The four metabolites were supplied at growth-supporting concentrations, which resulted in similar uptake rates in both auxotrophs and prototrophs so that their flux distribution was similar^41,42^. We then measured the drug response against azole antifungals, using both disk diffusion assays (DDA) in solid media and assessment of MIC in liquid cultures via microbroth dilution assays. Nutrient supplementation markedly increased growth in the presence of azoles in WT cells, to the extent that the growth-inhibitory properties of azoles were largely abrogated (Fig. 2d,e). This phenotype was independent of the growth-promoting effects of supplementation, because AUC values did not substantially change in the untreated controls. Furthermore, this result was substantiated by a gain in tolerance and resistance against azoles with increasing concentration of the supplemented metabolites in WT cells (Extended Data Fig. 5a), in a growth-rate-independent manner (Extended Data Fig. 5b). Together, these results show that an increase in exometabolome metabolite concentration, as caused by the presence of auxotrophs, increases cellular ability to tolerate drugs.

### Reciprocity of the metabolic response in prototrophs

**Fig. 3:**
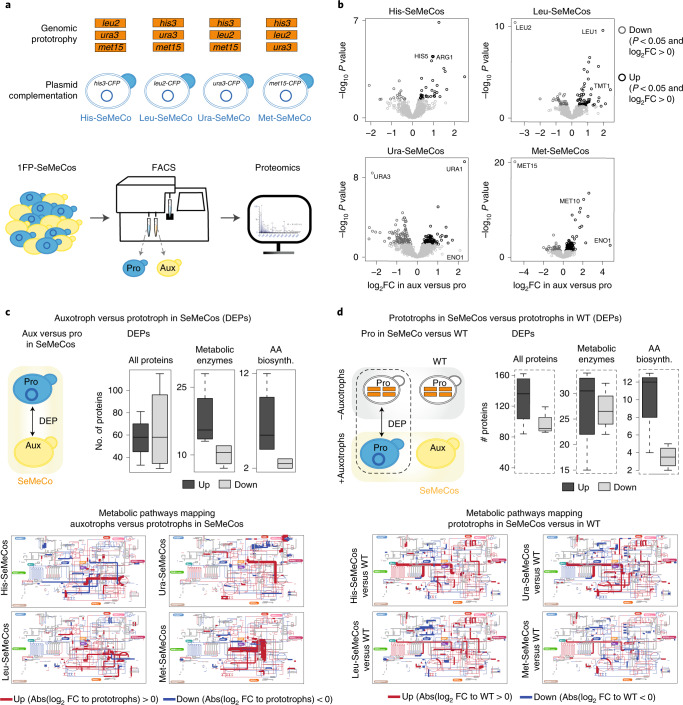
The prototroph proteome responds to the presence of auxotrophs. **a**, Schematic representation of auxotrophic SeMeCos strains where prototrophy is restored genomically, and in which the indicated plasmids are marked with CFP. Prototrophic and auxotrophic populations were sorted according to CFP expression. **b**, Proteomic analysis of sorted auxotrophic versus prototrophic cells in SeMeCos; *n* = 16 independent sorting experiments (*n* = 4 independent experiments for each of the auxotrophic SeMeCos strains), with differentially expressed proteins (DEP) illustrated as volcano plots. **c**, Top left: proteomic analysis of auxotrophs relative to cogrowing prototrophs in SeMeCos. Top right: summary of differentially expressed proteins and metabolic enzymes participating in amino acid biosynthesis; *n* = 16, whereby four bioreplicates of each of the four auxotrophic populations are compared to each other. Comparisons were made exclusively between proteins significantly differentially expressed in **b** where *P* < 0.05. Bottom: differential expression (log_2_ FC) of metabolic enzymes in auxotrophs relative to prototrophs in SeMeCos, mapped to the yeast metabolic network using iPATH3 (ref. ^83^); *n* = 4. **d**, Top left: proteomic analysis of prototrophic cells isolated from SeMeCos relative to prototrophs grown on their own. Top right: summary of differentially expressed proteins and metabolic enzymes in amino acid biosynthesis; *n* = 16, whereby four bioreplicates of each of the four prototrophic populations in SeMeCos and in WT communities were compared with each other. Comparisons were made exclusively between proteins significantly differentially expressed in **b** where *P* < 0.05. Bottom: differential protein expression (log_2_ FC) of enzymes in prototrophic cells cogrowing with auxotrophs (in SeMeCos) and metabolic enzyme expression relative to prototrophic cells cogrowing in WT communities mapped to the yeast metabolic network using iPATH3 (ref. ^83^); *n* = 4. **c**,**d,** Boxplots represent median (50% quantile (middle line) and lower and upper quantiles (lower (25% quantile) and upper (75% quantile), respectively) for the number of differentially expressed proteins. A protein is considered DEP when *P* < 0.05 (moderated *t*-test, two-sided) and log FC <0 or >0, downregulated or upregulated, respectively. Abs, absolute. Source data

**Extended Data Fig. 6:**
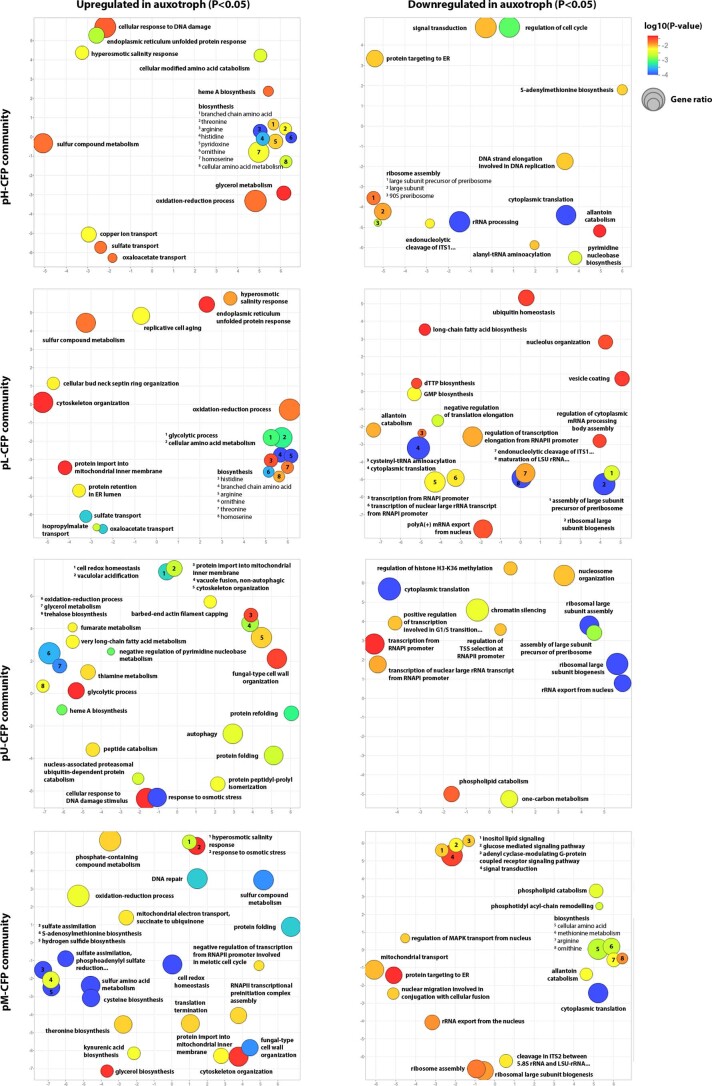
GO cluster representation in co-growing auxotrophs vs prototrophs. GO cluster and Gene Set Enrichment Analysis (GSEA) using the proteomics data from the sorted auxotrophic and prototrophic subpopulations, derived from single-plasmid CFP SeMeCos. Data are from n = 16 independent SeMecos sorting experiments (n = 4 independent experiments for each single-plasmid complemented strains (pH-, pL-, pU- and pM-SeMeCos). P-values obtained from GSEA were used to calculate a simRel score, a functional similarity measure for comparing two GO terms with each other and projected onto two dimensional space (x and y-axes) derived by applying multidimensional scaling to a matrix of the GO terms’ semantic similarities that generates slimmed GO terms. Left and right columns indicate upregulated and downregulated proteins respectively in auxotrophs when compared to the corresponding prototroph. Significance threshold is defined as P < 0.05. Bubble colour indicates log10(P-value), with blue being highly significant and red being less significant. Bubble sizes indicate gene ratio, which is the frequency of representation of the GO term within the Uniprot database for S. cerevisiae. A larger circle would indicate a more general term compared with a smaller circle that indicates a more specific term. All plots were generated via REVIGO^4^. Source data

**Extended Data Fig. 7:**
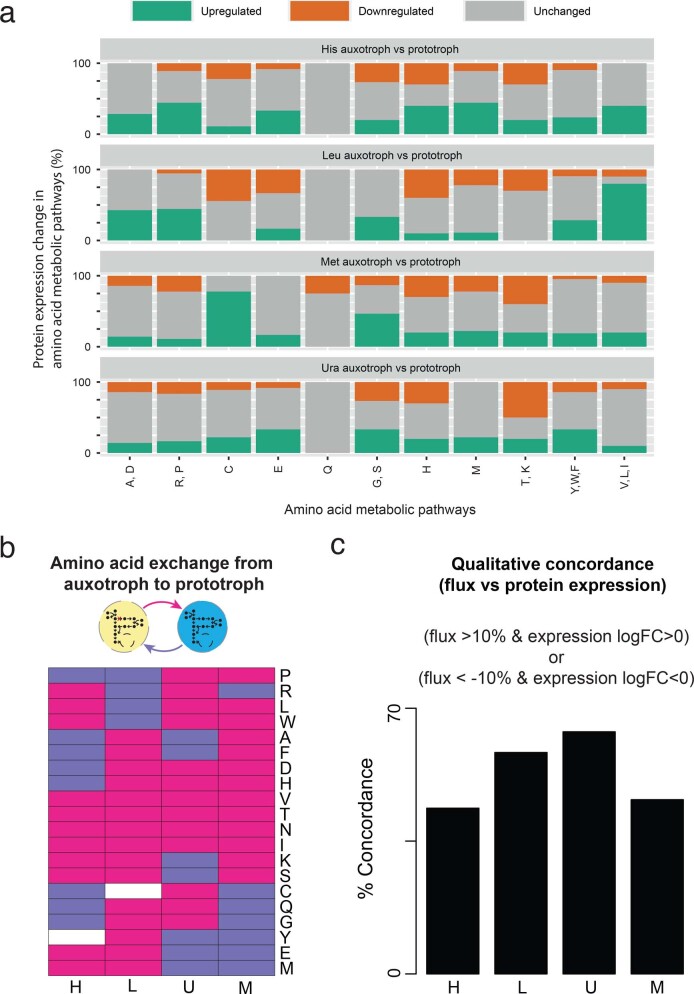
Auxotrophs export more non-essential metabolites and generate a rich metabolic environment conducive for drug tolerance. (**a**) Percentage change in expression of proteins involved in different amino acid biosynthetic pathways in the sorted histidine, leucine, uracil and methionine auxotrophs. Amino acid pathway annotations were taken as per the metabolic model, iMM904. (**b**) FBA analysis assessing amino acid exchange in auxotrophic versus protrophic subpopulations. The FBA analysis indicates that with the increased export of metabolites from auxotrophs, the degree of metabolite exchange within communal cells increases. Pink indicates export of metabolites from auxotrophs, blue indicates export of metabolites from prototrophs. (**c**) Qualitative analysis of concordance between fluxes (FBA analysis) and protein expression (proteomics analysis) in the auxotrophic versus prototrophic subpopulations. Source data

**Extended Data Fig. 8:**
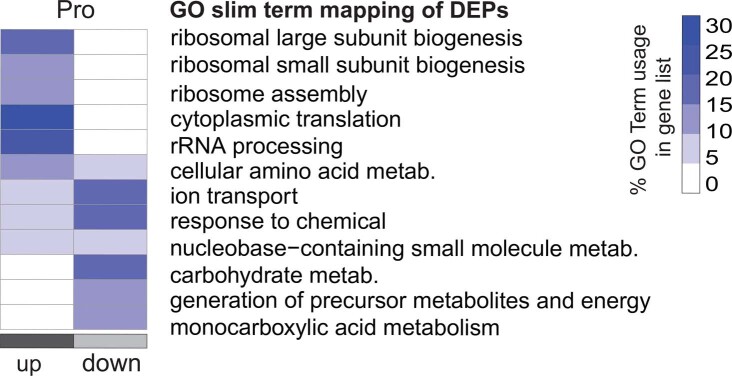
Prototrophs respond to the presence of auxotrophs and upregulate growth related processes. GO slim term mapping, using SGD’s GO Slim Mapper tool, of differentially expressed proteins from the proteomics analysis as in Fig. 3d, when prototrophs grow in the presence (SeMeCo communities) or absence (wild-type communities) of auxotrophs. Source data

Microbes in general, and yeast cells in particular, possess elaborate capacities to sense and uptake extracellular metabolites^10,11,29,43^. This biological situation implies that altered metabolite concentrations, as measured in the exometabolome (Fig. 2b), might trigger a metabolic response not only in auxotrophs but in all cells in the community. We generated SeMeCos containing only one segregating plasmid (pH, pL, pU or pM) encoding for an independently expressed enhanced cyan fluorescent protein (eCFP) (Fig. 3a). We then separated auxotrophic and prototrophic cells by FACS and measured their proteomes. In parallel, we measured the proteomes of equally treated prototrophs that had grown among themselves—that is, similarly treated cells isolated from prototrophic WT yeast colonies (Fig. 3a). We used liquid chromatography-sequential window aquisition of all theoretical ion spectra-mass spectrometry, a data-independent mass spectrometry acquisition technique to measure proteomes, in a pipeline we recently developed^44^ that provides a comprehensive, system-scale view of the state of the yeast metabolic network^45^. The proteome data thus obtained confirmed that CFP-based sorting of SeMeCos successfully separated auxotrophic and prototrophic populations based on expression of marker enzymes Leu2p, Met15p and Ura3p (His3p was below the detection limit in all samples). With this proteomic method, we quantified about 1,500 of the 4,000–5,000 proteins expressed in a typical yeast cell, covering mostly the high-abundant fraction of the proteome that is enriched for metabolic enzymes, including Leu2p, Ura3p and Met15p^34^. These marker enzymes were identified as the proteins most highly differentially expressed between the two populations (Fig. 3b). Gene set enrichment and Gene Ontology (GO) analyses of differentially expressed proteins revealed that metabolic terms or processes, particularly amino acid biosynthesis, were enriched among differentially expressed proteins (Extended Data Fig. 6). Multiple enzymes associated with flux changes in FBA (Fig. 2a) were expressed at lower levels in prototrophs compared to auxotrophs (Fig. 3c). Furthermore, also in agreement with the FBA analysis (Fig. 2a), metabolic pathways with a higher predicted flux in auxotrophs versus prototrophs (for example, V, L and I biosynthesis) contained many enzymes that were more highly expressed in auxotrophs. Similarly, many of the metabolic pathways with a lower predicted flux in prototrophs also had a higher proportion of downregulated enzymes (Extended Data Fig. 7a,b). Overall, when comparing flux predictions from FBA analysis with proteomic data, we detected that, depending on conditions, 44–64% of enzymes encoding for a reaction with flux change >10% were also differentially expressed (Extended Data Fig. 7c). Considering that overall, about half of these yeast metabolic changes can be explained by enzyme abundance changes^45^, this result indicates agreement between the predicted changes in flux and measured changes in the proteome. Next, we directly compared the proteomes of prototrophs that cogrow in the presence of auxotrophs—that is, in the SeMeCos environment—with those isolated from fully genomically prototrophic colonies—that is, WT communities (Fig. 3d). We found that enzyme expression, in particular enzymes implicated in amino acid biosynthetic pathways, were significantly different in prototrophs growing in the presence of auxotrophs, compared with prototrophic cells growing among prototrophic cells (Fig. 3d). Of note, while more pathways were upregulated in communal prototrophic cells others were downregulated, indicating that prototrophs both contribute and consume metabolites within SeMeCos. Finally, we also observed a higher expression of ribosomal and other growth-regulated proteins (Extended Data Fig. 8). This is also consistent with the aforementioned observation that prototrophs, despite carrying the synthesis costs for H, L, U and M, maintain a slightly faster growth rate compared to SeMeCos, providing no antifungal is present (Extended Data Fig. 4b).

### High metabolite efflux confers azole tolerance in auxotrophs

**Fig. 4:**
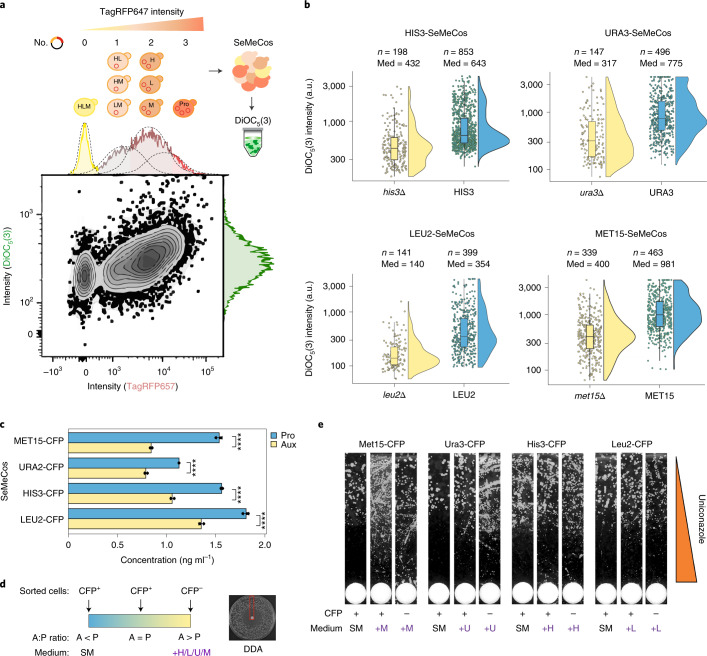
Increased metabolite export activity in auxotrophic cells promotes drug tolerance. **a**, SeMeCos cultures were incubated with DiOC_5_(3), and flow cytometry was used to assess fluorescent populations as described. Prototrophs, single and double auxotrophs (red gradient) and triple-auxotrophic (yellow) populations in SeMeCos were gated by TagRFP657 fluorescence. Gaussian curve fitting and cluster assignment via mclust^88^ identified subpopulations carrying different levels of auxotrophy. Prototrophic populations proportionally retain, on average, greater dye fluorescence, indicating a slower export of DiOC_5_(3). *n* = 20,000 cells from a single culture, of which 6,330 live cells were taken for downstream analysis. H, L and M indicate auxotrophy for histidine, leucine and methionine, respectively; PRO, prototrophic subpopulation. **b**, SeMeCos cultures were incubated with DiOC_5_(3), fixed and analyzed via fluorescence microscopy. Prototrophs and auxotrophs were identified by fluorescence, with two populations corresponding to low and high dye retention identified. Boxplots represent median (50% quantile (middle line), lower (25%) and upper (75%) quantiles, respectively); *n* = total number of cells counted per population; med, median fluorescence intensity per cell. Data are from one independent experiment. **c**, Intracellular concentration of uniconazole in auxotrophic and prototrophic subpopulations as sorted from singly auxotrophic SeMeCos, and measured by LC–MS/MS. Mean ± s.d. from a single injection from *n* = 3 biological replicates. Statistical significance was determined using a two-sided Student’s *t*-test, ****P < 0.00005. Exact *P* values are available in Source Data 4. **d**, Plating of auxotrophic and prototrophic subpopulations from sorted single auxotrophic SeMeCos onto SM or SM-supplemented medium with the complementary amino acids (+H/L/U/M) permitted titration of A:P ratios. Cropped DDA denoted by red-bounded region. **e**, DDA for the four sorted SeMeCos strains exposed to uniconazole plated onto SM or SM + H/L/U/M. DDAs were generated from a single-sort experiment, plated and exposed to uniconazole. a.u., Arbitrary units. Source data

**Extended Data Fig. 9:**
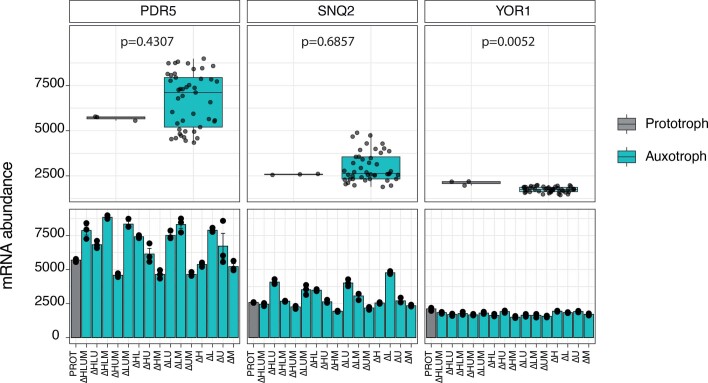
Expression of multidrug plasma membrane ABC transporters in auxotrophs and prototrophs for HLUM. mRNA expression profiles for the ABC transporters PDR5, SNQ2, and YOR1 were obtained from RNASeq expression data of n = 3 exponentially grown cultures, of 16 strains (1 prototroph and 15 auxotrophs in all possible combinations of HLUM) at similar cell density (optical density at 600 nm (OD600) of 0.8), followed by mRNA sequencing^5^. Analysis was performed either by (top) grouping prototrophs and auxotrophs normalised mRNA expression levels (box plots represent median (50% quantile (middle line) and lower and upper quantiles (lower (25% quantile) and upper (75% quantile); statistical significance was determined using a one-sided Kruskal-Wallis rank sum test) or (bottom) by each background strain (bar plots represent mean ± SEM of 3 independent cultures per strain, dots refer to individual cultures normalised mRNA abundance; statistical significance was determined using a two-sided Wilcoxon Rank Sum test, precise p-values are available in Source Data for Extended Data Fig. S9). The raw data^5^ (gene-wise read counts for gene expression estimation) was then processed using ‘DEseq2’^6^ in R for normalization. Source data

**Extended Data Fig. 10:**
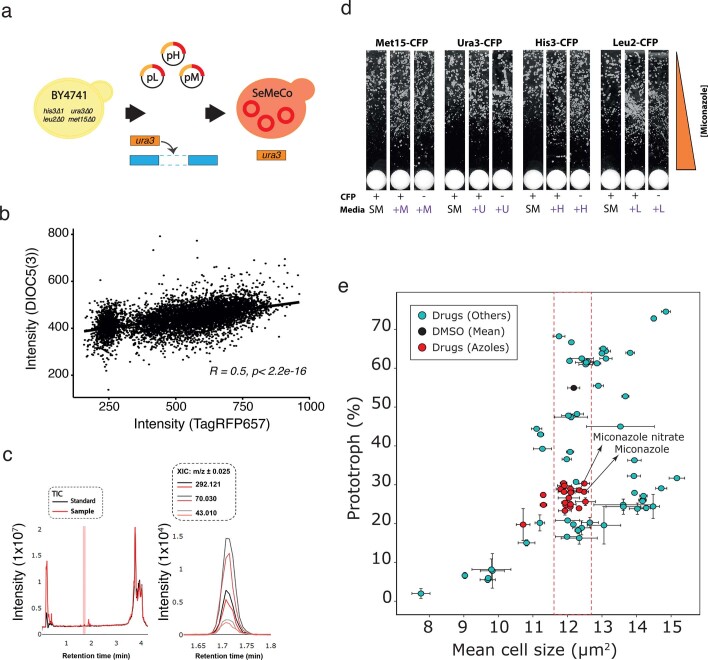
Auxotrophy correlates with DIOC_5_(3) export and azole tolerance independent of cell size in SeMeCos. (**a**) Construction of the 3-plasmid, TagRFP657 fluorescent SeMeCo strain. Auxotrophy was repaired in the parental BY4741 strain via plasmid complementation with pH, pL and pM (all encoding TagRFP657) and genomic knock-in of URA3. (**b**) Scatter plot of DIOC5(3) against TagRFP657 fluorescence intensity in 20,000 cells. Correlation was tested using a two-sided, Spearman’s Rank Correlation Coefficient (P value < 2.2e-16) with a R-value of 0.53 indicating a positive correlation. (**c**) Total ion chromatograms (TIC) and extracted ion chromatograms (XIC) corresponding to uniconazole in standard and cell pellet extracts from sorted cells. Peaks at <1 and ~4 min retention time correspond to highly hydrophilic and hydrophobic metabolites respectively. XIC at m/z of 292.121 was used to calculate concentration of uniconazole in extracts from a standard curve generated from the analytical standard (Sigma, 37044). Fragments at m/z 70.030 and 43.010 correspond to protonated triazole and loss of CNH thereof respectively. (**d**) DDA for all four sorted single-plasmid-CFP SeMeCos exposed to miconazole plated onto SM or SM + H/L/U/M. DDAs were generated from a single sort experiment and exposed to miconazole. (**e**) Summary of the changes in cell size against SeMeCo composition change as measured in the drug screen. Cell size was defined as the pixel area covered by a cell capture via high-throughput microscopy. DMSO (mean) indicates the global mean value from all DMSO treated wells. Drugs (Azoles) indicate azoles within the 900-FDA compounds collection, dotted lines indicate range of cell size changes for azole treated cells. Error bars indicate standard deviation of prototroph percentage or cell size from 3 biological replicates. Data are presented as mean values + /- SD. Source data

The concentration of extracellular metabolites is dependent on transport across the plasma membrane. In yeast, the export of amino acids is driven to a large extent by metabolite transporters with a broad substrate spectrum, and which are also responsible for the export of drugs and xenobiotics, including azole antifungals^46–49^. Indeed, a mechanism that mediates tolerance and resistance to antifungal substances is increased drug efflux^50^. We speculated that the increased export of amino acids from auxotrophs into the community space (Fig. 2c) might explain a higher tolerance to antifungal substances, if the higher efflux activity also affects drug concentrations. We first explored a transcriptome dataset that we previously used to quantify the impact of auxotrophy on gene expression epistasis^42^. We found that two out of three plasma membrane ATP-binding cassette (ABC) transporters with relevant antifungal activity (PDR5 and SNQ2)^51^ were expressed at higher levels across many auxotrophs, in comparison to prototrophs (Extended Data Fig. 9; ref. ^42^). To quantify export activity we then applied DiOC_5_(3), a cationic carbocyanine dye used to monitor general export activity, to SeMeCos. DiOC_5_(3) passively diffuses into cells, its export being dependent on ABC/MFS transporter activity. Therefore, cells with higher export activity have reduced staining for DiOC_5_(3)^52^. We assessed DiOC_5_(3) fluorescence intensity by flow cytometry, using a SeMeCos strain with three auxotrophies (*his3∆*, *leu2∆* and *met15∆*) complemented with three plasmids all encoding the same far-red-excitable TagRFP657 protein (Extended Data Fig. 10a). In this situation auxotrophy is, on average, inversely proportional to the intensity of fluorescence that is related to the number of plasmids carried by each cell (Fig. 4a). This experiment allowed us to quantitatively assess the relationship between auxotrophy and dye uptake in a complex SeMeCos system with multiple auxotrophies. Fluorescent marker and DiOC_5_(3) fluorescence levels were positively correlated, indicating that the prototrophic subpopulation exports the dye more slowly than the auxotrophic (Spearman’s rank order coefficient = 2.2 × 10^–16^, *R* = 0.53) (Fig. 4a and Extended Data Fig. 10b). To confirm that it was indeed the auxotrophs that exported the dye more rapidly, we used single auxotrophic, eCFP-expressing SeMeCos strains, stained them with DiOC_5_(3) and analyzed dye intensity by fluorescence microscopy. These analyses also revealed lower relative mean fluorescence intensity across the auxotrophic population compared to the prototrophic (Fig. 4b). Hence auxotrophs maintain lower DiOC_5_(3) concentrations, which indicates greater export activity when compared to prototrophs. In parallel, we sought to determine the influence of the drug on cell size, which needs to be taken into account when considering the underlying mechanisms for a change in drug resilience and/or transport. Reanalysis of the SeMeCos drug screen data revealed that, although some drugs can influence cell size, this was not the case for the majority of azoles tested (Extended Data Fig. 10e).

Next, we established an LC–MS method to quantify the intracellular concentration of uniconazole (Extended Data Fig. 10c) in sorted CFP^–^ auxotrophic and CFP^+^ prototrophic cells from azole-treated SeMeCos. Intracellular concentrations of uniconazole were significantly lower in auxotrophic subpopulations relative to prototrophic, sorted from the four communities (Fig. 4c). In each case, auxotrophs had lower azole levels than the corresponding prototrophs. We further noted that the absolute values of azoles were lower in *URA3* and *MET15* auxotrophs, followed by *HIS3* and *LEU2*, respectively, which corresponds to their differences in drug tolerance (Fig. 4c). Taking nondrug-treated cultures, we then sorted and plated the subpopulations onto either minimal (SM) medium, where only prototrophic cells can grow, or the corresponding supplemented medium (+H/L/U/M), which supports the growth of both populations, and assessed drug tolerance against miconazole or uniconazole using DDAs (Fig. 4d). Indeed, *ura3Δ* and *met15Δ cells* containing lower uniconazole concentrations when sorted from SeMeCos did grow better in the presence of the azole, as did *his3Δ* followed by *leu2Δ* cells, retaining higher azole concentrations when isolated from the same community (Fig. 4e). Similar results were observed in miconazole-treated cells: indeed, the resilience of *his3Δ and leu2Δ* auxotrophs was much stronger, highlighting the effect of differing azole potency on drug tolerance (Extended Data Fig. 10d).

## Discussion

Microbial cells generally produce, release, take up and sense a broad range of metabolites and, when microbes grow together, these intrinsic metabolic properties result in a high degree of metabolite exchange. Indeed, for many metabolites, prototrophic microbes prioritize uptake from the exometabolome over their own biosynthetic capacity for growth. Accumulating evidence suggests that the degree of metabolite exchange within cells in communities is extensive, with quantitative metabolome data revealing high levels of exported metabolites that enrich the exometabolome of both single- and multispecies communities^29,53–56^. The high degree of metabolite availability within microbial communities is reflected by a high prevalence of auxotrophic cells^57,58^ that can grow only if the community environment contains growth-supporting concentrations of the metabolites essential to them.

There is still an intense debate over how such high frequencies of auxotrophs can persist within communities without being at a disadvantage^17,58^. One popular explanation for the success of auxotrophy is the ‘black queen hypothesis’^59^, which postulates that cells profit either from auxotrophy, by reducing the burden of costly metabolite synthesis, or from the situation where cells are incapable of privatizing their resources once exported. A conundrum around the success of auxotrophs is, however, the ‘cheater dilemma’, because auxotrophs can exploit prototrophs that provide metabolites without returning any benefit^60^. If such a benefit allowed auxotrophs to grow more rapidly than prototrophs, it would ultimately destabilize the community^61,62^. Another possible explanation for the relatively stable coexistence observed in communities is that prototrophs might simply export or leak ‘costless’ metabolites. In this case the auxotrophic cells, even if cheaters, might impose minimal costs to the community because the metabolites essential to auxotroph growth are regarded as waste products by the prototrophs^63^.

The results presented here add a new aspect to understanding the success of auxotrophs in microbial communities. Our findings demonstrate that, when taking specific metabolites from the community, auxotrophs broadly reconfigure their metabolism and overflow metabolites other than those taken up. As a consequence, the presence of auxotrophs increases metabolite concentration in the community environment. We further show that such changes in the extracellular environment can have a profound effect on communities because microbial cells, irrespective of whether they are prototrophs or auxotrophs, sense changes in the extracellular metabolome and adapt their metabolism accordingly^21,42^. We found evidence for a reciprocal response, in which prototrophs downregulated several metabolic enzymes in the presence of auxotrophs, indicating that they made use of metabolites released by the auxotrophs. A community that contains auxotrophic cells therefore has broadly altered metabolic properties.

Auxotrophs possess the same basic metabolic network structure as prototrophs, and the interconnectivity in this metabolic network explains the increased overflow of a broad range of unrelated metabolites, when cells shift from amino acid self-synthesis to uptake. Indeed, we see that in the presence of the metabolite outside of cells, WT cells uptake metabolites like auxotrophs^29^ and, throughout our experiments, we find that their metabolism is reconfigured accordingly. From this situation we arrived at the conclusion that the ability to uptake metabolites for efficient use of the exometabolome is a property of the microbial cell, and hence needs no secondary adaptation to come into effect. Indeed, cells that are members of natural communities overflow large amounts of metabolites, an example being a community of lactic acid bacteria and yeasts^64^. Corroborating this, the discovery of increased drug tolerance was made in the genetic auxotrophs and was replicated in genetically prototrophic cells with induced metabolic uptake. We would like to emphasize that, because of these metabolic properties, the gain in tolerance can be explained by the individual cell’s optimization of metabolism and does not require coevolution of auxotrophs and prototrophs to be beneficial. Indeed, the basis for metabolite exchange interactions relies on the basic metabolic properties of microbes, in particular their ability to feedback inhibit their intrinsic biosynthetic pathways for efficient uptake and exploitation of extracellular metabolites, while it is reconfigurations in the metabolic network that are responsible for altered overflow metabolism^9^. The latter changes are explained by the structure of the metabolic network, which largely relies on the thermodynamics and reaction properties of the interconverted metabolites. Due to high interconnectivity, fluxes change broadly when cells switch from self-synthesis to uptake of a metabolite^42^.

Eventually, our results unveiled a biochemical mechanism linking metabolic interactions to robustness against antimicrobial drug treatments. By analysis of data collected as part of the EMP, as well as extensive gut microbiome data^27,28^ and confirmed by the traceable SeMeCo model, we found that amino acid auxotrophs are highly prevalent and more resilient to a broad range of drug exposures than prototrophs. We provide evidence that the underlying mechanism is increased metabolite efflux activity resulting from the metabolic reconfiguration undergone by cells when they switch from self-synthesis of specific essential metabolites to their uptake. This raises the attractive prospect of a priori drug efficacy prediction, although further characterization of drug efflux pump structure and activity will be required to achieve this. The data indicate that increased drug tolerance is an emergent property that comes as a consequence of metabolite exchange interactions, where the degree of metabolite exchange is more complex than can be determined simply by the number of auxotrophs present. Our metabolome data, FBA model and proteome data show that each of the different metabolites (H, L, U and M) used to model auxotroph–prototroph interactions has a different impact on a broad range of metabolites and proteomes, and each of the metabolite titrations has a different quantitative effect on drug levels and resistance. Thus, increased robustness to drugs is a function of metabolite exchange activity between cells that is stimulated by the presence of auxotrophs according to their degree of interactions with other cells.

Although we have not focused on the evolutionary aspects of resistance in our manuscript, it is also worth discussing our findings in the context of multiple reports that have attributed metabolic interactions to the emergence of drug resistance^25^. Metabolic interactions can drive community structures that are important for the spread of drug-resistance genes^65^. Moreover, the evolution of antimicrobial resistance can originate from tolerant subpopulations of cells that grow slowly in otherwise inhibitory drug concentrations^22,66–68^, at which point resistance genes can spread rapidly in complex communities via horizontal gene transfer^69–73^. Our data imply that in communities in which the presence of auxotrophs stimulates a high degree of metabolic interactions, the effective population size of cells that can persist following drug treatment is increased, possibly accelerating adaptive evolution. This speculation is consistent with recent reports showing that an increased number of metabolic mutations following metabolic evolution assays leads to increased drug resistance in bacteria^74^.

## Methods

### Yeast cultivation and growth assays

#### Plasmids and strain construction

All details relating to strains and plasmids used in this study can be found in Supplementary Information. Prototrophy was restored either by genomic knock-in or plasmid complementation^75^, following standard techniques^76^.

### SeMeCos generation and culture

The generation and culture of SeMeCos was based on previous work^19^. SeMeCos starts with a single prototrophic founder cell, where between one and four genetic auxotrophies are complemented by plasmids containing the essential metabolic gene(s) and, optionally, encode for a fluorescent marker gene (mRuby2, mWasabi, eCFP and TagRFP657). Because the SeMeCo founder cell grows into a colony, an increasing number of cells stochastically segregate one or more of its plasmids. This gives progeny to auxotrophic subpopulations that continue to grow by obtaining essential metabolites from complementary prototrophic cells. After the rapid emergence of auxotrophic subpopulations, cells enter an equilibrium of metabolite exchange with cogrowing prototrophs^29^. Cryostocks were streaked onto yeast nitrogen broth (6.8 g l^–1^, Sigma) + glucose (20 g l^–1^, Sigma) + 2% agar medium (solid Spizizen minimal (SM) medium) and cultured at 30 °C for 2–3 days. A microcolony was then diluted in 200 µl of distilled water (dH_2_O) and normalized to OD_600_ = 0.8. Then, 5 µl was spotted onto solid SM medium to generate a giant colony, corresponding to ~7.2 × 10^4^ cells using a predefined OD-to-cell number standard curve. Cells were incubated for 2 days at 30 °C, then giant colony generation was repeated twice. This dilution and respotting was performed to ensure that cells had undergone sufficient proliferation cycles and plasmid segregation to enable metabolic cooperation whilst being continuously maintained in an exponential growth phase, preventing nutrient recycling from dead or dying cells. For the competitive assay, giant colony generation was repeated nine times, forming every 2 days for 18 days, corresponding to ~120 doublings in total. For culture, giant spots were diluted in 1 ml of dH_2_O and normalized to OD_600_ = 0.1 in SM liquid medium. This relatively high starting OD_600_ ensured that cells were kept at a density that minimizes disturbing the relative proportions of auxotrophs and prototrophs generated in SeMeCos. Cells were then incubated overnight (~20 h) at 30 °C and 180 r.p.m and collected for downstream experiments.

### Metabotyping by colony-forming units and sequencing

A sample of the SeMeCos culture was cryostocked at days 6, 12 and 18, from which new giant colonies were generated by resuspending scrapings in 30 µl of dH_2_O and spotting 5 µl of this dilution onto SM solid medium. This colony was resuspended in 1 ml of dH_2_O, plated at 1:100,000 dilution on solid synthetic complete (SC) medium and incubated at 30 °C for 2 days to establish colony-forming units (CFUs). Each CFU was then resuspended in 100 µl of dH_2_O in a 96-well plate (Nunc, Sigma) and replica plated onto SM and SC + 5-fluoroorotic acid (5-FOA, 1 mg ml^–1^; Sigma) + H/L/U/M solid medium. Auxotrophies could be distinguished based on the presence or absence of URA3; cells carrying pU and pHLUM do not survive in the presence of 5-FOA, as opposed to those carrying pH, L or M. Plasmid extraction and sequencing were then performed for additional confirmation.

### FACS and flow cytometry of SeMeCos

Fluorescent-tagged SeMeCos were generated and cultured as described above. Before sorting, cells were sonicated for 30 s to dissociate clumps and stained with LIVE/DEAD Fixable dye UV or far-red excitable (ThermoFisher, catalog nos. L23105 and L34973, respectively) to identify live cells. Cells were sorted or analyzed on a BD Aria Fusion or Fortessa HTS-X20 (BD Biosciences) using the following fluorophore and excitation laser: bandpass filter settings: LIVE/DEAD dye; 355-nm-UV; 440/40-UV, eCFP; 405-nm-violet; 525/50-violet, mWasabi/DiOC_5_(3); 488-nm-blue; 530/30-blue, mRuby2; 561-nm-yellow/green; 610/20-yellow, TagRFP657; 633 nm-red; 730/45-red. Analysis was conducted in either BD FACSDiva v.8.0 or FlowJo v.10.6.2.

### Fluorescent SeMeCos composition analysis and drug screening

For high-throughput drug screening, a preculture of four-plasmid-bearing fluorescent SeMeCo was grown overnight to OD_600_ = 1.0 and diluted to OD_600_ = 0.3. Next, 384-well microtiter plates were predosed with 0.7 µl per well of drug at 1 mM stock concentration using a Labcyte Echo 550 acoustic dispenser, for a final working concentration of 10 µM. Diluted culture (70 µl per well) was then transferred using a rapid liquid dispensing robot (FluidX Xrd-384) and incubated for 24 h. Cells were fixed rapidly by the addition of 20 µl of 16% paraformaldehyde before transfer to polylysine-coated imaging plates (Perkin Elmer Cell Carrier-384 Ultra, catalog no. 6057300) for high-throughput fluorescence imaging using a Perkin Elmer Opera Phenix fully automated confocal microscope. Thresholding and autofluorescence corrections were performed with the aid of the corresponding single auxotrophic fluorescent strains (pH-mRuby2, pL-mWasabi, pU-eCFP and pM-TagRFP657), and image analysis was performed via the high-content imaging software Harmony v.5.0. Drugs were subsetted from an FDA-approved collection from Selleck (2,572 compounds, no. L1300-Z368434-100uL).

For low-throughput validation, SeMeCo cultures were propagated as described as in SeMeCos generation and culture and transferred to 1,200-µl deep-well plates (Greiner Bio-One), then diluted to final OD_600_ = 0.3 in 1 ml of liquid medium (SM). Drugs (atorvastatin, fluvastatin, itraconazole, miconazole, uniconazole—all Selleck) were reconstituted in DMSO (Sigma) to a stock concentration of either 1 or 10 mM and used at a final concentration of 10 µM. Plates were incubated at 30 °C on an orbital shaker for 24 h, before 200 µl of each culture was transferred to a U-bottomed, high-throughput, flow-cytometry-compatible microtiter plate for data acquisition. Analysis was performed on FlowJo v.10.6.2, from which raw cell counts were extracted, processed in R^77^ and visualized in Clustvis v.1.0 (ref. ^78^).

### Metabolomics

#### Sample preparation

Wild-type and 4p-SeMeCos were grown to exponential phase, as described in SeMeCos generation and culture, and 0.5 ml of each culture was collected for amino acid and uracil profiling. Samples were centrifuged at 4,000*g* for 3 min, and supernatants (SN) were transferred to a new microcentrifuge tube for extracellular metabolite profiling while cell pellets were used for intracellular metabolite profiling. Both cell pellets and SN were immediately frozen in dry ice and samples stored at −80 °C until required for analysis. Amino acid and uracil extraction, separation and detection protocols were adapted from ref. ^39^. Briefly, 200 µl of 80% ethanol at 80 °C was added to the yeast pellets; samples were then heated for 2 min at 80 °C, vigorously mixed on a vortex mixer and incubated for a further 2 min at 80 °C followed by vigorous vortexing. The extracts were removed from debris by centrifugation at 12,000*g* for 5 min. SN were also centrifuged at 12,000*g* for 5 min, to further purify samples from any debris. Before analysis by high-performance liquid chromatography–tandem mass spectrometry (HPLC–MS/MS), the order of samples was randomized and during analysis a quality control sample was assessed every 24 samples.

#### Sample acquisition

Analysis by LC–MS/MS was based on previous work^39^. Amino acids and uracil were separated by hydrophilic interaction liquid chromatography using an ACQUITY UPLC BEH amide column (130 A˚, 1.7 mm, 2.1 × 100 mm^2^) on a liquid chromatography (Agilent 1290 Infinity) and tandem mass spectrometry (Agilent 6460) system. Buffer A was composed of 50:50 acetonitrile (ACN)/water (Greyhound, nos. Bio-012041 and 23214125), 10 mM ammonium formate (Fluka, catalog no. 14266), 0.176% formic acid (FA; Fluka, catalog no. O6454); buffer B consisted of 95:5:5 ACN/methanol/water (Greyhound, no. BIO-13684102), 10 mM ammonium formate and 0.176% FA. Gradient elution was performed at a constant flow rate of 0.9 ml min^–1^. Starting conditions were 85% buffer B then, after 0.7 min the concentration was decreased gradually to 5% until 2.5 min and kept for a further 0.05 min before returning to initial conditions. The column was then equilibrated, resulting in a total run time of 3.25 min. Compounds were identified by matching retention time and fragmentation (MS2) with commercially obtained standards (Sigma-Aldrich, catalog no. LAA21). Signals for free amino acids were then acquired in dynamic SRM mode in Agilent Technologies MassHunter software suite v.8.07.00. Amino acid and uracil quantifications were then normalized according to OD_600_ at the time of collection.

### Proteomics

#### Sample preparation

For proteomics on FACS samples, 20 million sorted WT (that had just passed through FACS), CFP^+^ and CFP^–^ SeMeCos were immediately spun down at 4,000 r.p.m., SN was partially discarded, 600 µl was transferred to a 1.5-ml centrifuge tube and spun down at 4,000 r.p.m. for 5 min, SN was discarded and cell pellets were then immediately stored at −80 °C until all samples had been collected. Cell pellets were processed in a bead beater for 5 min at 1,500 r.p.m. (Spex Geno/Grinder) in a lysis buffer, where proteins were denatured in 8 M urea (Sigma-Aldrich, no. 33247) and 0.1 M ammonium bicarbonate (Sigma-Aldrich, no. 09830) at pH 8.0. Samples were spun down for 1 min at 4,000 r.p.m before being reduced in 5 mM dithiothreitol (Sigma-Aldrich, no. 43815) for 1 h at 30 °C. Samples were then alkylated in 10 mM iodoacetamide (Sigma-Aldrich, no. I1149) for 30 min at room temperature and protected from light. Samples were diluted to <1.5 M urea in 0.1 M ammonium bicarbonate at pH 8.0, before overnight digestion of proteins at 37 °C with trypsin (Promega, no. V511X). Trypsin was neutralized with 1% FA (Fisher Scientific, no. 13454279), before peptides were purified in 96-well MacroSpin plates (Nest Group): (1) plates were first equilibrated in a series of methanol (1×) (Greyhound Chromatography, no. BIO-13684102), 50% ACN (2×) (Greyhound Chromatography, no. Bio-012041-2.5 L) and 3% ACN/0.1% FA (2×); between each wash, plates were spun down for 1 min at 100*g* and flowthrough was discarded. (2) Samples were loaded into plates and peptides were cleaned up in a series of 3% ACN and 0.1% FA (3x); between each wash, samples were spun down for 1 min at 100*g* and flowthrough was discarded. (3) Peptides were eluted into a new collection plate with 50% ACN (3×) and spin-dried overnight at room temperature in a speed vacuum. Peptides were then dissolved in 40 µl of 3% ACN/0.1% FA. Peptide concentration was measured at an absorbance of 280 nm using Lunatic (Unchained Labs).

#### Sample acquisition

Acquisition was largely based on a previous study^44^. In brief, digested peptides were analyzed on a nanoAcquity (Waters) (running as 5 µl min^−1^ microflow liquid chromatography) coupled to a TripleTOF 6600 (SCIEX). Protein digest (2 µg) was injected and peptides were separated with a 23-min nonlinear gradient starting with 4% ACN in 0.1% FA and increasing to 36% ACN in 0.1% FA. A Waters HSS T3 column (150 mm × 300 µm, 1.8-µm particles) was used. The data-independent acquisition (DIA) method consisted of a single MS1 scan at 400–1,250 *m/z* (50-ms accumulation time) and 40 MS2 scans (35-ms accumulation time) with a variable precursor isolation width covering the mass range from 400 to 1,250 *m/z*.

#### Data analysis

Data quantification was performed using DIA-NN v.7.1 software^79^. Postprocessing data analysis was conducted in R. Missing values in proteomics data were median imputed. Differential protein expression analysis was performed using the limma package v.3.48.3 in R^80^. GO terms were retrieved using the package ‘GO2ALLORFS object of org.Sc.sgd.db’ v.3.14.0 (ref. ^81^), and enrichment analysis of differentially expressed proteins was performed using hypergeometric statistical tests. The GO slim-term mapper from the SGD database^82^ was used to map differentially expressed proteins with GO slim terms. Metabolic enzyme expression levels were mapped to the yeast metabolic network using iPATH3 (ref. ^83^).

### Community modeling

The genome-scale metabolic model of *S. cerevisiae* (*iMM904_NADcorrected*) was used to perform model simulations^36,37^. The model was modified by the addition of two transport reactions (R_NADPtru: nadp_c -> nadp_r and R_NADPHtru: nadph_c -> nadph_r) to reproduce ergosterol auxotrophy. Furthermore, the URAt2 reaction was changed from nonreversible to reversible since uracil was previously shown to be secreted by the cell to the extracellular environment^29^. The revised model, which consisted of a total of 1,577 reactions (1,413 metabolic and 164 exchange reactions), was then utilized to construct the auxotrophic–prototrophic community metabolic models using the compartment-per-guild approach^84^. In this approach, both strains in the community are treated as separate compartments where exchange metabolites are transported to and from the extra compartment pool of metabolites. All 1,413 metabolic reactions were assigned to both strains, and suffixes (PROTS for prototroph or AUXOS for auxotrophs) were added to all metabolites and reactions to avoid any duplication between the two strains. For instance, glucokinase reaction ID, GLUK, was renamed R_PROTSGLUK and glucose compound ID was changed to M_PROTSCglc_D for the prototroph, whereas for the auxotroph these were changed to R_AUXOSGLUK and M_AUXOSCglc_D, respectively. Extracellular metabolites/reactions were kept the same and shared by both strains. A new reaction, representing the community biomass (AUXOSCbiomass + PROTSCbiomass → community biomass), was also added to the model. The final community model consisted of 2,991 reactions (1,413 × 2 metabolic, 164 exchange and one community biomass). Growth of the community (community biomass reaction) was maximized in minimal media. Because the auxotrophic strain lacks an essential gene, it cannot make its biomass in minimal media on its own. However, due to metabolic cooperation between the auxotroph and prototroph, the former acquires essential compounds allowing the community as a whole to grow. Default minimal media were used, and the uptake flux of glucose and oxygen was doubled to feed the two strains. Percentage concordance between the predicted flux and protein expression profile was calculated by dividing the number of reactions, where either (1) flux change >10% and protein expression log fold change (FC) >0 or (2) flux change <−10% and protein expression log FC < 0, by the total number of reactions with absolute flux change >10%. For each reaction, protein expression log FC (auxotroph versus prototroph) was assigned using the gene–protein–reaction relation, where ‘or’ and ‘and’ logic was replaced by max and min, respectively. Model simulations were performed using the cobra toolbox^85^.

### Biochemical assays

#### DiOC_5_(3) export assay

For SeMeCos, giant colony cultures were grown overnight and washed 3× with dH_2_O before incubation with 2 µM DiOC_5_(3) (Stratech) at 30 °C for 30 min with agitation. Cells were then incubated for a further 1 h in SM to allow export of the dye before fixation with 4% PFA (Sigma) for 20 min and mounting for fluorescence microscopy. For three-plasmid-bearing fluorescent SeMeCos, giant colonies were cultured in the presence of uniconazole (10 µM, Selleck) before washing and incubation with 2 µM DiOC_5_(3) at 30 °C for 30 min with agitation. Here, the fixation step was omitted and cells were taken directly for flow cytometry analysis.

#### DDA

SeMeCos were generated and cultured as in SeMeCos generation and culture. Following sorting of ~10 million cells, cultures were centrifuged and resuspended in 700 µl of SM, of which 100 µl was spread on solid medium using glass beads. For WT strains and experiments that did not require sorting, precultures were normalized to OD_600_ = 1.0 before plating. Once plates were dry, a single blank Oxoid antimicrobial susceptibility disk (ThermoFisher) was placed in the center of the plate and inoculated with 50 µg of uniconazole or miconazole (Selleck). Plates were then incubated for 3 days at 30 °C and imaged.

#### Azole quantification by LC–MS

##### Sample preparation

Glass beads and 200 µl of methanol were added to aliquots of previously sorted cells (7.5 million of CFP^+^ and CFP^–^ cells from each single auxotrophic fluorescent strain). Samples were beaten for 3 × 3 min at 250*g*, followed by centrifugation (1 min, maximum speed) before further centrifugation for 3 min at 1,250*g* through a 0.45-µm filter plate (Agilent). Samples were then evaporated to dryness at room temperature using an Eppendorf Concentrator Plus (Eppendorf). The dry residue was reconstituted in 40% methanol (1 ml) and an aliquot subjected to LC–MS analysis.

#### Sample acquisition

Liquid chromatography was performed on an Infinity II ultra-high-pressure system (Agilent) hyphenated to a TripleTOF 6600 mass spectrometer (Sciex). Chromatographic separations were performed on a C18 ZORBAX Rapid Resolution High Definition (RRHD) column (2.1 × 50 mm^2^, 1.8 µm) (Agilent) by application of a linear gradient of 40–60% buffer B over 3 min at a flow rate of 600 µl min^–1^ (buffer A, 0.1% FA/H_2_O v/v; buffer B, 0.1% FA/ACN v/v). For washing of the column, the organic solvent was increased to 100% buffer B within 0.5 min. Equilibration time between runs was 3.6 min. For washing and equilibration, the flow rate was increased to 1 ml min^–1^. The injection volume was set to 20 µl and the column temperature held at 30 °C. The mass spectrometer was operated in positive ESI mode using a DuoSpray ion source, and spray voltage was set to 5.5 kV. Gas flows of 50 arbitrary units for the nebulizer gas, 40 arbitrary units for the heater gas and 25 arbitrary units for the curtain gas were employed. The temperature of the turbo gas was adjusted to 450 °C. A duty cycle consisted of a single MS scan (accumulation time, 250 ms; scan range, 50–800 *m/z*) followed by a product ion scan at 292.121 *m/z* in high-sensitivity mode (accumulation time, 100 ms; collision energy, 30 eV with a spread of 5 eV; scan range, 20–300 *m/z*). Instruments were controlled by Analyst TF 1.7.1 software (Sciex). Compound identification and quantification were achieved using MultiQuant 3.0.2 (Sciex). Identification was based on chromatographic retention time and compound-specific ion traces of product ions (70.030 and 43.010 *m/z*), as well as the precursor ion (292.121 *m/z*). Ion traces were extracted at a width of 0.05 *m/z*. Quantification was performed using external calibration at the MS1 level due to the absence of interfering compounds. The linear calibration model covered a range of 0.05−2.5 ng ml^–1^, with 1/x weighting and the lowest level being considered the lower level of quantitation.

### Reanalysis of metagenomic EMP dataset

Area under the curve growth values, as measured by optical density at absorbance wavelength OD_600_, were obtained from a study that screened the effect of 1,197 drugs on 40 gut microbiome members in vitro^28^. In the original study the AUC values were normalized and scaled such that a value of 1 corresponded to no growth effect of drug on the microbe, while a value of 0 corresponded to no growth of the microbe under the drug. Gut microbiome members were assigned as being either auxotrophic or prototrophic depending on the presence or absence of any amino acid auxotrophy, based on in silico predictions from another recent study^30^. In short, as described in Machado et al.^86^, genome-scale models were used to calculate the auxotrophies of all reference species. Then, with 100 stochastic character mapping, posterior probabilities of the auxotrophic ancestral state were calculated using functions from the phytools R package^87^. All data analysis was carried out in R v.3.6.1 (unless otherwise described), using the package tidyverse 1.3.0 for data manipulation and visualization.

### Reporting Summary

Further information on research design is available in the Nature Research Reporting Summary linked to this article.

## Supplementary information

Supplementary Information Supplementary Fig. 1: Flow cytometry gating strategy related to Fig. 4a, and supplementary references related to Extended Data Figs. 1–3, 6 and 9

Reporting Summary

Peer Review Information

Supplementary Table 1 Tables listing the strains, plasmids and primers used used to clone and restore prototrophy in the BY4741 haploid yeast strain.

## Data Availability

The data supporting the findings of this study are available within the paper and its Supplementary Information and are deposited within publicly accessible repositories. Datasets derived from EMP relevant to Fig. 1 and Extended Data Fig. 1 can be accessed at https://qiita.ucsd.edu/. The proteomic datasets generated during the current study and that are relevant to the data shown in Fig. 3 and Extended Data Figs. 6–8 are available from the PRoteomics IDEntifications database (PRIDE, https://www.ebi.ac.uk/pride/, project ID: PXD031160). Yeast gene functions and the GO slim-term mapper can be accessed at the Saccharomyces Genome Database (https://www.yeastgenome.org/). Protein sequence databases used for the identification and mapping of proteins from proteomics can be accessed via KEGG (https://www.genome.jp/kegg/pathway.html) and Uniprot (https://www.uniprot.org/), respectively. Source data are provided with this paper.

## Source data

Source Data Fig. 1 Statistical source data.
Source Data Fig. 2 Statistical source data.
Source Data Fig. 2 Unprocessed images for Supplementary Fig. 2a,b.
Source Data Fig. 3 Statistical source data.
Source Data Fig. 4 Statistical source data.
Source Data Fig. 4 Unprocessed images for Fig. 4e.
Source Data Extended Data Fig. 1 Statistical source data.
Source Data Extended Data Fig. 2. Unprocessed images for Supplementary Fig. 2a,b.
Source Data Extended Data Fig. 3 Statistical source data.
Source Data Extended Data Fig. 4 Statistical source data.
Source Data Extended Data Fig. 5 Unprocessed images for Supplementary Fig. 5a,b.
Source Data Extended Data Fig. 6 Statistical source data.
Source Data Extended Data Fig. 7 Statistical source data.
Source Data Extended Data Fig. 8 Statistical source data.
Source Data Extended Data Fig. 9 Statistical source data.
Source Data Extended Data Fig. 10 Statistical source data.
Source Data Extended Data Fig. 10 Unprocessed images for Supplementary Fig. 10d.

## Code availability

No custom codes were generated as part of this study. All analyses conducted in R v.3.6.1 used standard, publicly accessible packages obtained either through GitHub (https://github.com/), the Comprehensive R Archive Network (CRAN, https://cran.r-project.org/) or Bioconductor (https://www.bioconductor.org/).
