# Supplementary material for: Microbial communities form rich extracellular metabolomes that foster metabolic interactions and promote drug tolerance
Source: Nat Microbiol. 2022 Mar 21;7(4):542–55. doi: 10.1038/s41564-022-01072-5 (PMC8975748; doi:10.1038/s41564-022-01072-5)
Supplement: Supplementary file 1 — Supplementary Fig. 1: Flow cytometry gating strategy related to Fig. 4a, and supplementary references related to Extended Data Figs. 1–3, 6 and 9 [file 41564_2022_1072_MOESM1_ESM.pdf]

nature portfolio

https://doi.org/10.1038/s41564-022-01072-5

## Supplementary information

# Microbial communities form rich extracellular metabolomes that foster metabolic interactions and promote drug tolerance

In the format provided by the authors and unedited

# Supplementary Information

**Microbial communities form rich extracellular metabolomes that foster metabolic interactions and promote drug tolerance**

Jason S. L. Yu[1]†, Clara Correia-Melo[1]†, Francisco Zorrilla[2,3], Lucia Herrera-Dominguez[1,4,]‡, Mary Y. Wu[5], Johannes Hartl[4], Kate Campbell[6,11]§, Sonja Blasche[2,3], Anna-Sophia Egger[1], Marco Kreidl[1], Christoph B. Messner[1,6], Vadim Demichev[4], Anja Freiwald[4,7], Michael Muelleder[7], Michael Howell[5], Judith Berman[8], Kiran R. Patil[2,3], Mohammad Tauqeer Alam[9,10]*$, and Markus Ralser[1,4,6]*$

**This file includes:**

Supplementary figure 1: Gating strategy for Fig. 4a

Supplementary references related to Extended Data 1, 2, 3, 6 and 9.

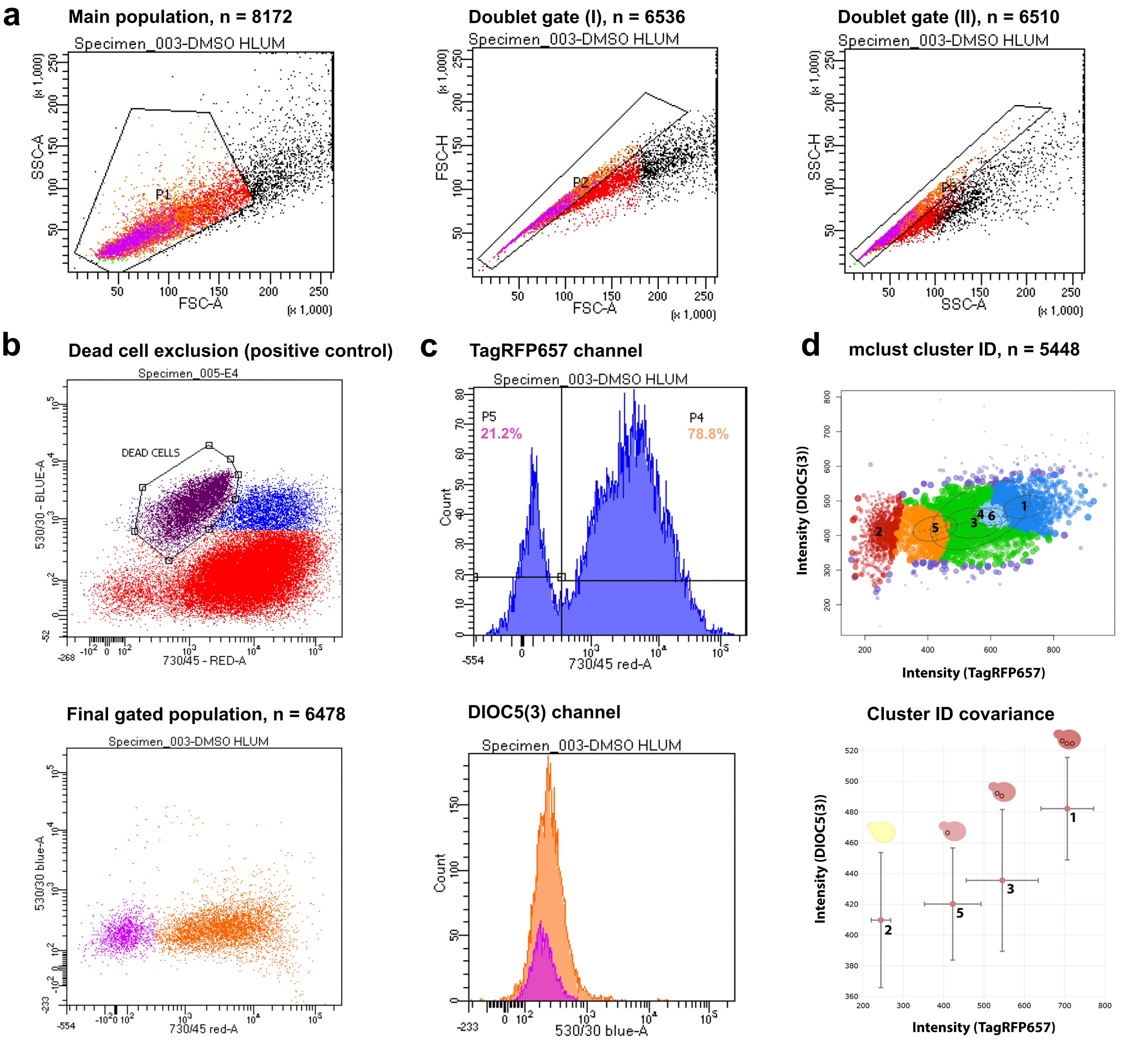


**Supplementary figure 1: Gating strategy for DIOC5(3) accumulation experiment (Fig. 4a) (a)** Gating strategy identifying main population and doublet discrimination using forward (FSC-A) and side (SSC-A) scatter area parameters. Gated populations as indicated by boxed areas (P1, P2, P3). **(b)** Positive dead-cell control (heat-treated yeast) used to establish dead-cell exclusion gate. Dead cells stain strongly for LIVE/DEAD fixable dye, have lower TagRFP657 expression due to denaturation of the fluorophore-core and high DIOC3(5) staining due disruption of dye export machinery. Final gated population contains relatively little dead cells by comparison. **(c)** Histograms showing distribution of final gated population across TagRFP657 and DIOC5(3) channels, TagRFP657+/- (P4, P5) populations could be easily identified. **(d)** Unsupervised Gaussian finite mixture modelling predicts the presence of 6 populations based on DIOC5(3) and TagRFP657 intensity/cell, of which 4 can be unambiguously identified (coloured, clusters 1, 2, 3 and 5). Analysis of covariances reveal that these clusters correspond well with the expected populations in the culture (full auxotroph, 1-3 plasmid bearing).

**Supplementary references**


1. Machado, D. *et al.* Polarization of microbial communities between competitive and cooperative metabolism. *Cold Spring Harbor Laboratory* 2020.01.28.922583 (2020) doi:10.1101/2020.01.28.922583.
2. Vowinckel, J., Hartl, J., Butler, R. & Ralser, M. MitoLoc: A method for the simultaneous quantification mitochondrial network morphology and membrane potential in single cells. *Mitochondrion* (2015) doi:10.1016/j.mito.2015.07.001.
3. Segrè, D., Vitkup, D. & Church, G. M. Analysis of optimality in natural and perturbed metabolic networks. *Proc. Natl. Acad. Sci. U. S. A.* **99**, 15112–15117 (2002).
4. Supek, F., Bošnjak, M., Škunca, N. & Šmuc, T. REVIGO Summarizes and Visualizes Long Lists of Gene Ontology Terms. *PLoS One* **6**, e21800 (2011).
5. Alam, M. T. *et al.* The metabolic background is a global player in Saccharomyces gene expression epistasis. *Nat Microbiol* **1**, 15030 (2016).
6. Love, M. I., Huber, W. & Anders, S. Moderated estimation of fold change and dispersion for RNA-seq data with DESeq2. *Genome Biol.* **15**, 550 (2014).
