# Supplementary material for: Microbial communities form rich extracellular metabolomes that foster metabolic interactions and promote drug tolerance
Source: Nat Microbiol. 2022 Mar 21;7(4):542–55. doi: 10.1038/s41564-022-01072-5 (PMC8975748; doi:10.1038/s41564-022-01072-5)
Supplement: Supplementary file 2 — Reporting Summary [file 41564_2022_1072_MOESM2_ESM.pdf]

nature research

Corresponding author(s): MT Alam, M Ralser

Last updated by author(s): January 2022

Reporting Summary

Nature Research wishes to improve the reproducibility of the work that we publish. This form provides structure for consistency and transparency in reporting. For further information on Nature Research policies, see our Editorial Policies and the Editorial Policy Checklist.

Statistics

For all statistical analyses, confirm that the following items are present in the figure legend, table legend, main text, or Methods section.

n/a	Confirmed	
☐	☒	The exact sample size (*n*) for each experimental group/condition, given as a discrete number and unit of measurement
☐	☒	A statement on whether measurements were taken from distinct samples or whether the same sample was measured repeatedly
☐	☒	The statistical test(s) used AND whether they are one- or two-sided<br>*Only common tests should be described solely by name; describe more complex techniques in the Methods section.*
☒	☐	A description of all covariates tested
☐	☒	A description of any assumptions or corrections, such as tests of normality and adjustment for multiple comparisons
☐	☒	A full description of the statistical parameters including central tendency (e.g. means) or other basic estimates (e.g. regression coefficient) AND variation (e.g. standard deviation) or associated estimates of uncertainty (e.g. confidence intervals)
☐	☒	For null hypothesis testing, the test statistic (e.g. *F*, *t*, *r*) with confidence intervals, effect sizes, degrees of freedom and *P* value noted<br>*Give P values as exact values whenever suitable.*
☒	☐	For Bayesian analysis, information on the choice of priors and Markov chain Monte Carlo settings
☒	☐	For hierarchical and complex designs, identification of the appropriate level for tests and full reporting of outcomes
☐	☒	Estimates of effect sizes (e.g. Cohen's *d*, Pearson's *r*), indicating how they were calculated

Our web collection on statistics for biologists contains articles on many of the points above.

Software and code

Policy information about availability of computer code

Data collection

BD FACSDiva 8.0, FlowJo 10.6.2, Harmony High-Content Imaging and Analysis Software 5.0, Agilent Technologies, Masshunter software suite 8.07.00, Sciex Analyst TF 1.7.1, Clustvis 1.0, Docker 20.10.7, R 3.6.1, DIA-NN 1.7.1, MATLAB 2018b, Cobra Toolbox 3.0, Sciex MultiQuant 3.0.2, limma 3.48.3, mClust 5.4.9, Revigo 1.0, iPATH 3.0, org.Sc.sgd.db v3.14.0.

Data analysis

BD FACSDiva 8.0, FlowJo 10.6.2, Harmony High-Content Imaging and Analysis Software 5.0, Agilent Technologies, Masshunter software suite 8.07.00, Sciex Analyst TF 1.7.1, Clustvis 1.0, Docker 20.10.7, R 3.6.1, DIA-NN 1.7.1, MATLAB 2018b, Cobra Toolbox 3.0, Sciex MultiQuant 3.0.2, limma 3.48.3, mClust 5.4.9, Revigo 1.0, iPATH 3.0, org.Sc.sgd.db v3.14.0.

Fluorescence based yeast assays: FACS and flow cytometry analysis was conducted in the BD FACSDiva v8.0 or FlowJo v10.6.2. Fluorescent SeMeCo composition analysis (flow cytometry) and high-throughput drug screening analysis (fluorescence microscopy) was performed on FlowJo v10.6.2 or Harmony High-Content Imaging and Analysis v5.0 software respectively. Further analysis and visualisation were generated using either R v3.6.1 or Clustvis v1.0 deployed natively using a Docker v20.10.7 container.

Metabolomics: Signals for free amino acids were acquired in dynamic SRM mode using Agilent MassHunter software suite v8.07.00. Data analysis was performed using R v3.6.1

Proteomics: Total peptide concentration was measured using the Lunatic UV/Vis spectrophotometer (Unchained Labs) via inbuilt software prior to analysis by microFlow-SWATH. Proteomes were acquired via the Sciex Analyst TF v1.7.1 software. and processed using DIA-NN v7.1. Post-processing data analysis was conducted in R. Differential protein expression analysis was performed using the limma package v3.48.3 in R. Gene ontology (GO) terms were retried using the org.Sc.sgd.db v3.14.0 package in R. GO slimming analysis was conducted either via the GO slim term mapper from the SGD database (https://www.yeastgenome.org/goSlimMapper) or via Revigo v1.0 (http://revigo.irb.hr/). Mapping of differentially expressed proteins was conducted using iPATH v3.0 (https://pathways.embl.de/) .

Community modelling: The iMM904_NAD corrected mathematical model was used and cobra toolbox v3.0, MATLAB 2018b software was used for analysis.

Intracellular drug quantification: Chromatographic separations were performed on a C18 XORBAX Rapid Resolution High Definition (RRHD) column from Agilent. The instruments were controlled by the Sciex Analyst TF v1.7.1 software. Compounds identification and quantification was conducted using Sciex MultiQuant v3.0.2, data analysis was done in R v3.6.1.

For manuscripts utilizing custom algorithms or software that are central to the research but not yet described in published literature, software must be made available to editors and reviewers. We strongly encourage code deposition in a community repository (e.g. GitHub). See the Nature Research guidelines for submitting code & software for further information.

Data

Policy information about availability of data

All manuscripts must include a data availability statement. This statement should provide the following information, where applicable:

- Accession codes, unique identifiers, or web links for publicly available datasets
- A list of figures that have associated raw data
- A description of any restrictions on data availability

The data supporting the findings of this study are available within the paper, its Supplementary Information and are deposited within publicly accessible repositories. Datasets derived from the Earth Microbiome Project (EMP) relevant to Figure 1 and Extended Data figure 1 can be accessed at https://qiita.ucsd.edu/. The proteomic datasets generated during the current study that are relevant to data shown in Figure 3 and Extended Data figures 6-8 are available from the PRoteomics IDEntifications database (PRIDE, https://www.ebi.ac.uk/pride/, project ID: PXD031160). Yeast gene functions and GO slim term mapper can be accessed at the Saccharomyces Genome Database (SGD, https://www.yeastgenome.org/). Protein sequence databases used for the identification and mapping of proteins from the proteomics can be accessed via KEGG (https://www.genome.jp/kegg/pathway.html) and Uniprot (https://www.uniprot.org/) respectively.

Field-specific reporting

Please select the one below that is the best fit for your research. If you are not sure, read the appropriate sections before making your selection.

☒ Life sciences ☐ Behavioural & social sciences ☐ Ecological, evolutionary & environmental sciences

For a reference copy of the document with all sections, see nature.com/documents/nr-reporting-summary-flat.pdf

Life sciences study design

All studies must disclose on these points even when the disclosure is negative.

Sample size	Sample sizes were chosen so that each individual experiment was sufficiently powered for the conclusions we derive. Statistical analyses are corrected for sample size and multiple testing where appropriate.
Data exclusions	For proteomics and metabolomics analysis, samples were considered outliers if they had less than 80% of the maximum identification number across all samples. Other than this, no data was excluded from all analyses.
Replication	Three biological/independent replicates were usually performed for each experiment unless otherwise stated in the figure captions. All replication attempts were successful.
Randomization	In analytical experiments all the samples were randomised. In all other experiments randomisation was not applied as the experiments were either performed in parallel, or we did not expect undue influence by acquisition order or plate/block position based on previous experience/publication where we apply similar methodologies.
Blinding	Measurements and analysis were not blinded as no observer bias is expected in this technical study.

Reporting for specific materials, systems and methods

We require information from authors about some types of materials, experimental systems and methods used in many studies. Here, indicate whether each material, system or method listed is relevant to your study. If you are not sure if a list item applies to your research, read the appropriate section before selecting a response.

Materials & experimental systems

n/a	Involved in the study	
☒	☐	Antibodies
☒	☐	Eukaryotic cell lines
☒	☐	Palaeontology and archaeology
☒	☐	Animals and other organisms
☒	☐	Human research participants
☒	☐	Clinical data
☒	☐	Dual use research of concern

Methods

n/a	Involved in the study	
☒	☐	ChIP-seq
☐	☒	Flow cytometry
☒	☐	MRI-based neuroimaging

Antibodies

Antibodies used	NA
Validation	NA

Eukaryotic cell lines

Policy information about cell lines

Cell line source(s)	No cell lines were used. The Saccharomyces cerevisiae strain BY4741 was obtained from EuroSCARF (Y00000).
Authentication	Genetic background of BY4741 (Δhis3, Δleu2, Δura3, Δmet15) was confirmed by sequencing and media growth tests.
Mycoplasma contamination	NA
Commonly misidentified lines (See ICLAC register)	NA

Palaeontology and Archaeology

Specimen provenance	*Provide provenance information for specimens and describe permits that were obtained for the work (including the name of the issuing authority, the date of issue, and any identifying information).*
Specimen deposition	*Indicate where the specimens have been deposited to permit free access by other researchers.*
Dating methods	*If new dates are provided, describe how they were obtained (e.g. collection, storage, sample pretreatment and measurement), where they were obtained (i.e. lab name), the calibration program and the protocol for quality assurance OR state that no new dates are provided.*

☐ Tick this box to confirm that the raw and calibrated dates are available in the paper or in Supplementary Information.

Ethics oversight	*Identify the organization(s) that approved or provided guidance on the study protocol, OR state that no ethical approval or guidance was required and explain why not.*

Note that full information on the approval of the study protocol must also be provided in the manuscript.

Animals and other organisms

Policy information about studies involving animals; ARRIVE guidelines recommended for reporting animal research

Laboratory animals	*For laboratory animals, report species, strain, sex and age OR state that the study did not involve laboratory animals.*
Wild animals	*Provide details on animals observed in or captured in the field; report species, sex and age where possible. Describe how animals were caught and transported and what happened to captive animals after the study (if killed, explain why and describe method; if released, say where and when) OR state that the study did not involve wild animals.*
Field-collected samples	*For laboratory work with field-collected samples, describe all relevant parameters such as housing, maintenance, temperature, photoperiod and end-of-experiment protocol OR state that the study did not involve samples collected from the field.*
Ethics oversight	*Identify the organization(s) that approved or provided guidance on the study protocol, OR state that no ethical approval or guidance was required and explain why not.*

Note that full information on the approval of the study protocol must also be provided in the manuscript.

Human research participants

Policy information about studies involving human research participants

Population characteristics	*Describe the covariate-relevant population characteristics of the human research participants (e.g. age, gender, genotypic information, past and current diagnosis and treatment categories). If you filled out the behavioural & social sciences study design questions and have nothing to add here, write "See above."*
Recruitment	*Describe how participants were recruited. Outline any potential self-selection bias or other biases that may be present and how these are likely to impact results.*
Ethics oversight	*Identify the organization(s) that approved the study protocol.*

Note that full information on the approval of the study protocol must also be provided in the manuscript.

Clinical data

Policy information about clinical studies

All manuscripts should comply with the ICMJE guidelines for publication of clinical research and a completed CONSORT checklist must be included with all submissions.

Clinical trial registration	*Provide the trial registration number from ClinicalTrials.gov or an equivalent agency.*

Study protocol	*Note where the full trial protocol can be accessed OR if not available, explain why.*
Data collection	*Describe the settings and locales of data collection, noting the time periods of recruitment and data collection.*
Outcomes	*Describe how you pre-defined primary and secondary outcome measures and how you assessed these measures.*

Dual use research of concern

Policy information about dual use research of concern

Hazards

Could the accidental, deliberate or reckless misuse of agents or technologies generated in the work, or the application of information presented in the manuscript, pose a threat to:

No	Yes	
☒	☐	Public health
☒	☐	National security
☒	☐	Crops and/or livestock
☒	☐	Ecosystems
☒	☐	Any other significant area

Experiments of concern

Does the work involve any of these experiments of concern:

No	Yes	
☒	☐	Demonstrate how to render a vaccine ineffective
☒	☐	Confer resistance to therapeutically useful antibiotics or antiviral agents
☒	☐	Enhance the virulence of a pathogen or render a nonpathogen virulent
☐	☐	Increase transmissibility of a pathogen
☒	☐	Alter the host range of a pathogen
☒	☐	Enable evasion of diagnostic/detection modalities
☒	☐	Enable the weaponization of a biological agent or toxin
☒	☐	Any other potentially harmful combination of experiments and agents

ChIP-seq

Data deposition

☐ Confirm that both raw and final processed data have been deposited in a public database such as GEO.

☐ Confirm that you have deposited or provided access to graph files (e.g. BED files) for the called peaks.

Data access links<br>*May remain private before publication.*	*For "Initial submission" or "Revised version" documents, provide reviewer access links. For your "Final submission" document, provide a link to the deposited data.*
Files in database submission	*Provide a list of all files available in the database submission.*
Genome browser session<br>(e.g. UCSC)	*Provide a link to an anonymized genome browser session for "Initial submission" and "Revised version" documents only, to enable peer review. Write "no longer applicable" for "Final submission" documents.*

Methodology

Replicates	*Describe the experimental replicates, specifying number, type and replicate agreement.*
Sequencing depth	*Describe the sequencing depth for each experiment, providing the total number of reads, uniquely mapped reads, length of reads and whether they were paired- or single-end.*
Antibodies	*Describe the antibodies used for the ChIP-seq experiments; as applicable, provide supplier name, catalog number, clone name, and lot number.*
Peak calling parameters	*Specify the command line program and parameters used for read mapping and peak calling, including the ChIP, control and index files used.*

Data quality	*Describe the methods used to ensure data quality in full detail, including how many peaks are at FDR 5% and above 5-fold enrichment.*
Software	*Describe the software used to collect and analyze the ChIP-seq data. For custom code that has been deposited into a community repository, provide accession details.*

Flow Cytometry

Plots

Confirm that:

☒ The axis labels state the marker and fluorochrome used (e.g. CD4-FITC).

☒ The axis scales are clearly visible. Include numbers along axes only for bottom left plot of group (a 'group' is an analysis of identical markers).

☒ All plots are contour plots with outliers or pseudocolor plots.

☒ A numerical value for number of cells or percentage (with statistics) is provided.

Methodology

Sample preparation	Prior to FACS or flow cytometry cells were briefly sonicated to dissociate clumps and stained with LIVE/DEADTM Fixable dye UV to identify live cells.
Instrument	BD Fortessa X20B, BD FACSymphony
Software	BD FACSDivaTM (v8.0), FlowJo (v10.6.2), R v4.0.2, mclust package.
Cell population abundance	Not applicable as cells were only analysed and not sorted.
Gating strategy	A threshold exclusion of 5000 was set on the cytometer to omit debris. FSC-A vs SSC-A was then used to identify the main population. Double discrimination was performed using FSC-A vs FSC-H followed by SSC-A vs SSC-H. Live/Dead gating was conducted based on LIVE/DEAD Fixable dye UV staining and gating with positive dead-cell control. Resulting cells from this gating were used in downstream analysis to generate Fig. 4c.

☒ Tick this box to confirm that a figure exemplifying the gating strategy is provided in the Supplementary Information.

Magnetic resonance imaging

Experimental design

Design type	*Indicate task or resting state; event-related or block design.*
Design specifications	*Specify the number of blocks, trials or experimental units per session and/or subject, and specify the length of each trial or block (if trials are blocked) and interval between trials.*
Behavioral performance measures	*State number and/or type of variables recorded (e.g. correct button press, response time) and what statistics were used to establish that the subjects were performing the task as expected (e.g. mean, range, and/or standard deviation across subjects).*

Acquisition

Imaging type(s)	*Specify: functional, structural, diffusion, perfusion.*
Field strength	*Specify in Tesla*
Sequence & imaging parameters	*Specify the pulse sequence type (gradient echo, spin echo, etc.), imaging type (EPI, spiral, etc.), field of view, matrix size, slice thickness, orientation and TE/TR/flip angle.*
Area of acquisition	*State whether a whole brain scan was used OR define the area of acquisition, describing how the region was determined.*

Diffusion MRI ☐ Used ☐ Not used

Preprocessing

Preprocessing software	*Provide detail on software version and revision number and on specific parameters (model/functions, brain extraction, segmentation, smoothing kernel size, etc.).*
Normalization	*If data were normalized/standardized, describe the approach(es): specify linear or non-linear and define image types used for transformation OR indicate that data were not normalized and explain rationale for lack of normalization.*

Normalization template	*Describe the template used for normalization/transformation, specifying subject space or group standardized space (e.g. original Talairach, MNI305, ICBM152) OR indicate that the data were not normalized.*
Noise and artifact removal	*Describe your procedure(s) for artifact and structured noise removal, specifying motion parameters, tissue signals and physiological signals (heart rate, respiration).*
Volume censoring	*Define your software and/or method and criteria for volume censoring, and state the extent of such censoring.*

Statistical modeling & inference

Model type and settings	*Specify type (mass univariate, multivariate, RSA, predictive, etc.) and describe essential details of the model at the first and second levels (e.g. fixed, random or mixed effects; drift or auto-correlation).*
Effect(s) tested	*Define precise effect in terms of the task or stimulus conditions instead of psychological concepts and indicate whether ANOVA or factorial designs were used.*

Specify type of analysis: ☐ Whole brain ☐ ROI-based ☐ Both

Statistic type for inference (See Eklund et al. 2016)	*Specify voxel-wise or cluster-wise and report all relevant parameters for cluster-wise methods.*
Correction	*Describe the type of correction and how it is obtained for multiple comparisons (e.g. FWE, FDR, permutation or Monte Carlo).*

Models & analysis

n/a	Involved in the study
☐	☐ Functional and/or effective connectivity
☐	☐ Graph analysis
☐	☐ Multivariate modeling or predictive analysis

Functional and/or effective connectivity	*Report the measures of dependence used and the model details (e.g. Pearson correlation, partial correlation, mutual information).*
Graph analysis	*Report the dependent variable and connectivity measure, specifying weighted graph or binarized graph, subject- or group-level, and the global and/or node summaries used (e.g. clustering coefficient, efficiency, etc.).*
Multivariate modeling and predictive analysis	*Specify independent variables, features extraction and dimension reduction, model, training and evaluation metrics.*
