# Supplementary figures and images for: Microbial communities form rich extracellular metabolomes that foster metabolic interactions and promote drug tolerance
Source: Nat Microbiol. 2022 Mar 21;7(4):542–55. doi: 10.1038/s41564-022-01072-5 (PMC8975748; doi:10.1038/s41564-022-01072-5)

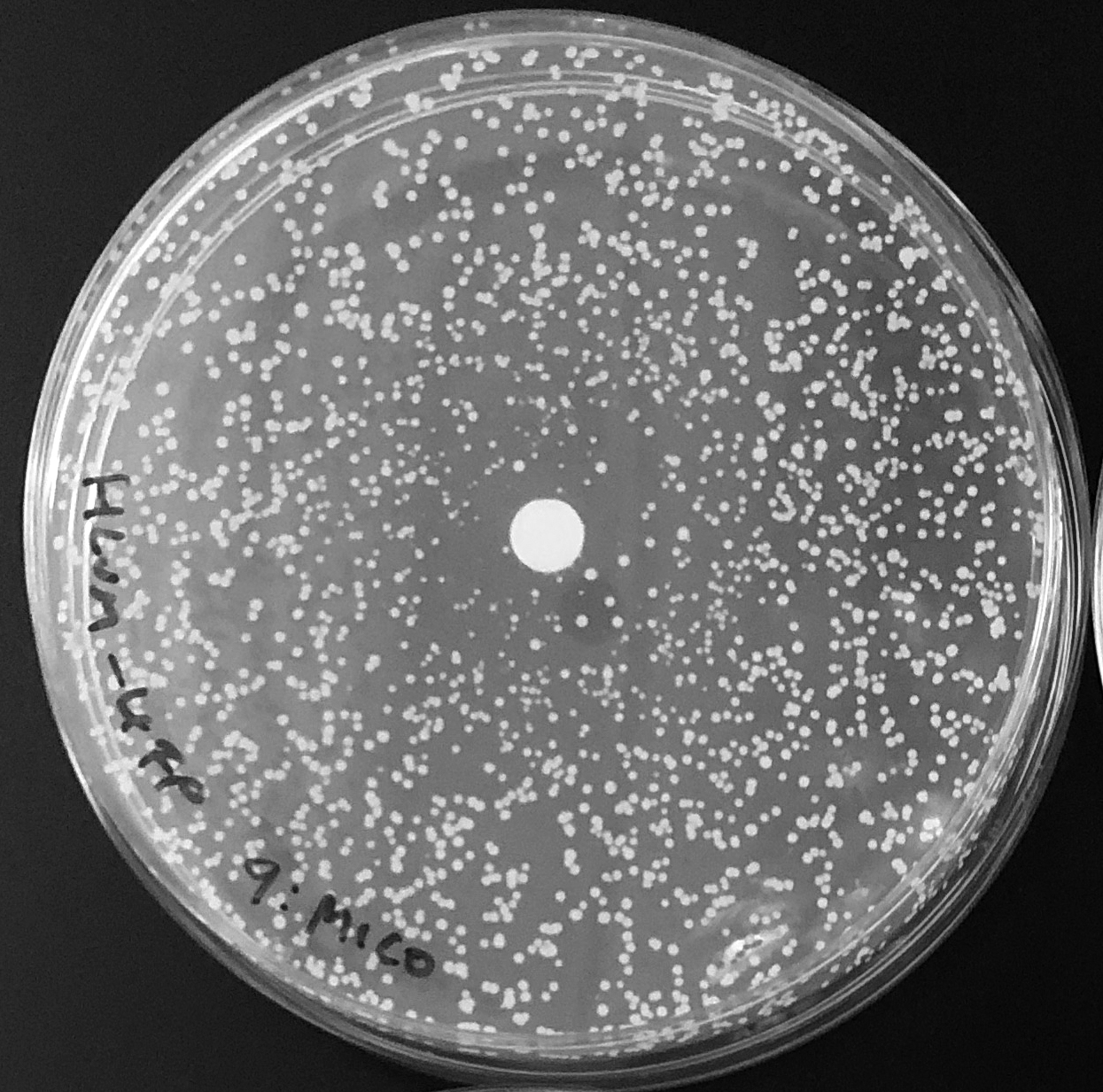

Supplement: Source Data Fig. 2 — Unprocessed images for Supplementary Fig. 2a,b. [file 41564_2022_1072_MOESM7_ESM.zip › MICO_4FP_HLUM.jpg]

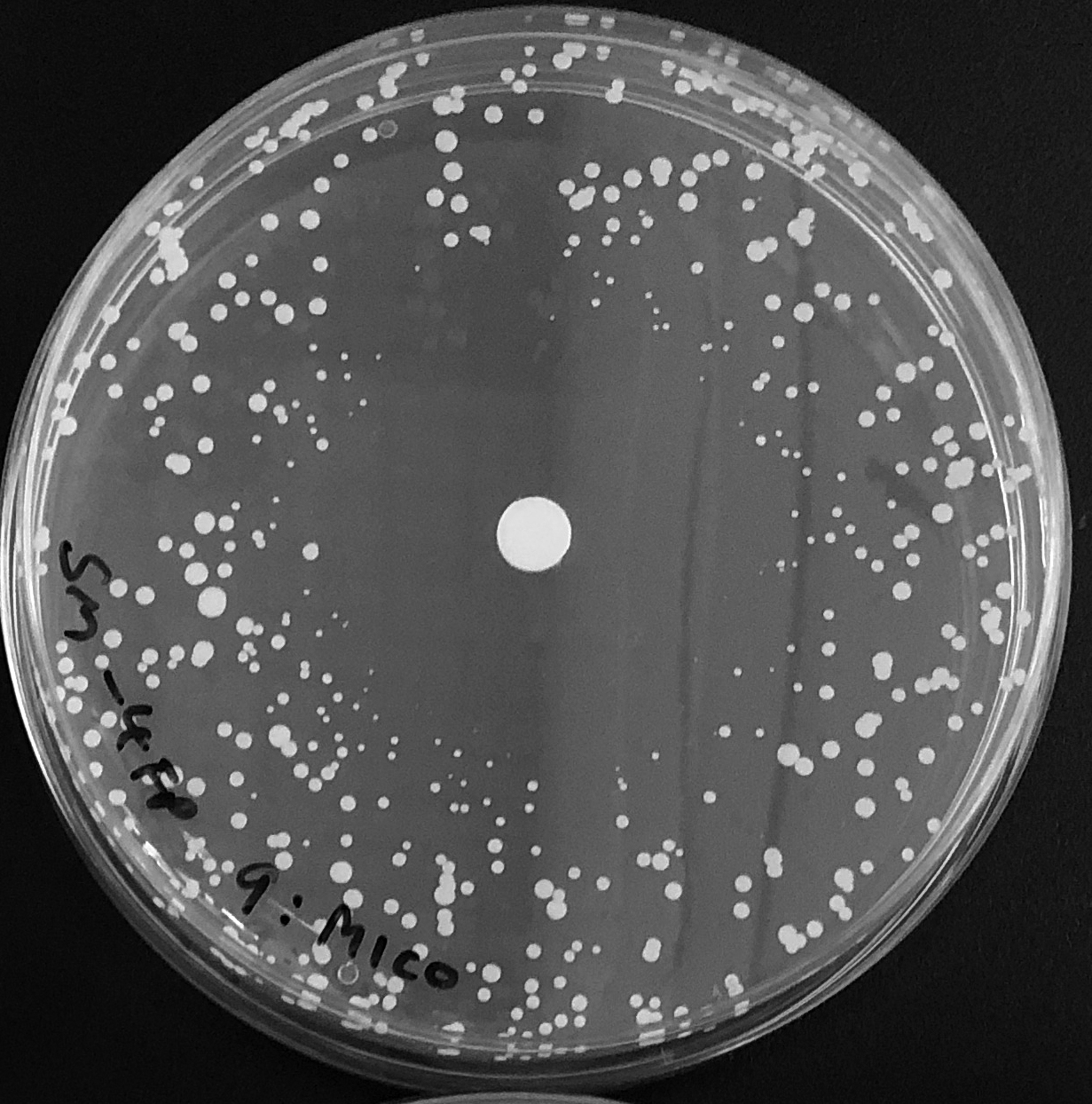

Supplement: Source Data Fig. 2 — Unprocessed images for Supplementary Fig. 2a,b. [file 41564_2022_1072_MOESM7_ESM.zip › MICO_4FP_SM.jpg]

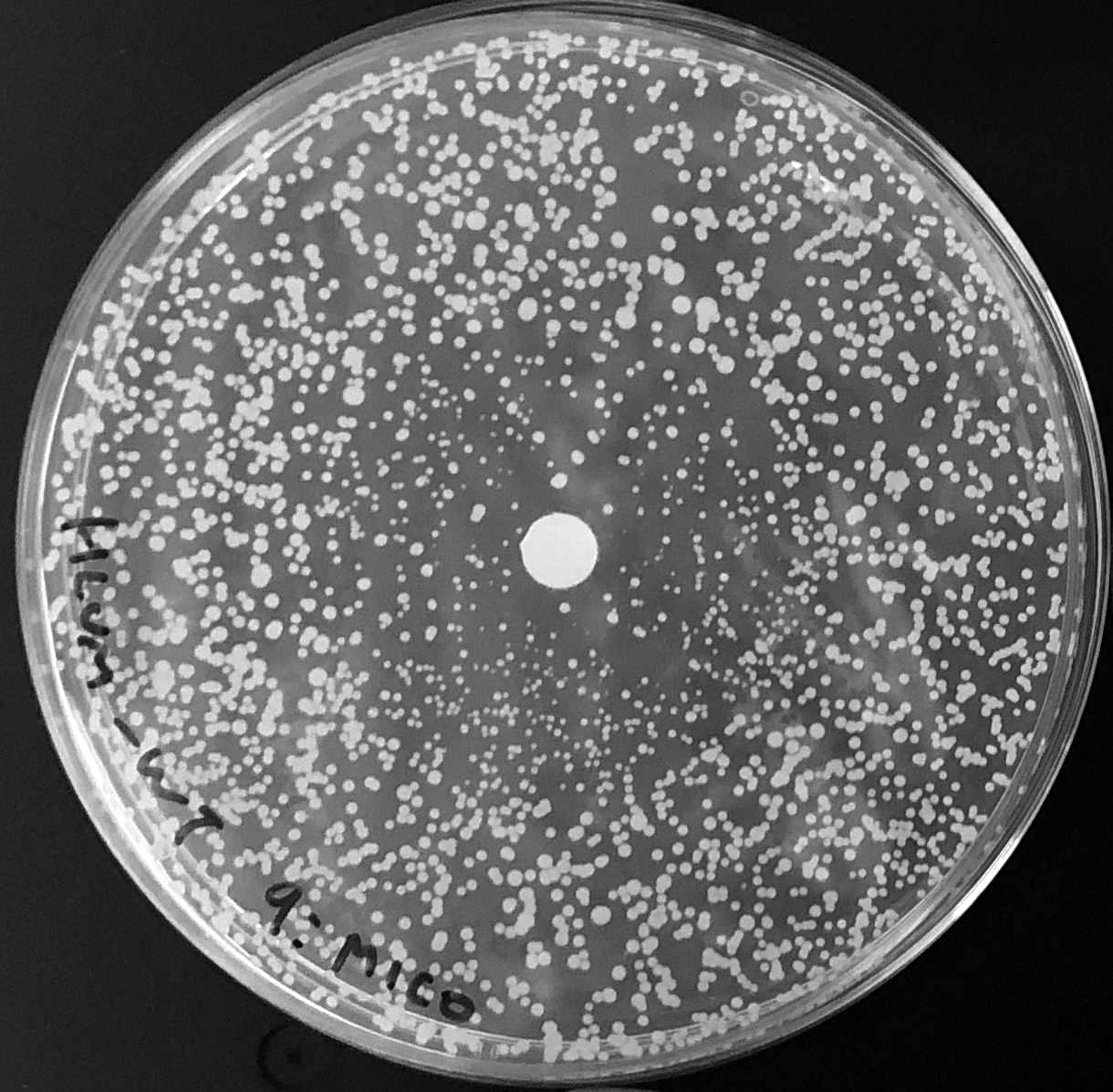

Supplement: Source Data Fig. 2 — Unprocessed images for Supplementary Fig. 2a,b. [file 41564_2022_1072_MOESM7_ESM.zip › MICO_WT_HLUM.jpg]

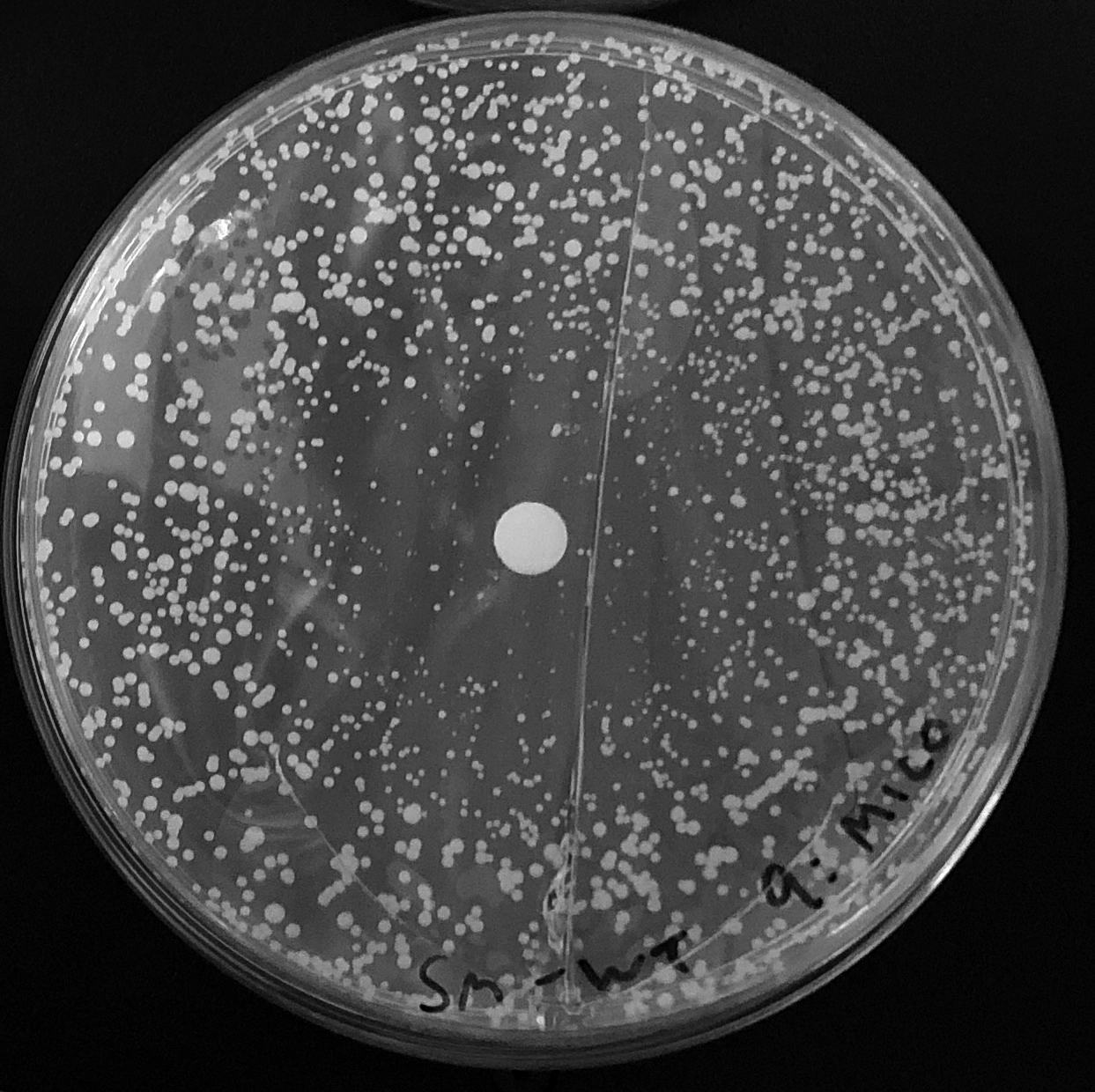

Supplement: Source Data Fig. 2 — Unprocessed images for Supplementary Fig. 2a,b. [file 41564_2022_1072_MOESM7_ESM.zip › MICO_WT_SM.jpg]

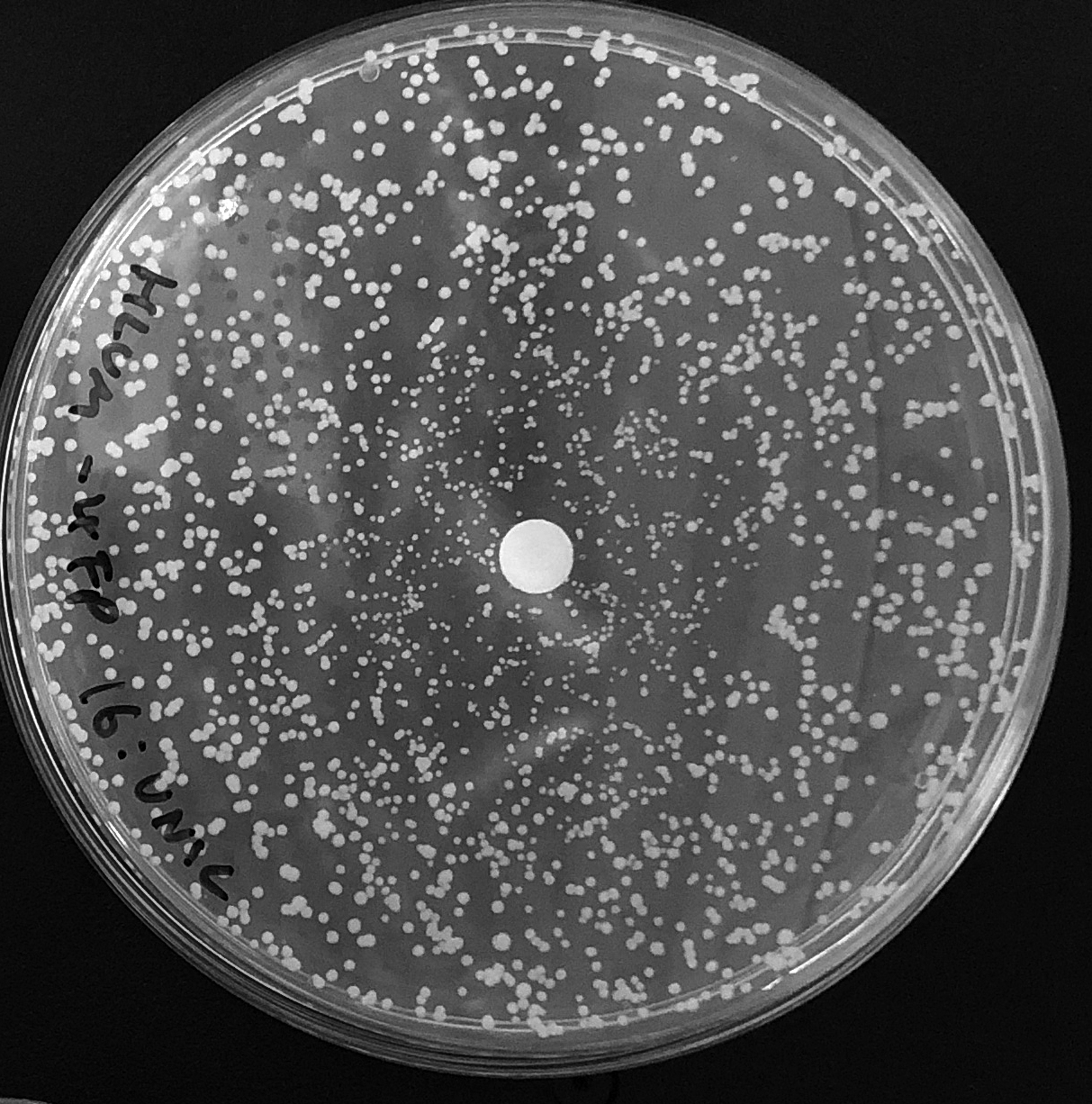

Supplement: Source Data Fig. 2 — Unprocessed images for Supplementary Fig. 2a,b. [file 41564_2022_1072_MOESM7_ESM.zip › UNIC_4FP_HLUM.jpg]

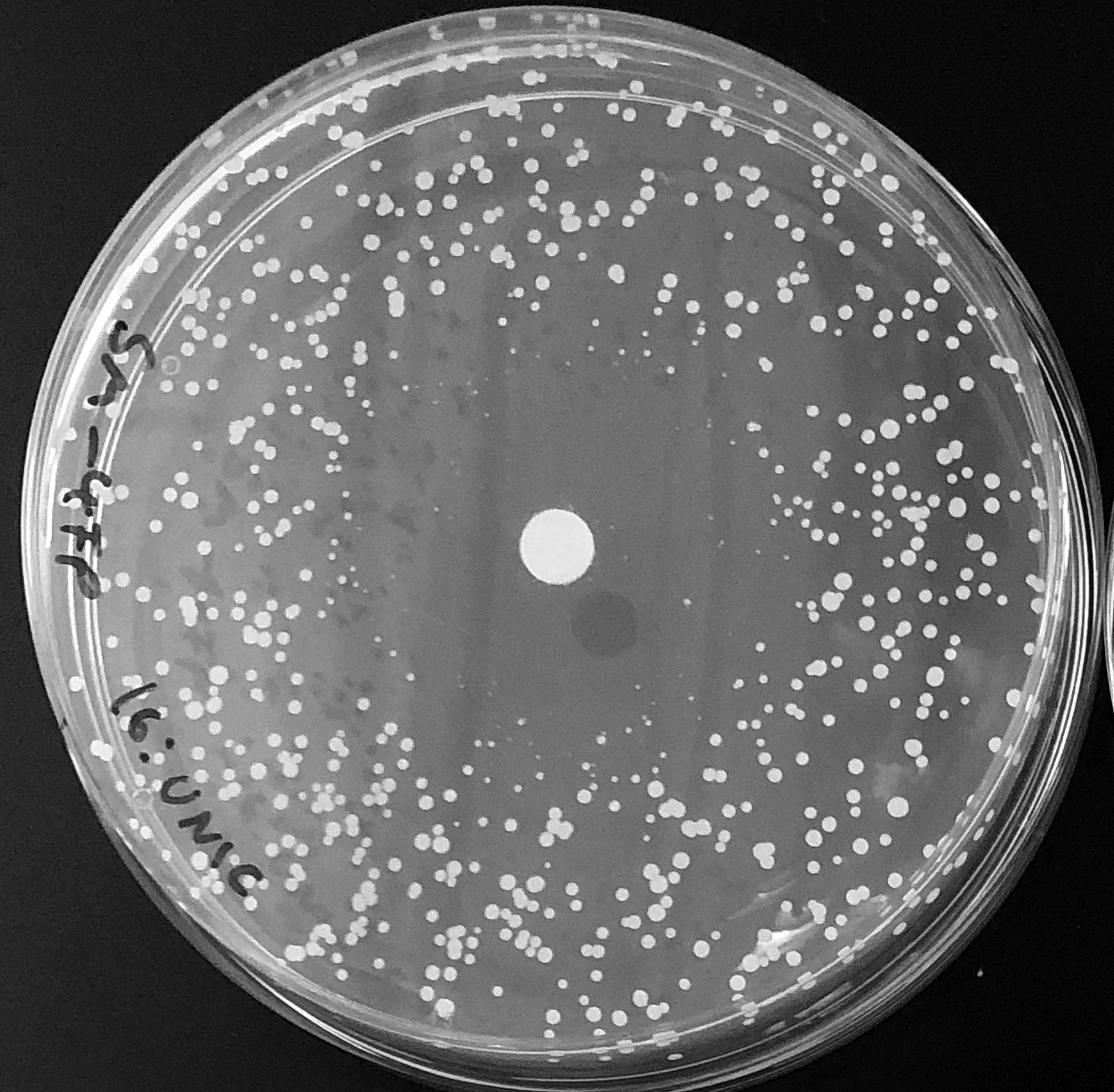

Supplement: Source Data Fig. 2 — Unprocessed images for Supplementary Fig. 2a,b. [file 41564_2022_1072_MOESM7_ESM.zip › UNIC_4FP_SM.jpg]

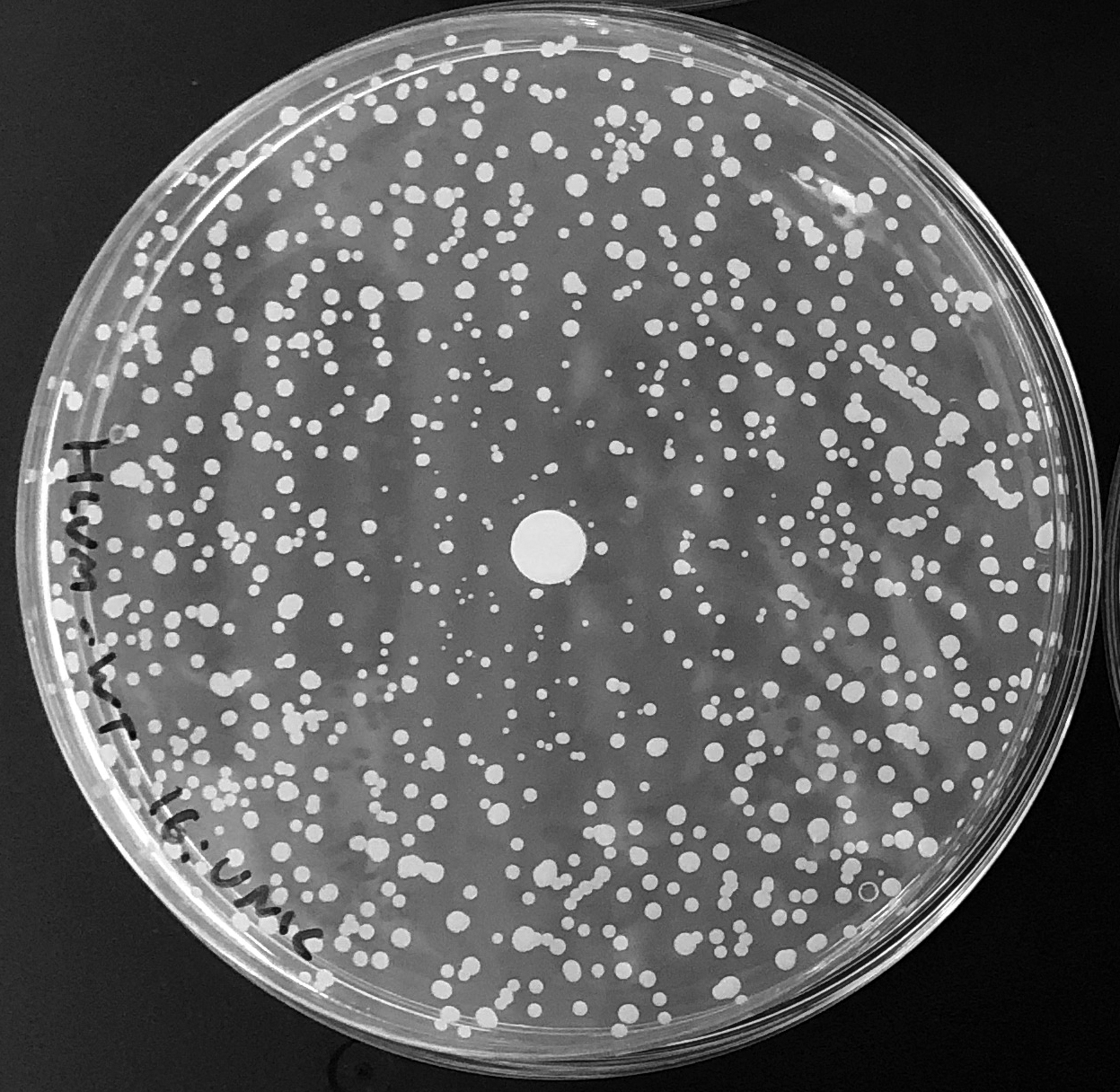

Supplement: Source Data Fig. 2 — Unprocessed images for Supplementary Fig. 2a,b. [file 41564_2022_1072_MOESM7_ESM.zip › UNIC_WT_HLUM.jpg]

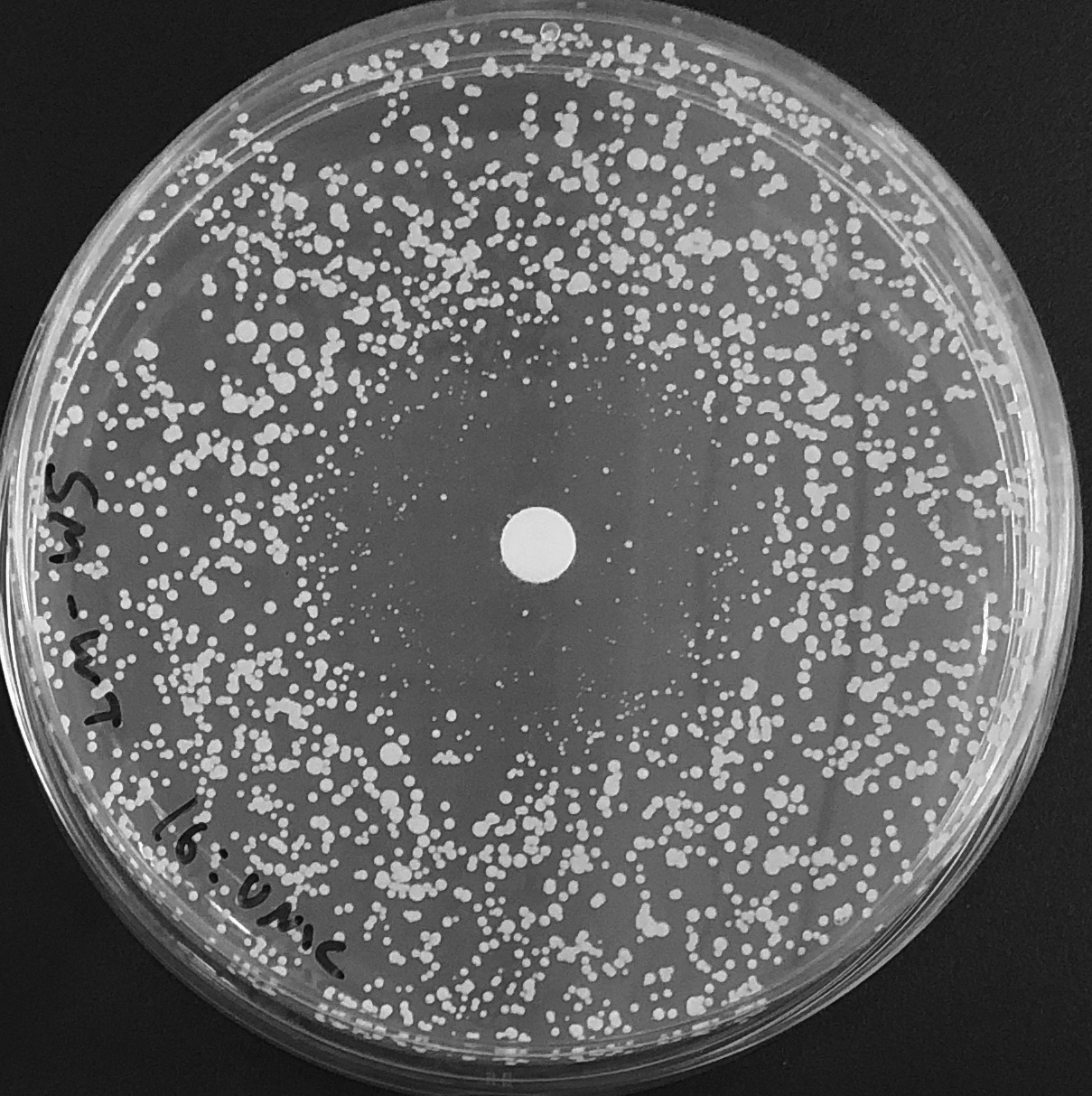

Supplement: Source Data Fig. 2 — Unprocessed images for Supplementary Fig. 2a,b. [file 41564_2022_1072_MOESM7_ESM.zip › UNIC_WT_SM.jpg]

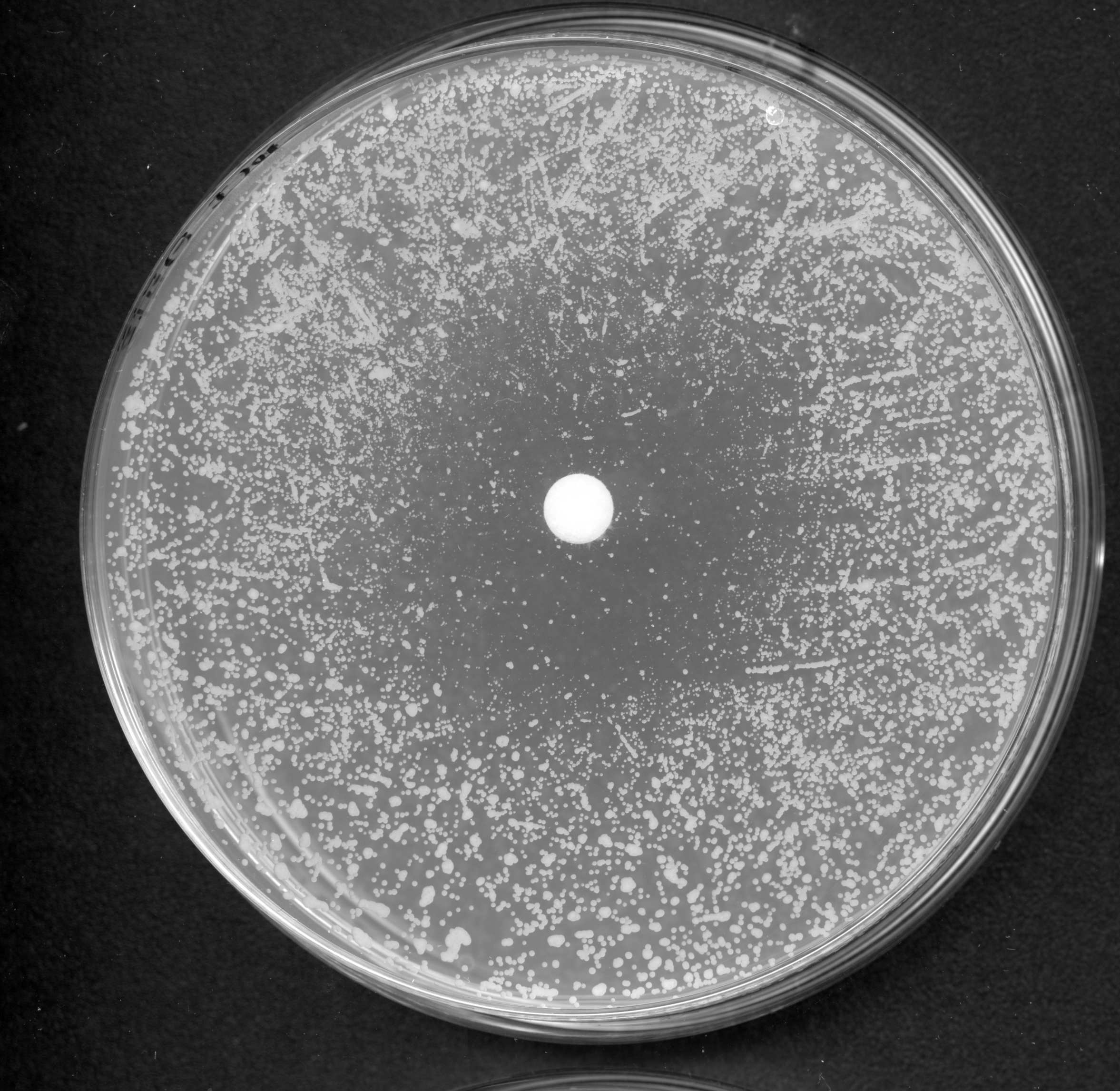

Supplement: Source Data Fig. 4 — Unprocessed images for Fig. 4e. [file 41564_2022_1072_MOESM10_ESM.zip › Source Data 4/UNIC_M+_HLUM.jpg]

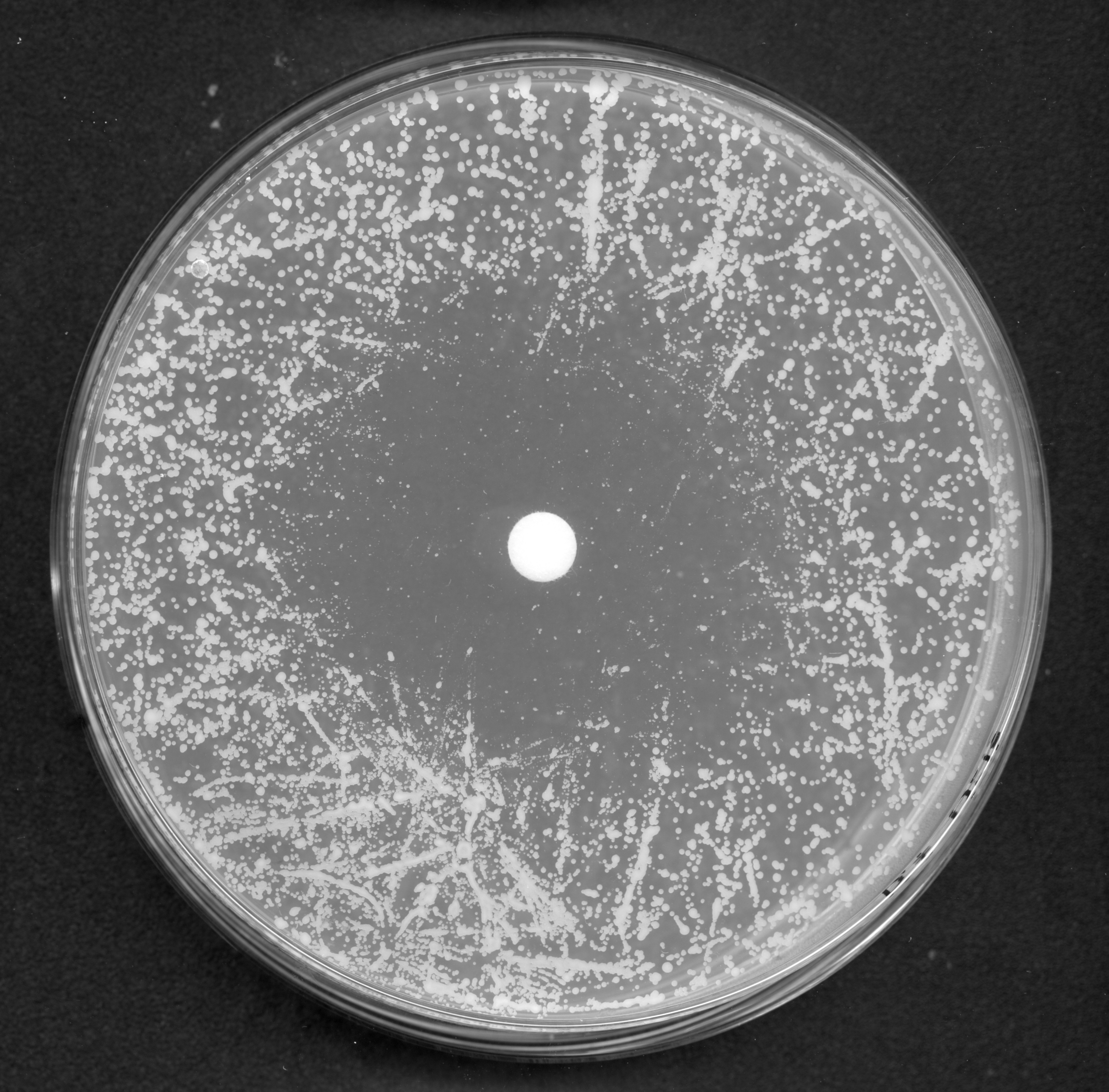

Supplement: Source Data Fig. 4 — Unprocessed images for Fig. 4e. [file 41564_2022_1072_MOESM10_ESM.zip › Source Data 4/UNIC_U+_HLUM.jpg]

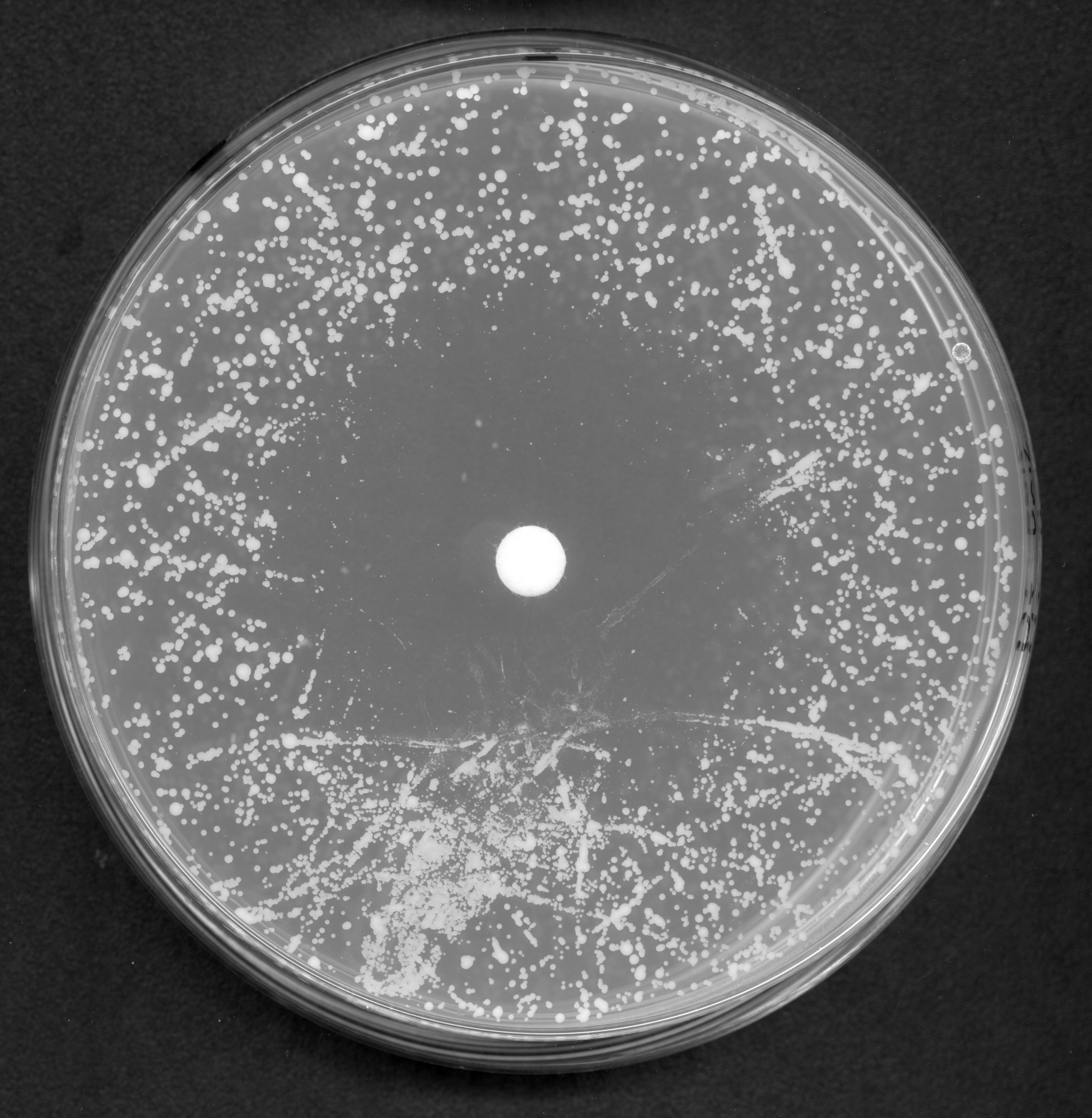

Supplement: Source Data Fig. 4 — Unprocessed images for Fig. 4e. [file 41564_2022_1072_MOESM10_ESM.zip › Source Data 4/UNIC_U+_SM.jpg]

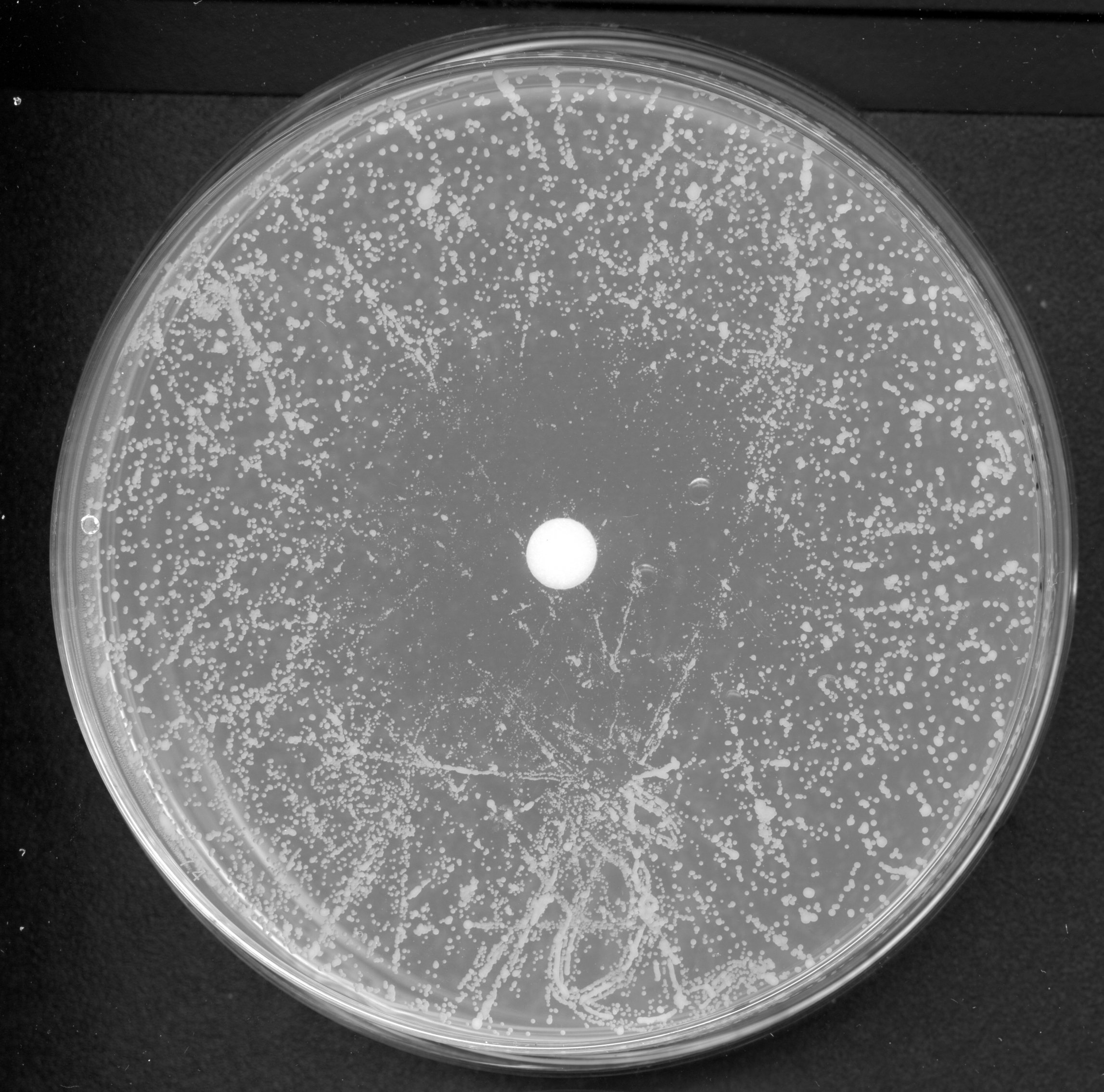

Supplement: Source Data Fig. 4 — Unprocessed images for Fig. 4e. [file 41564_2022_1072_MOESM10_ESM.zip › Source Data 4/UNIC_L-_HLUM.jpg]

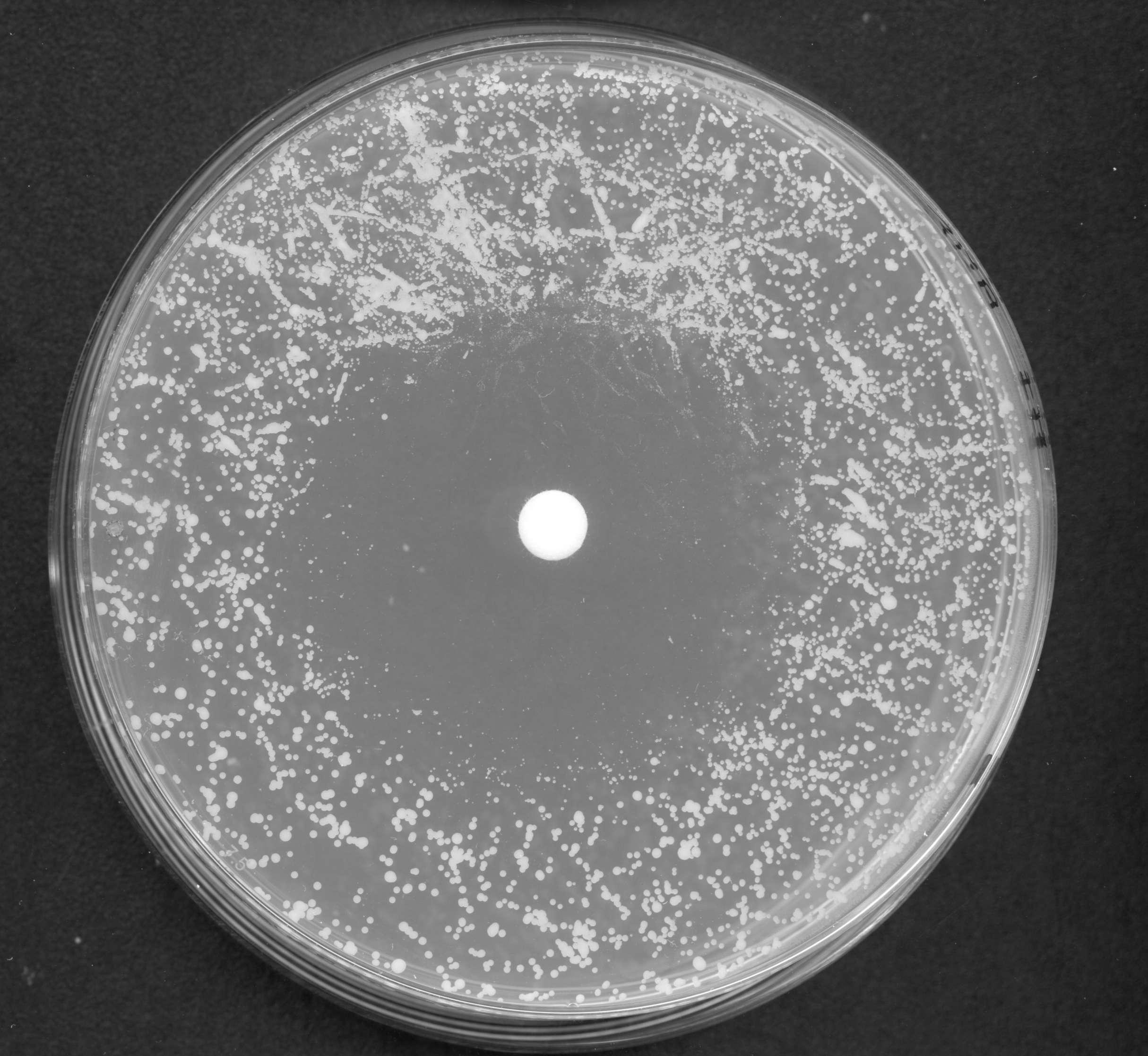

Supplement: Source Data Fig. 4 — Unprocessed images for Fig. 4e. [file 41564_2022_1072_MOESM10_ESM.zip › Source Data 4/UNIC_H+_SM.jpg]

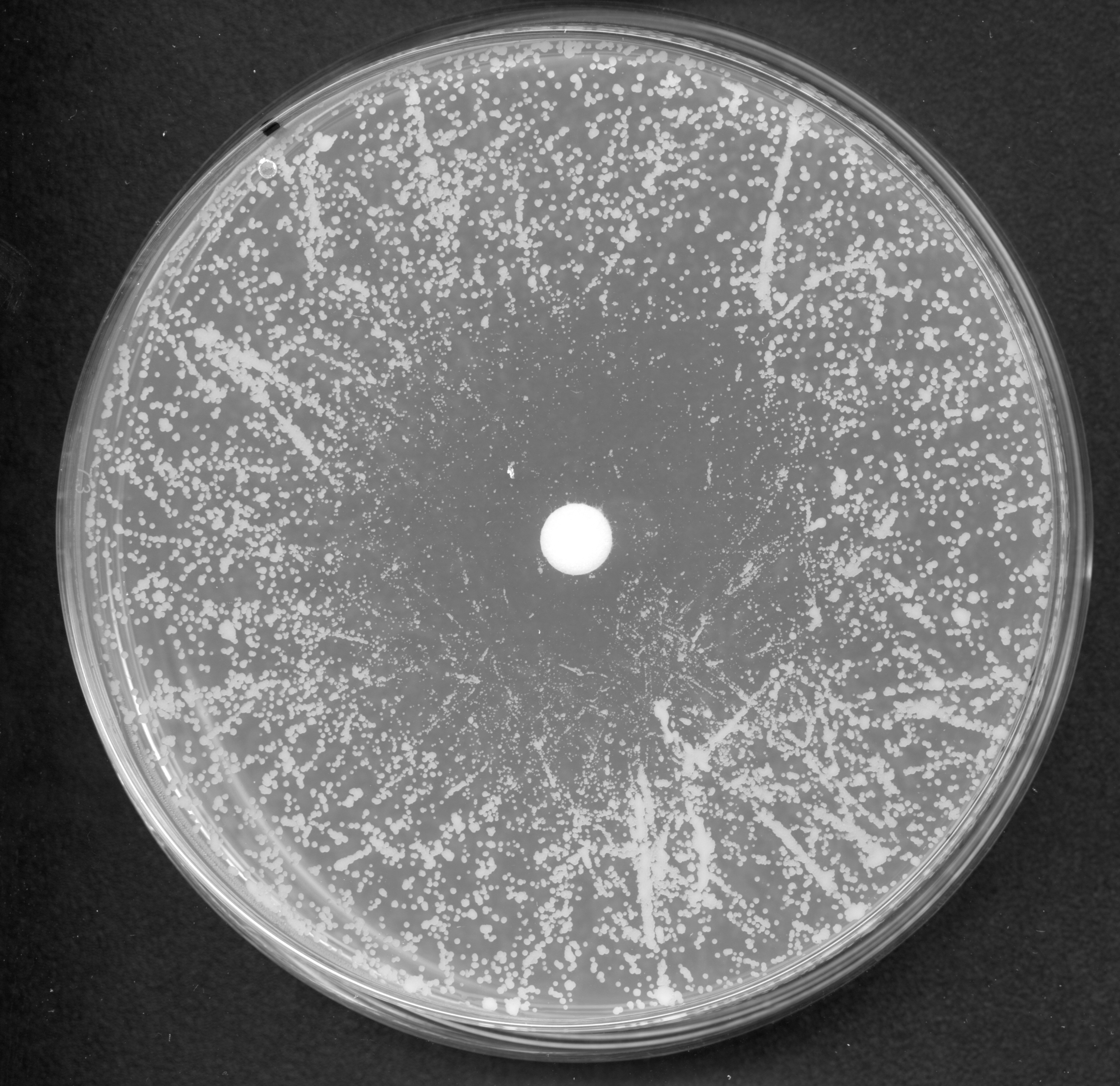

Supplement: Source Data Fig. 4 — Unprocessed images for Fig. 4e. [file 41564_2022_1072_MOESM10_ESM.zip › Source Data 4/UNIC_L+_HLUM.jpg]

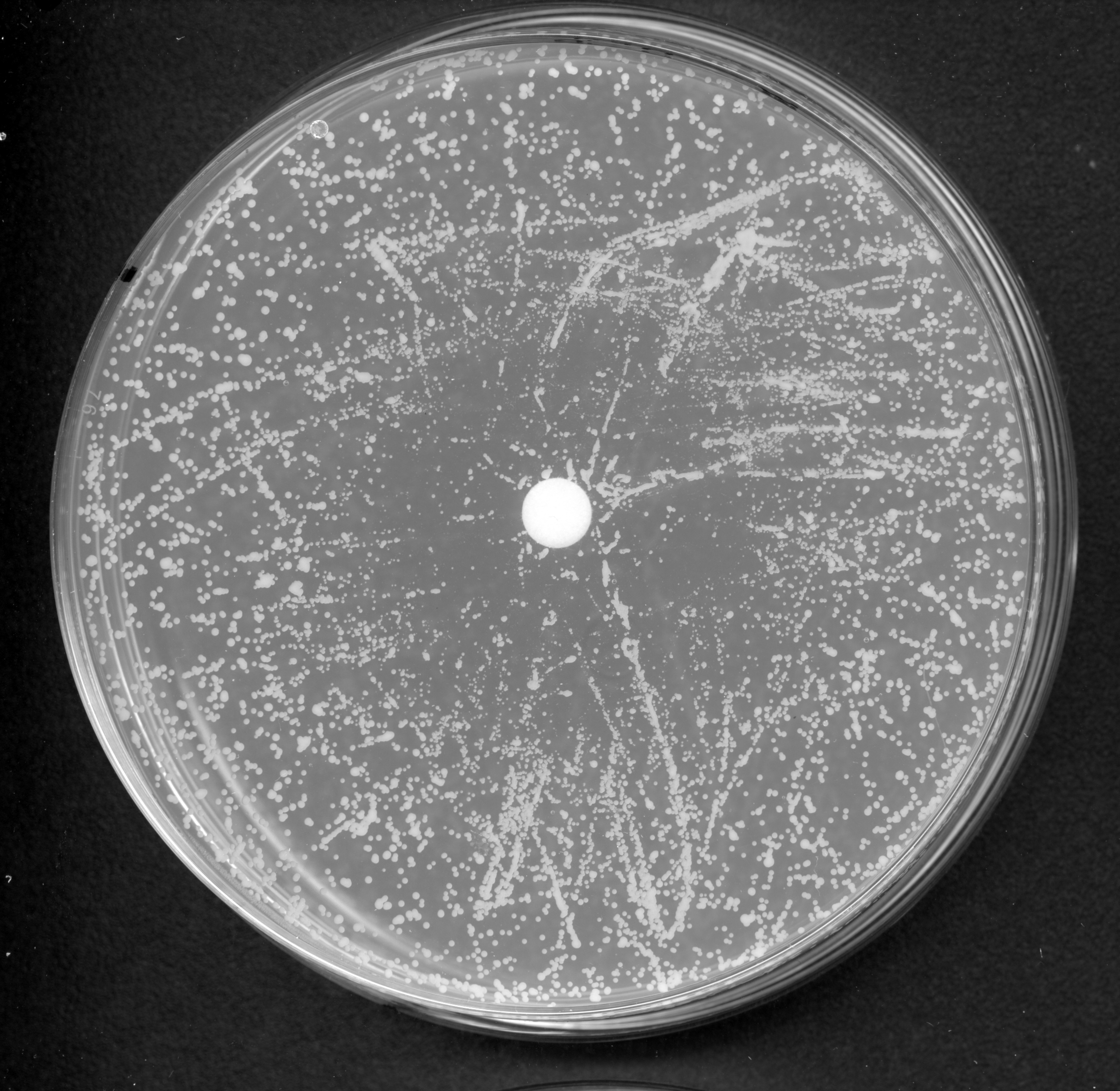

Supplement: Source Data Fig. 4 — Unprocessed images for Fig. 4e. [file 41564_2022_1072_MOESM10_ESM.zip › Source Data 4/UNIC_M-_HLUM.jpg]

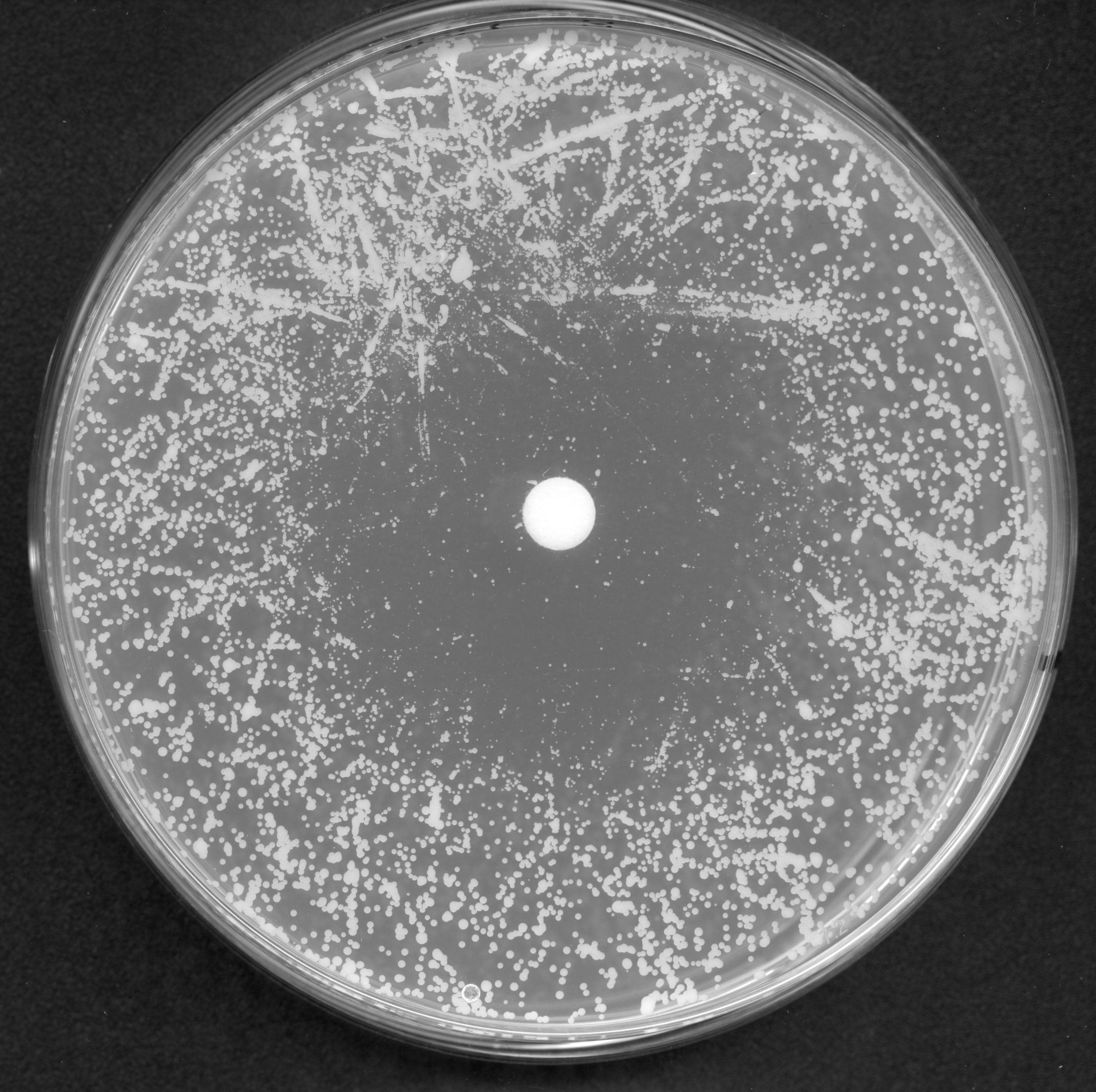

Supplement: Source Data Fig. 4 — Unprocessed images for Fig. 4e. [file 41564_2022_1072_MOESM10_ESM.zip › Source Data 4/UNIC_U-_HLUM.jpg]

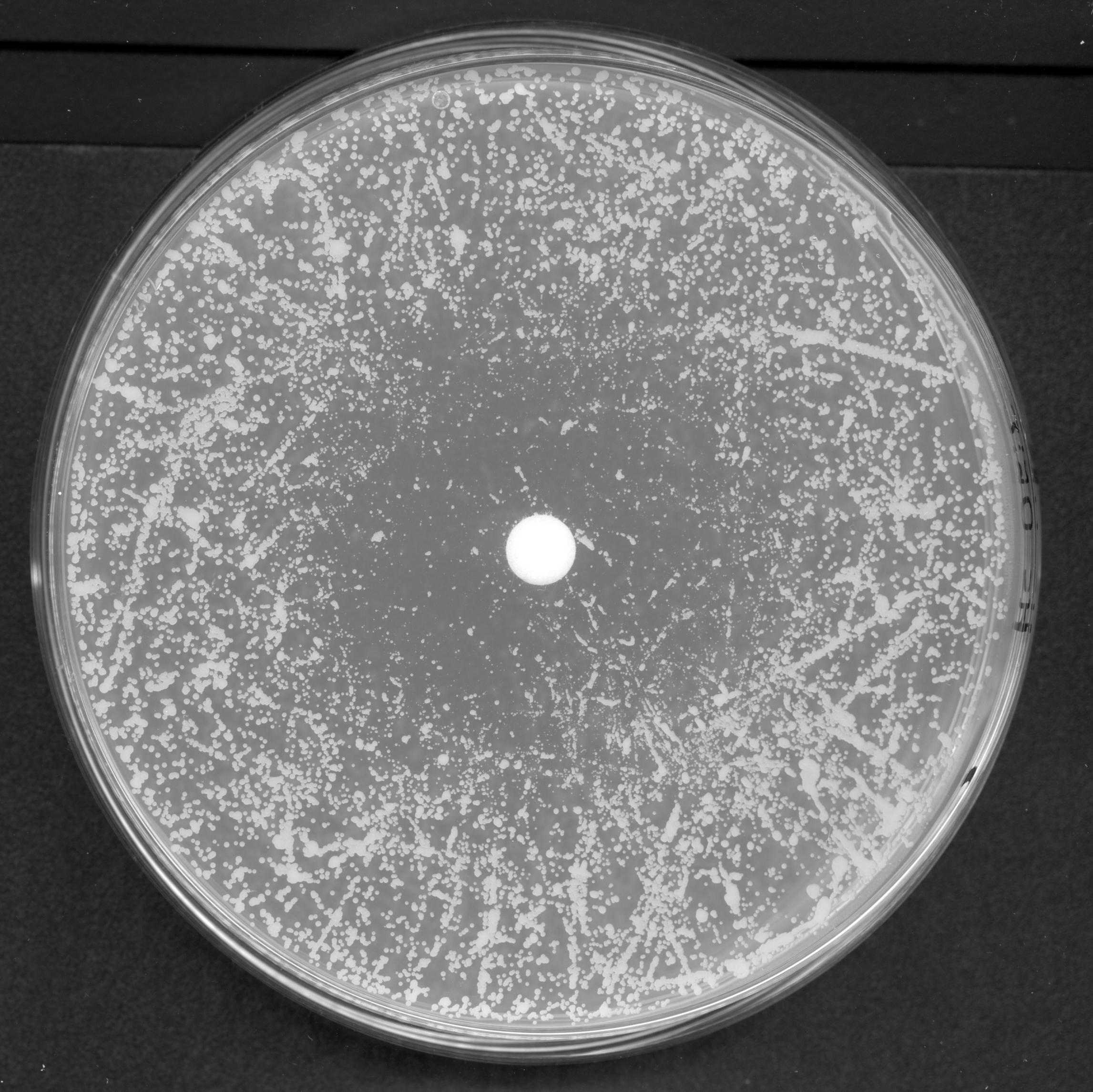

Supplement: Source Data Fig. 4 — Unprocessed images for Fig. 4e. [file 41564_2022_1072_MOESM10_ESM.zip › Source Data 4/UNIC_H-_HLUM.jpg]

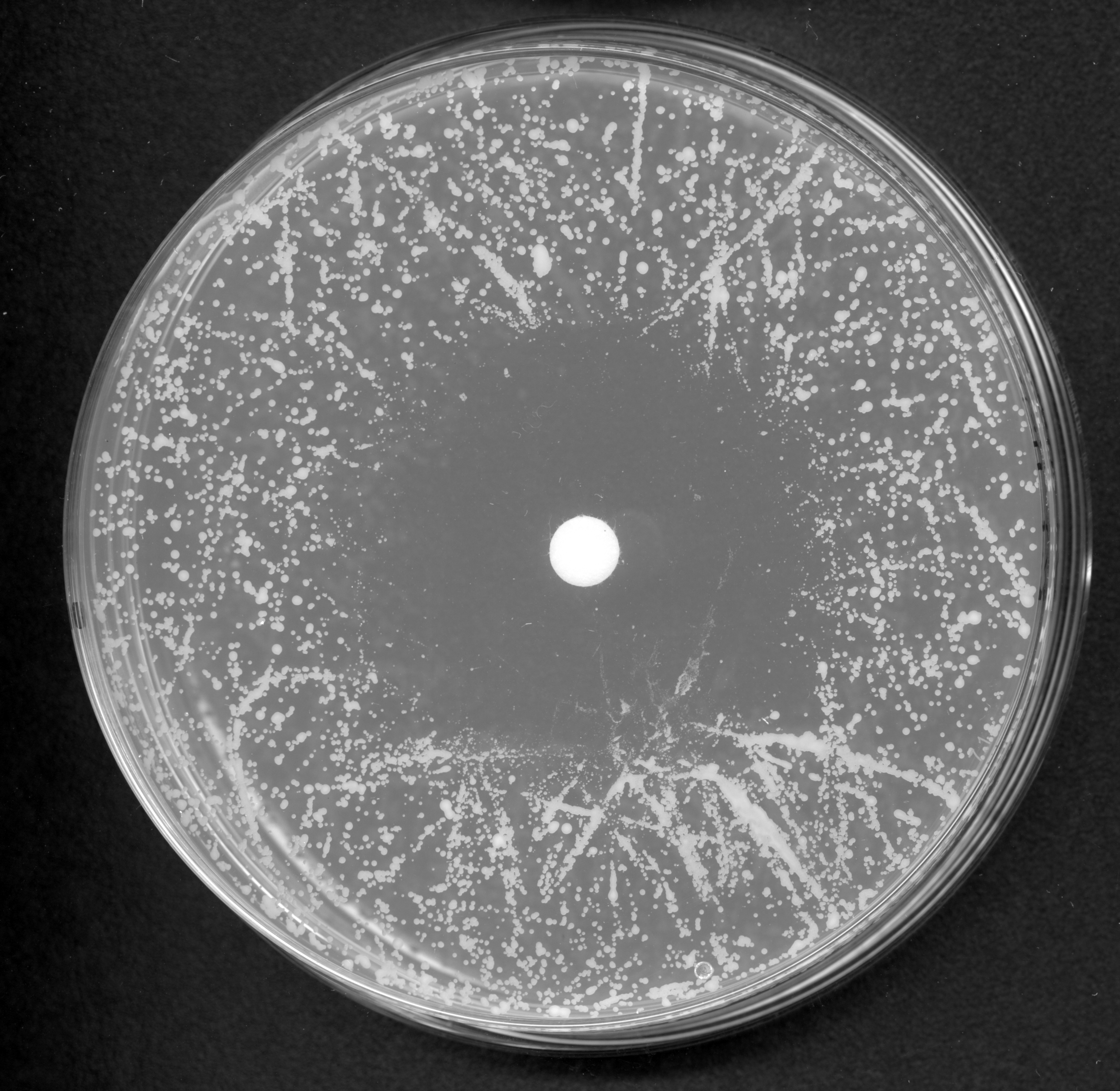

Supplement: Source Data Fig. 4 — Unprocessed images for Fig. 4e. [file 41564_2022_1072_MOESM10_ESM.zip › Source Data 4/UNIC_M+_SM.jpg]

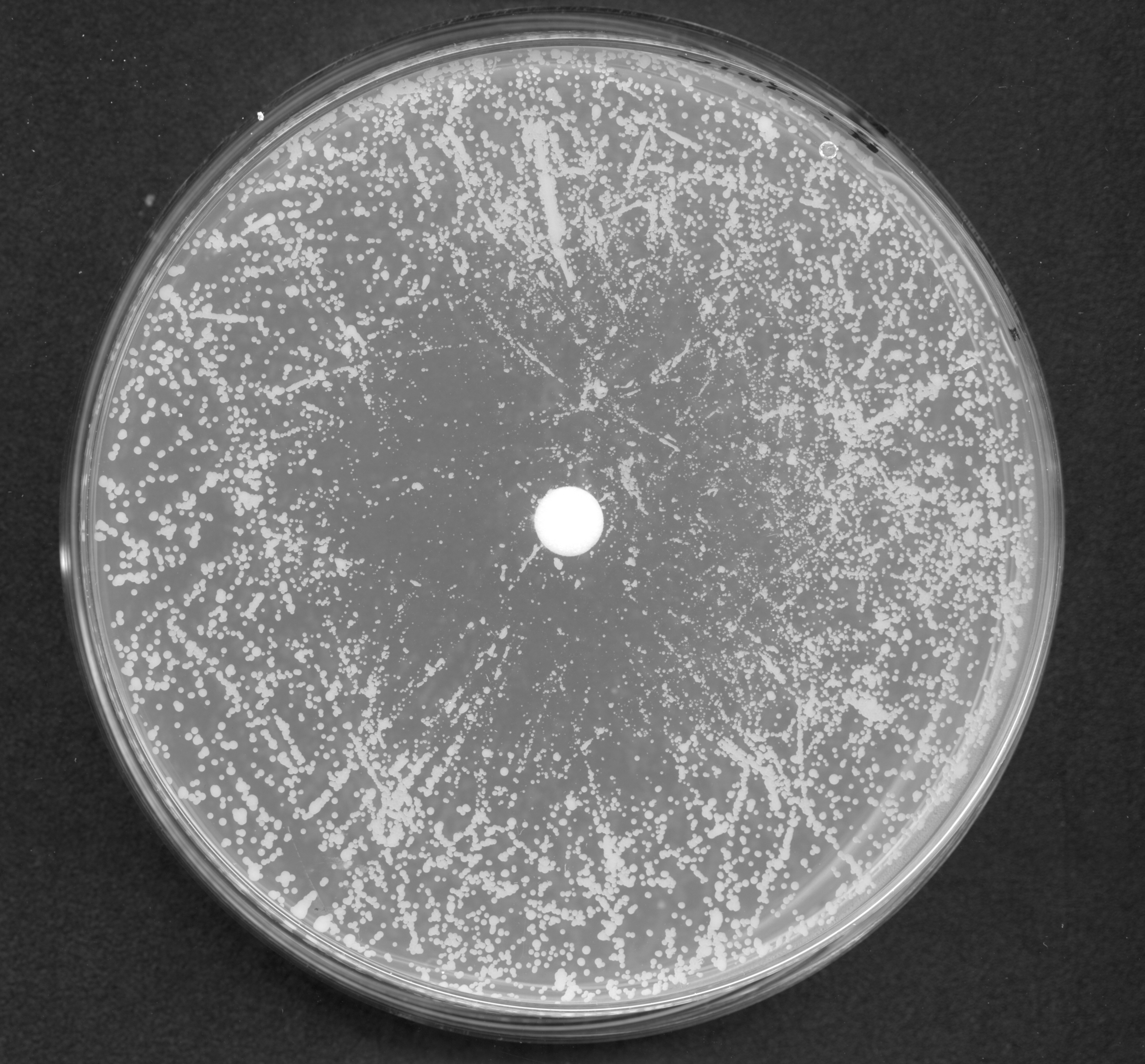

Supplement: Source Data Fig. 4 — Unprocessed images for Fig. 4e. [file 41564_2022_1072_MOESM10_ESM.zip › Source Data 4/UNIC_H+_HLUM.jpg]

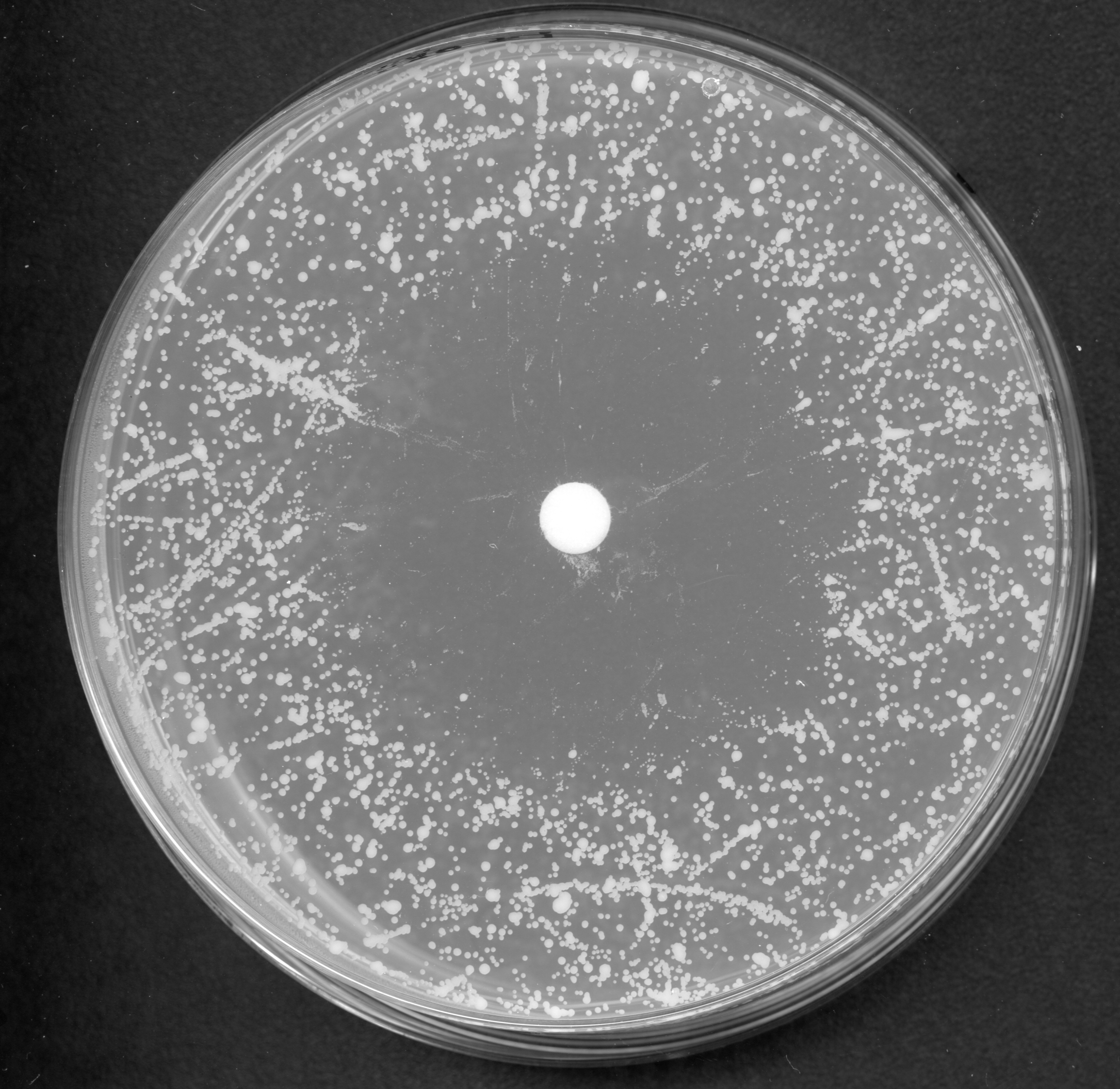

Supplement: Source Data Fig. 4 — Unprocessed images for Fig. 4e. [file 41564_2022_1072_MOESM10_ESM.zip › Source Data 4/UNIC_L+_SM.jpg]

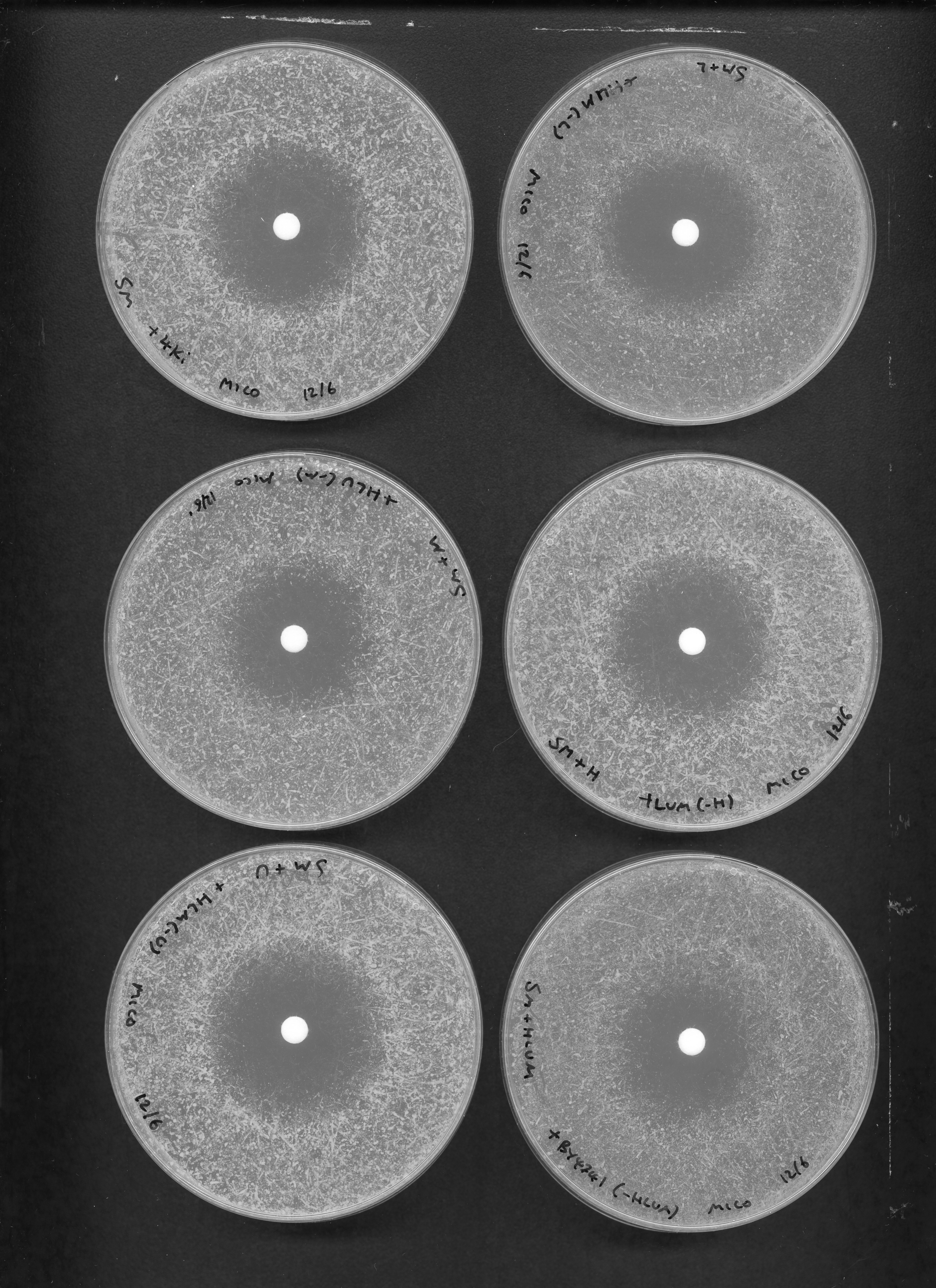

Supplement: Source Data Extended Data Fig. 2. — Unprocessed images for Supplementary Fig. 2a,b. [file 41564_2022_1072_MOESM12_ESM.zip › Source Data S2b/img20210614_07204383.jpg]

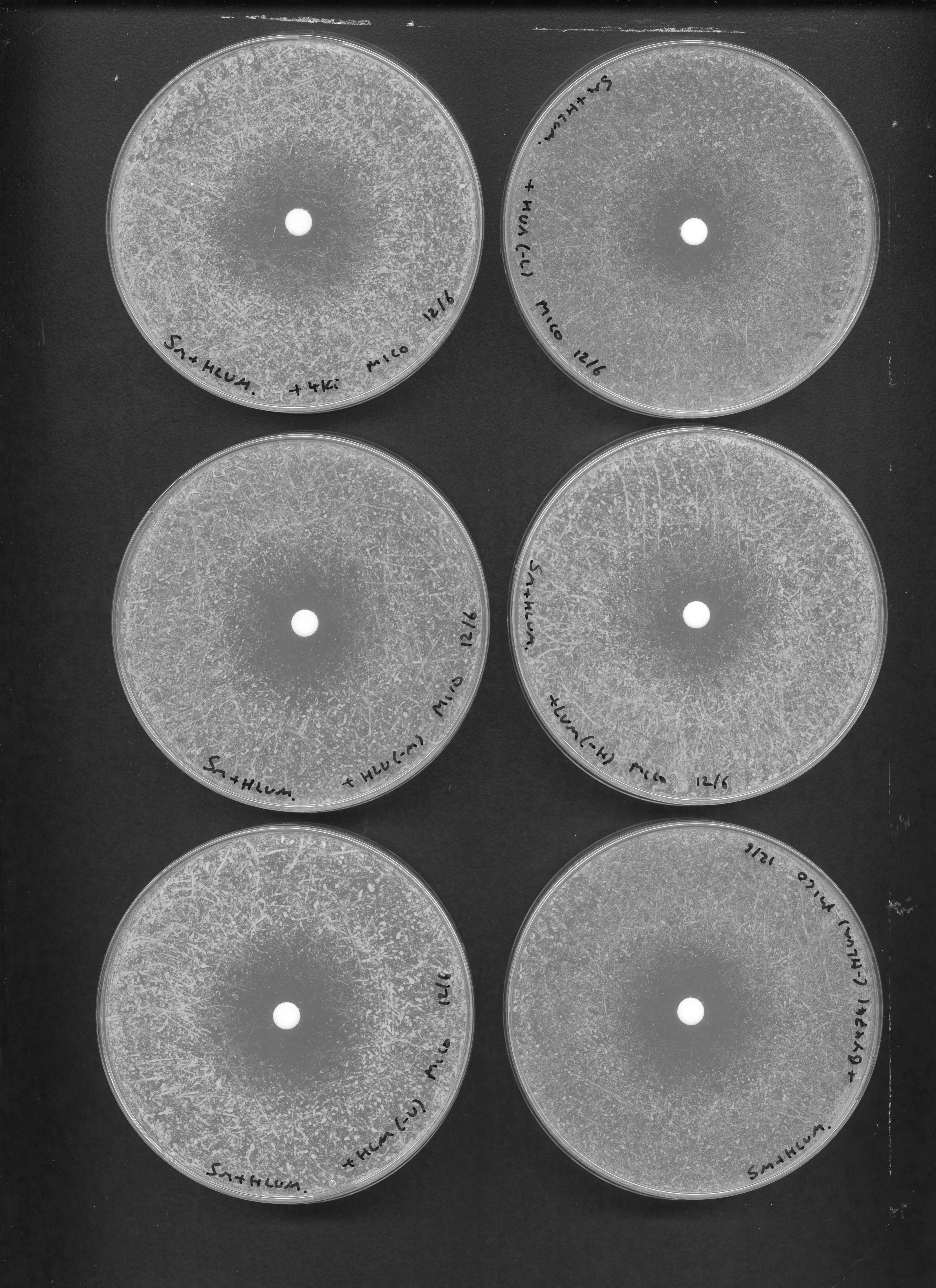

Supplement: Source Data Extended Data Fig. 2. — Unprocessed images for Supplementary Fig. 2a,b. [file 41564_2022_1072_MOESM12_ESM.zip › Source Data S2b/img20210614_07184264.jpg]

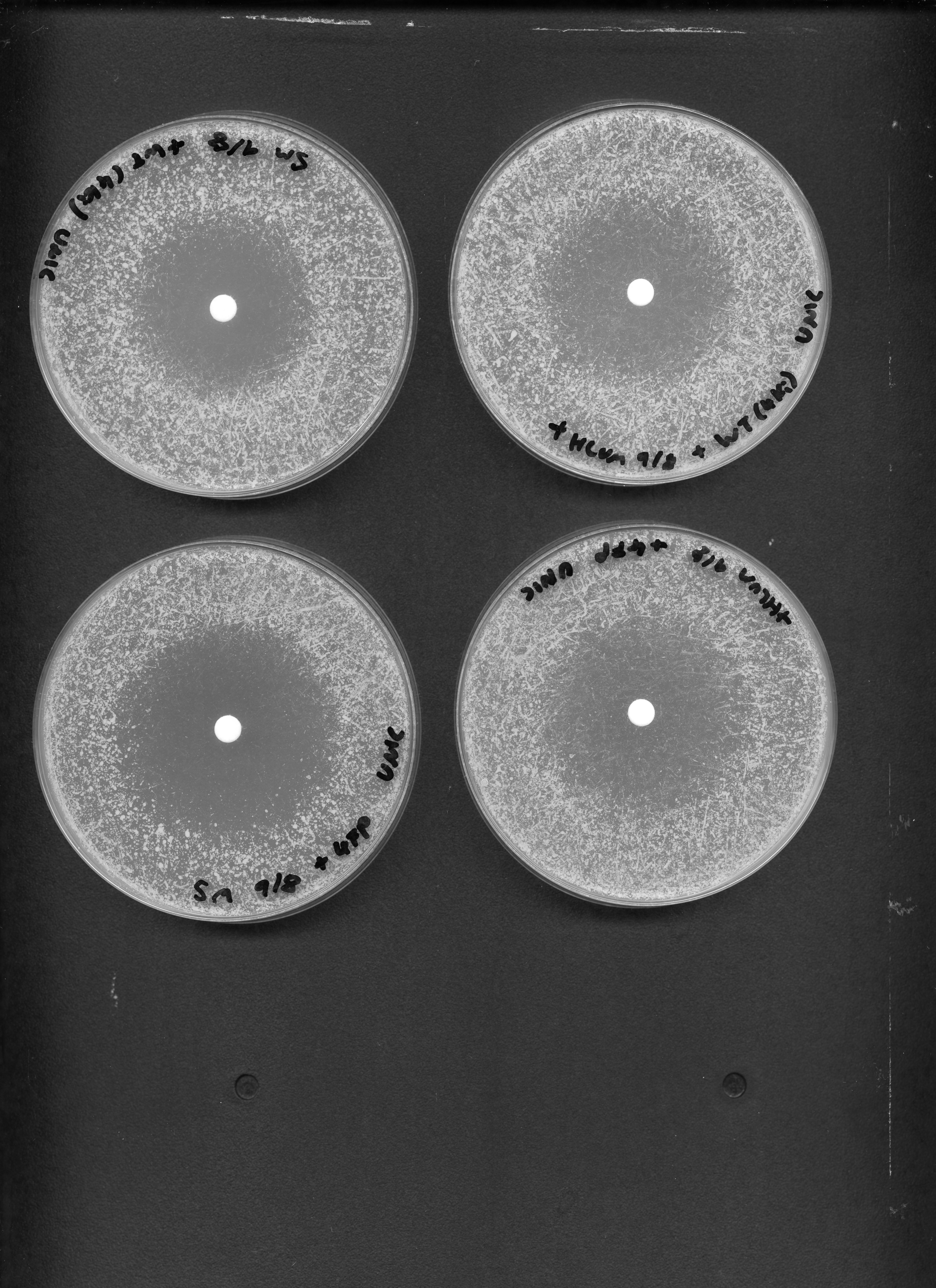

Supplement: Source Data Extended Data Fig. 2. — Unprocessed images for Supplementary Fig. 2a,b. [file 41564_2022_1072_MOESM12_ESM.zip › Source Data S2a/img20210813_09560297.jpg]

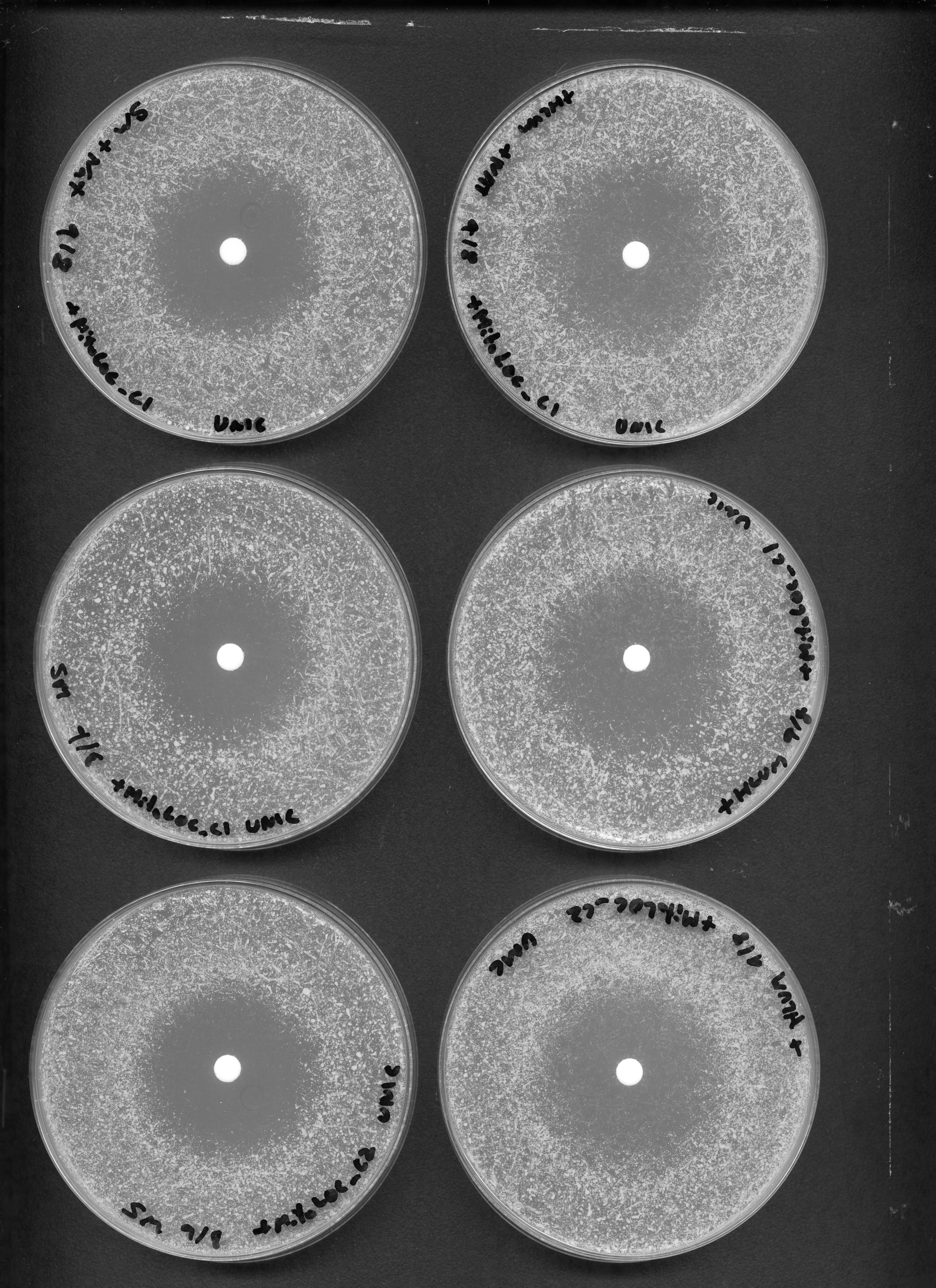

Supplement: Source Data Extended Data Fig. 2. — Unprocessed images for Supplementary Fig. 2a,b. [file 41564_2022_1072_MOESM12_ESM.zip › Source Data S2a/img20210813_09543338.jpg]

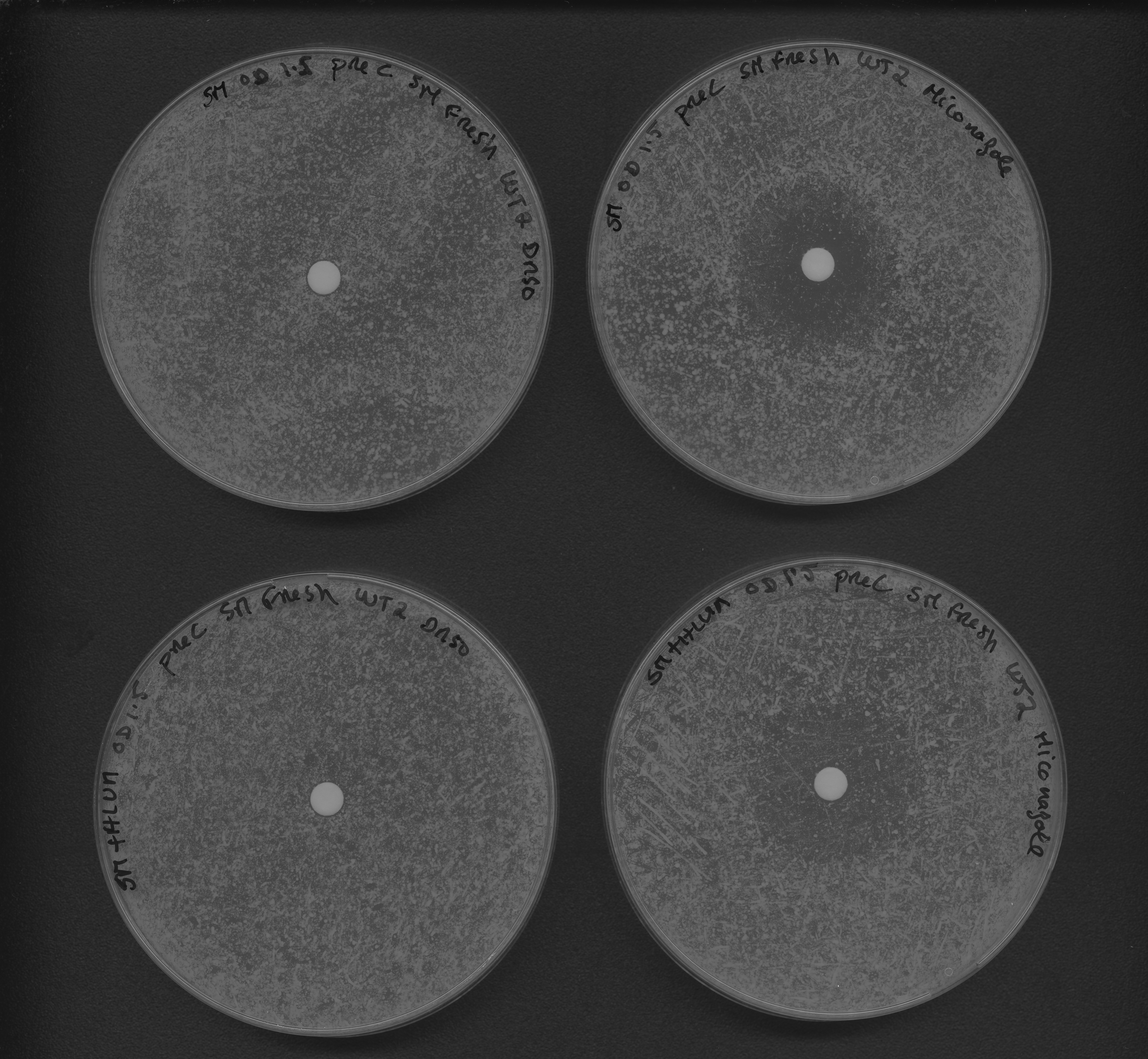

Supplement: Source Data Extended Data Fig. 5 — Unprocessed images for Supplementary Fig. 5a,b. [file 41564_2022_1072_MOESM15_ESM.zip › Source Data S5b/OD15_preC_SMfresh_wt2.jpg]

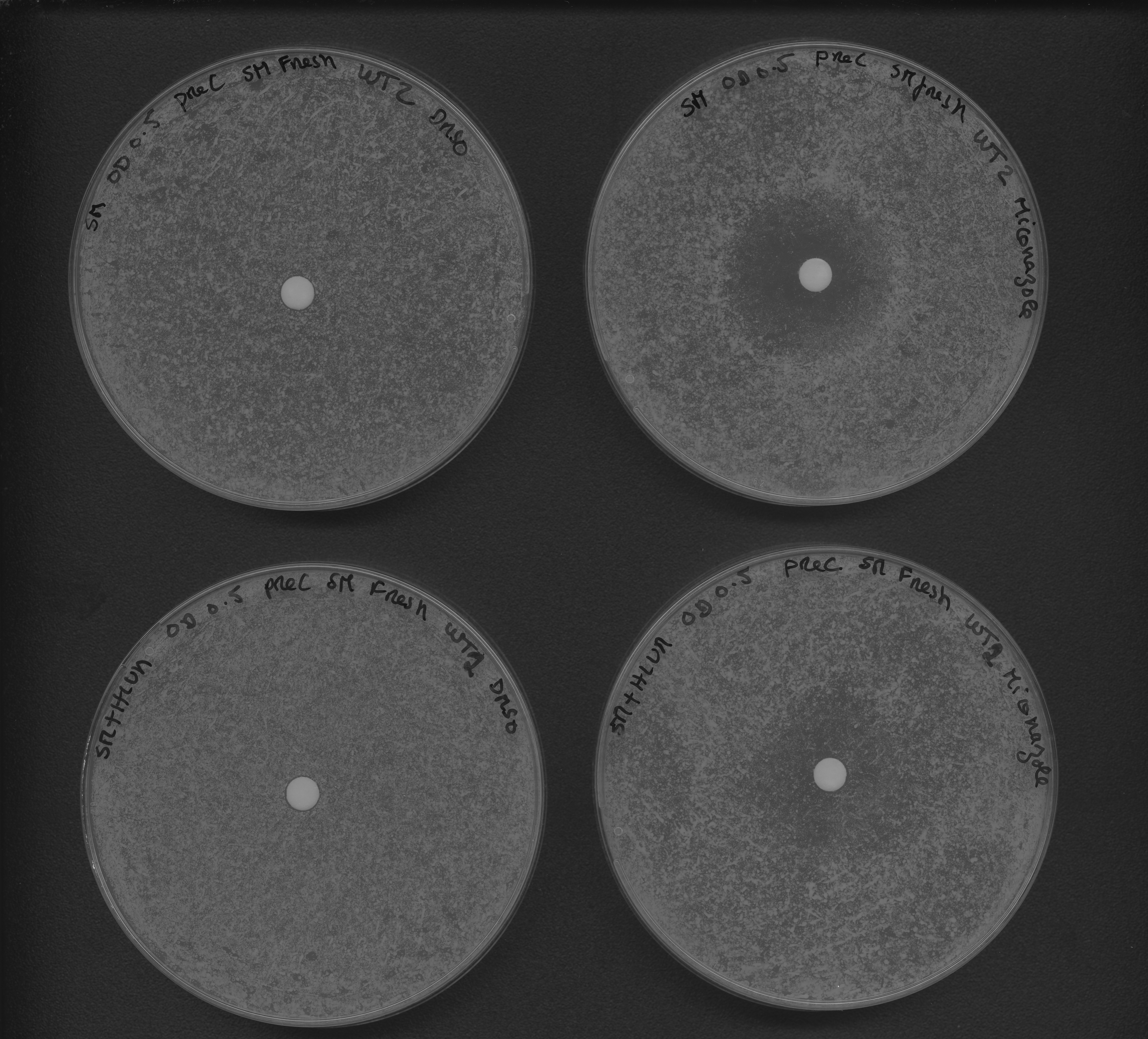

Supplement: Source Data Extended Data Fig. 5 — Unprocessed images for Supplementary Fig. 5a,b. [file 41564_2022_1072_MOESM15_ESM.zip › Source Data S5b/OD05_preC_SMfresh_wt2.jpg]

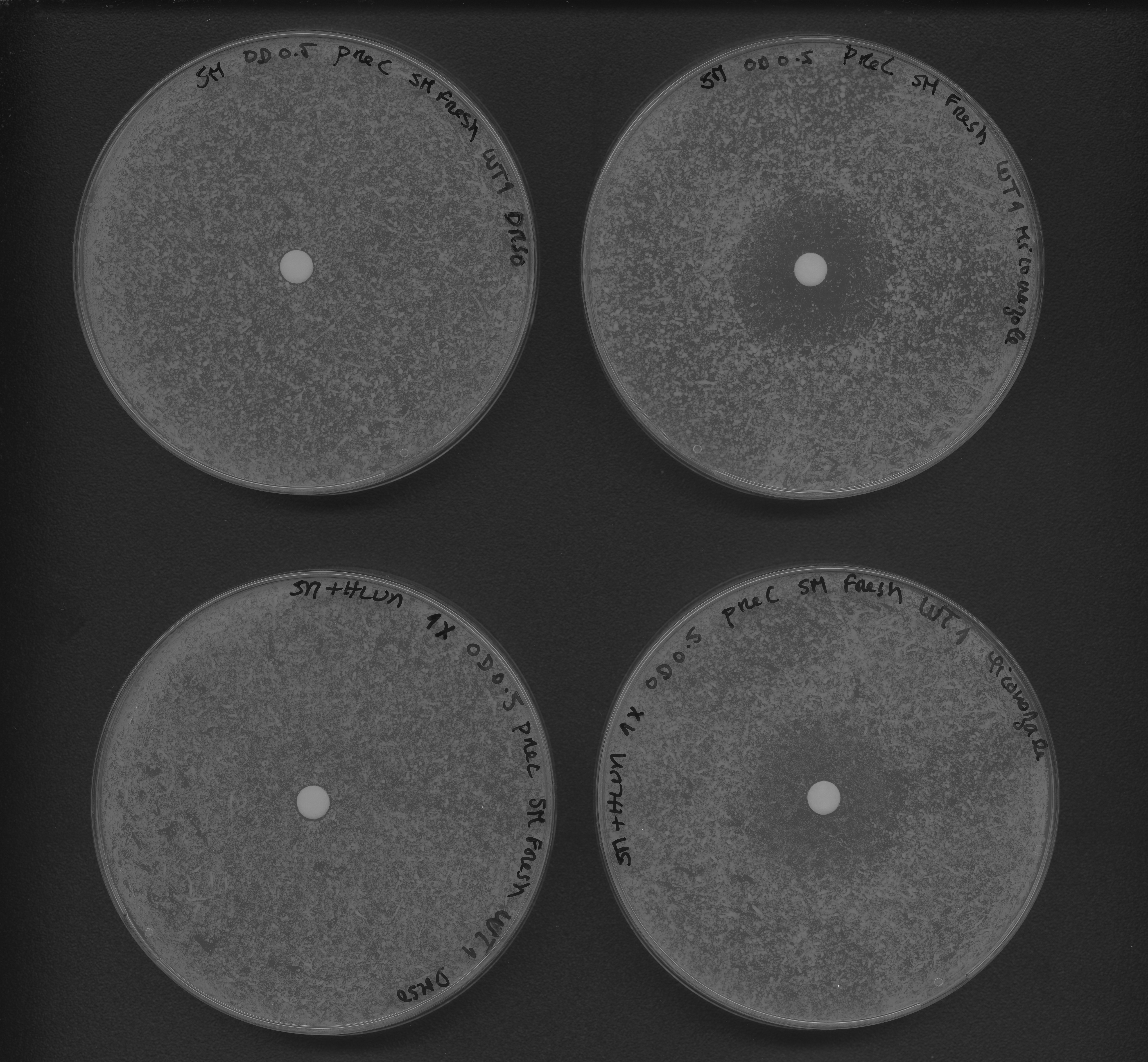

Supplement: Source Data Extended Data Fig. 5 — Unprocessed images for Supplementary Fig. 5a,b. [file 41564_2022_1072_MOESM15_ESM.zip › Source Data S5a/hlum_concentration_test007.jpg]

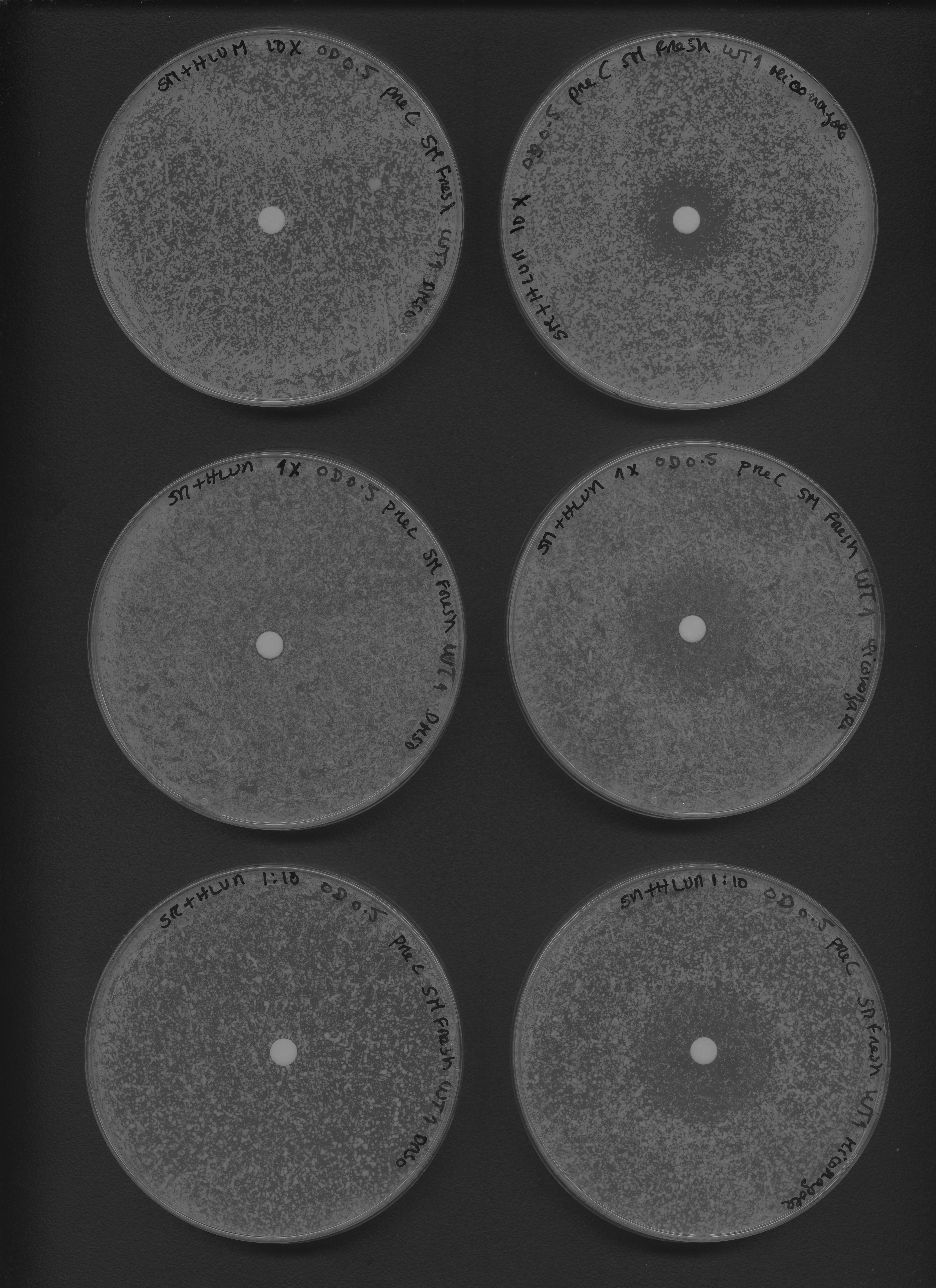

Supplement: Source Data Extended Data Fig. 5 — Unprocessed images for Supplementary Fig. 5a,b. [file 41564_2022_1072_MOESM15_ESM.zip › Source Data S5a/hlum_concentration_test005.jpg]

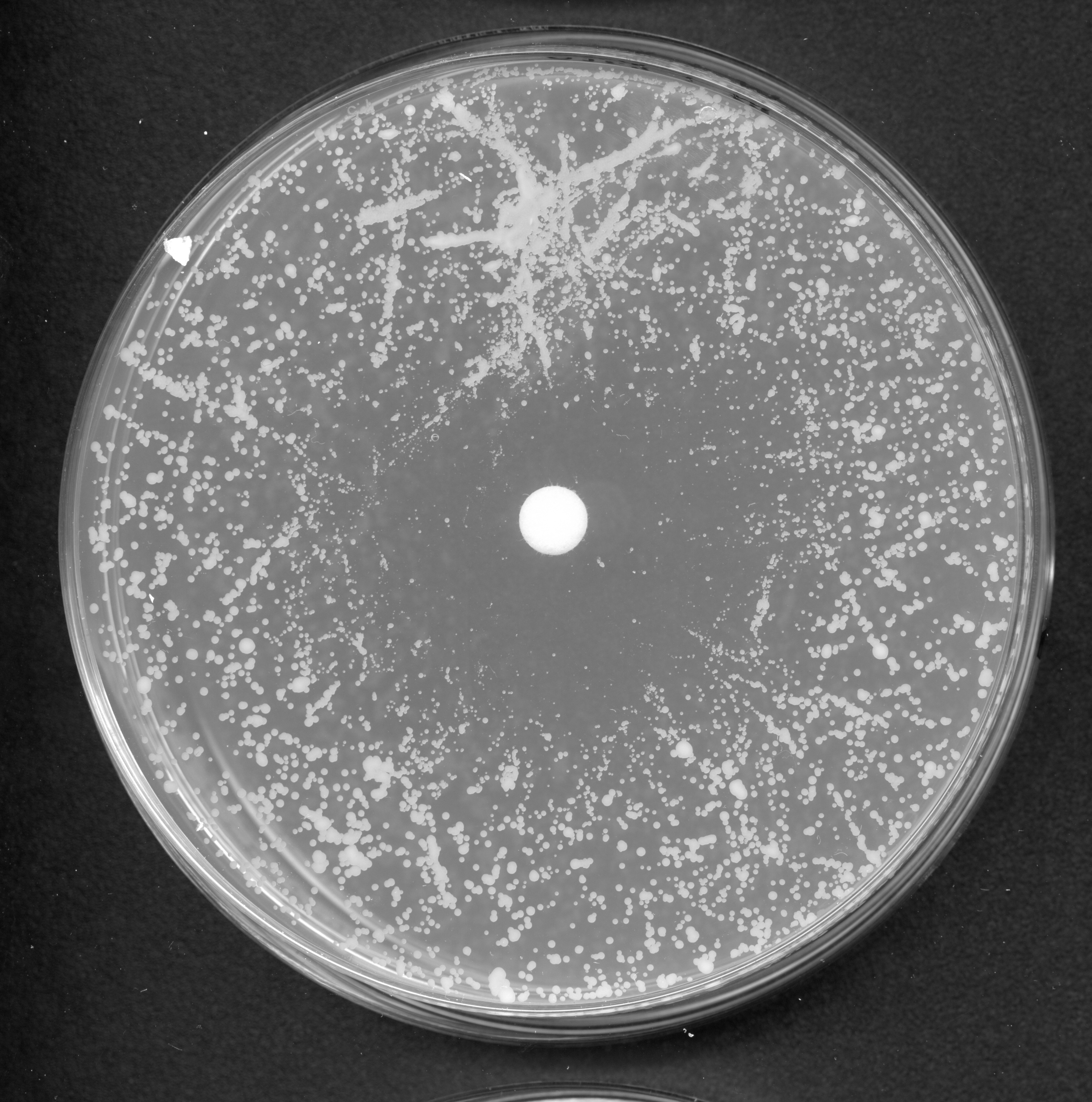

Supplement: Source Data Extended Data Fig. 10 — Unprocessed images for Supplementary Fig. 10d. [file 41564_2022_1072_MOESM21_ESM.zip › Source Data S10/Source Data S10d/MICO_M+_SM.jpg]

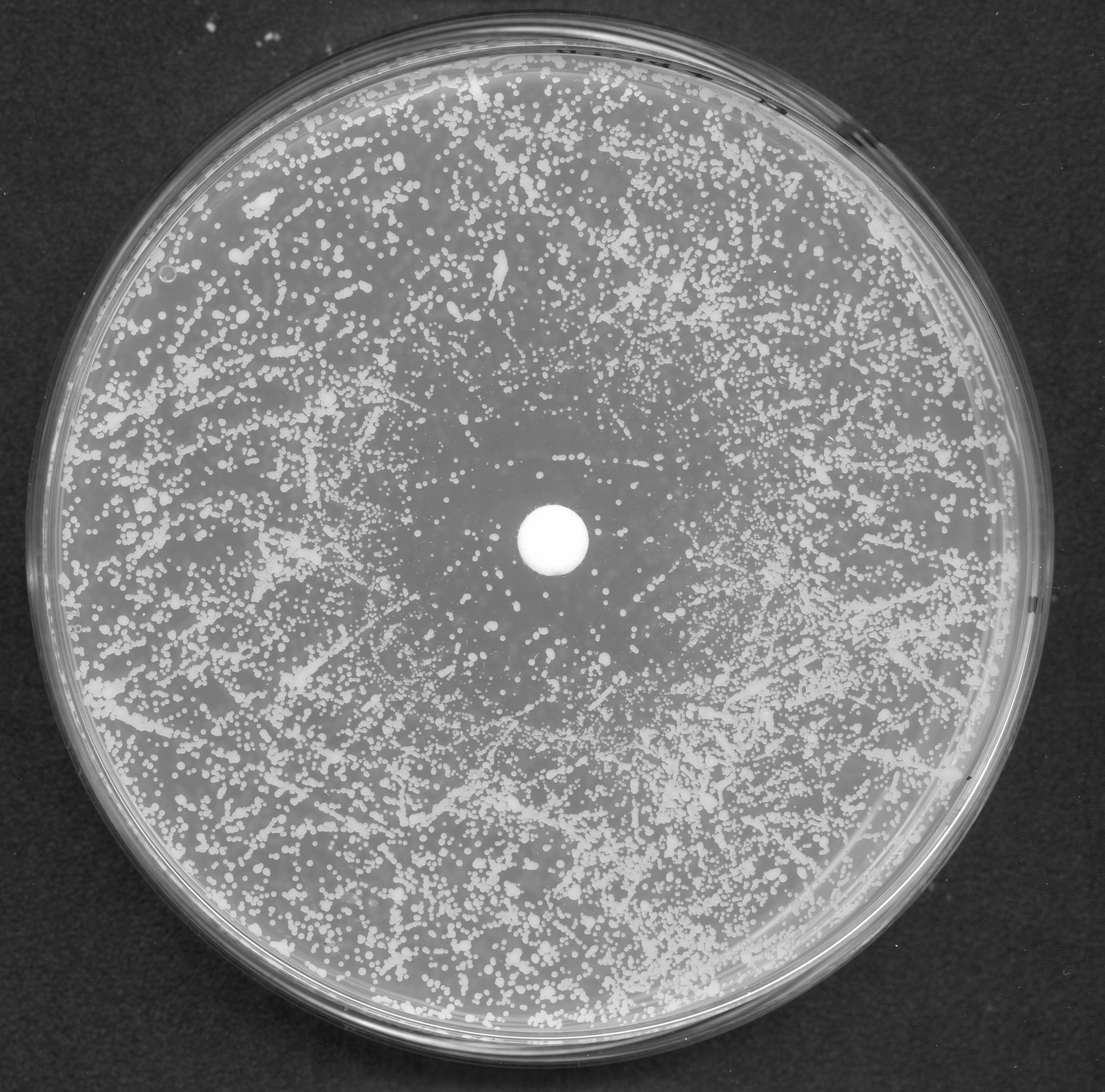

Supplement: Source Data Extended Data Fig. 10 — Unprocessed images for Supplementary Fig. 10d. [file 41564_2022_1072_MOESM21_ESM.zip › Source Data S10/Source Data S10d/MICO_H+_HLUM.jpg]

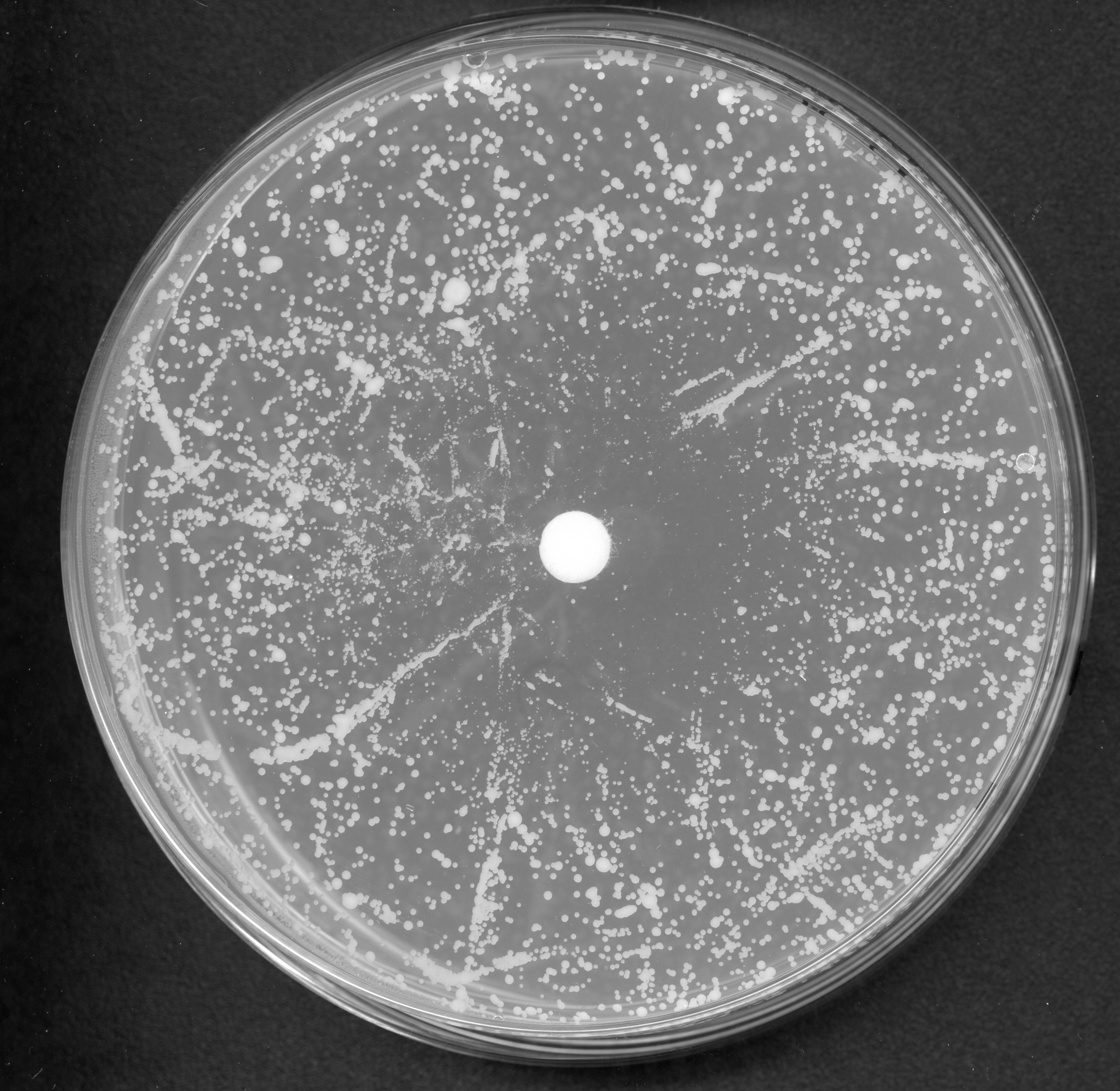

Supplement: Source Data Extended Data Fig. 10 — Unprocessed images for Supplementary Fig. 10d. [file 41564_2022_1072_MOESM21_ESM.zip › Source Data S10/Source Data S10d/MICO_L+_SM.jpg]

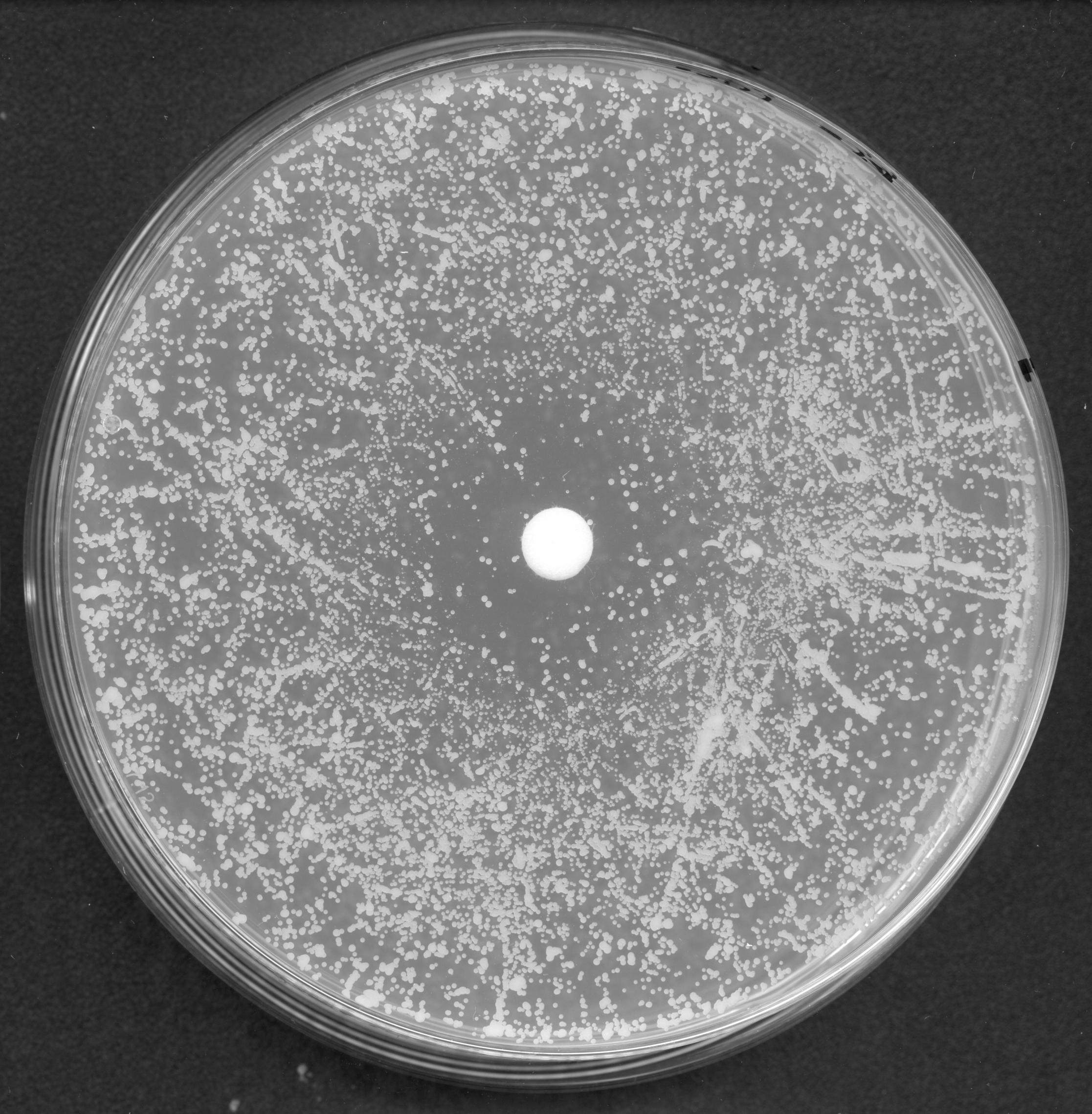

Supplement: Source Data Extended Data Fig. 10 — Unprocessed images for Supplementary Fig. 10d. [file 41564_2022_1072_MOESM21_ESM.zip › Source Data S10/Source Data S10d/MICO_H-_HLUM.jpg]

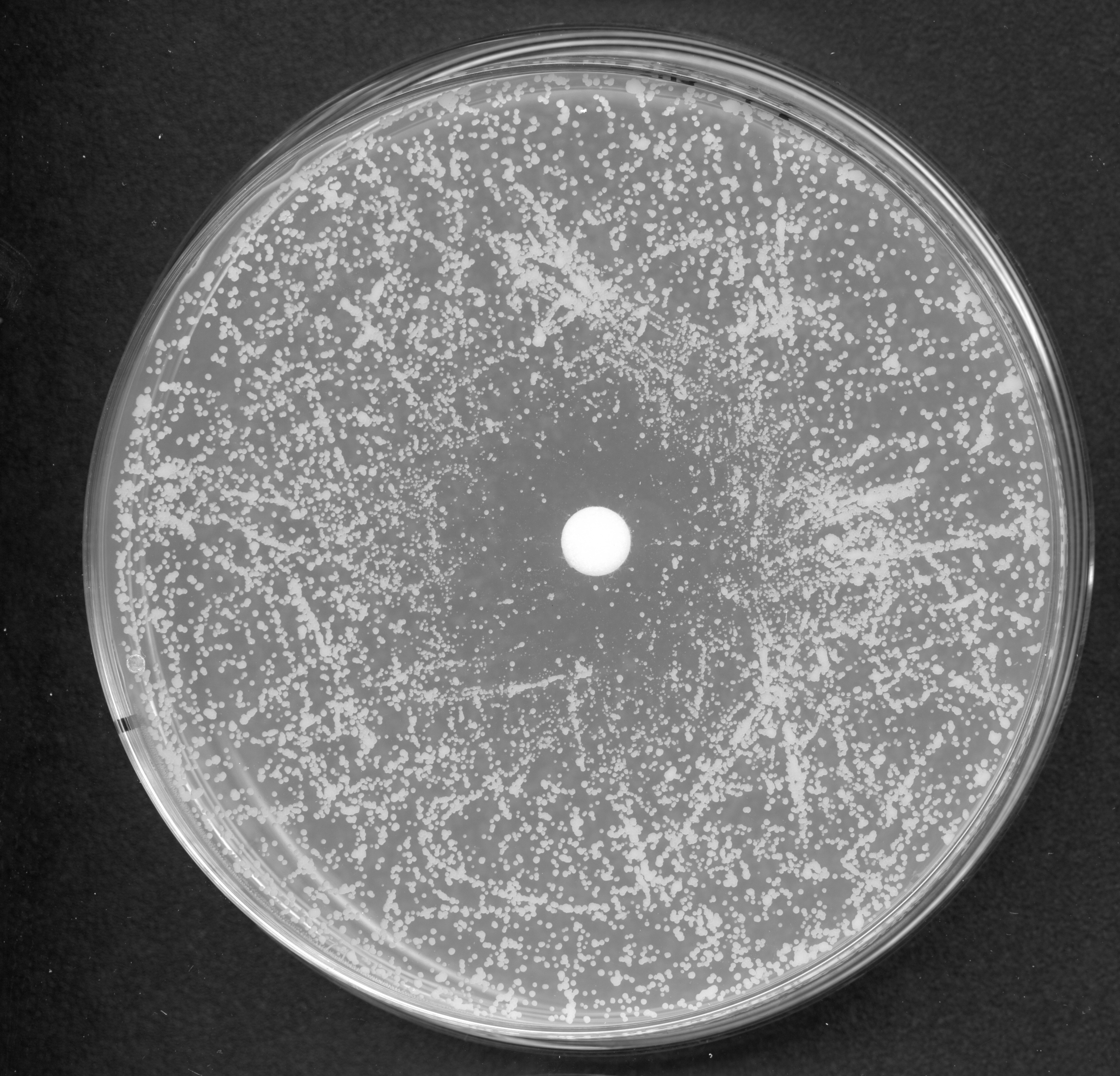

Supplement: Source Data Extended Data Fig. 10 — Unprocessed images for Supplementary Fig. 10d. [file 41564_2022_1072_MOESM21_ESM.zip › Source Data S10/Source Data S10d/MICO_L+_HLUM.jpg]

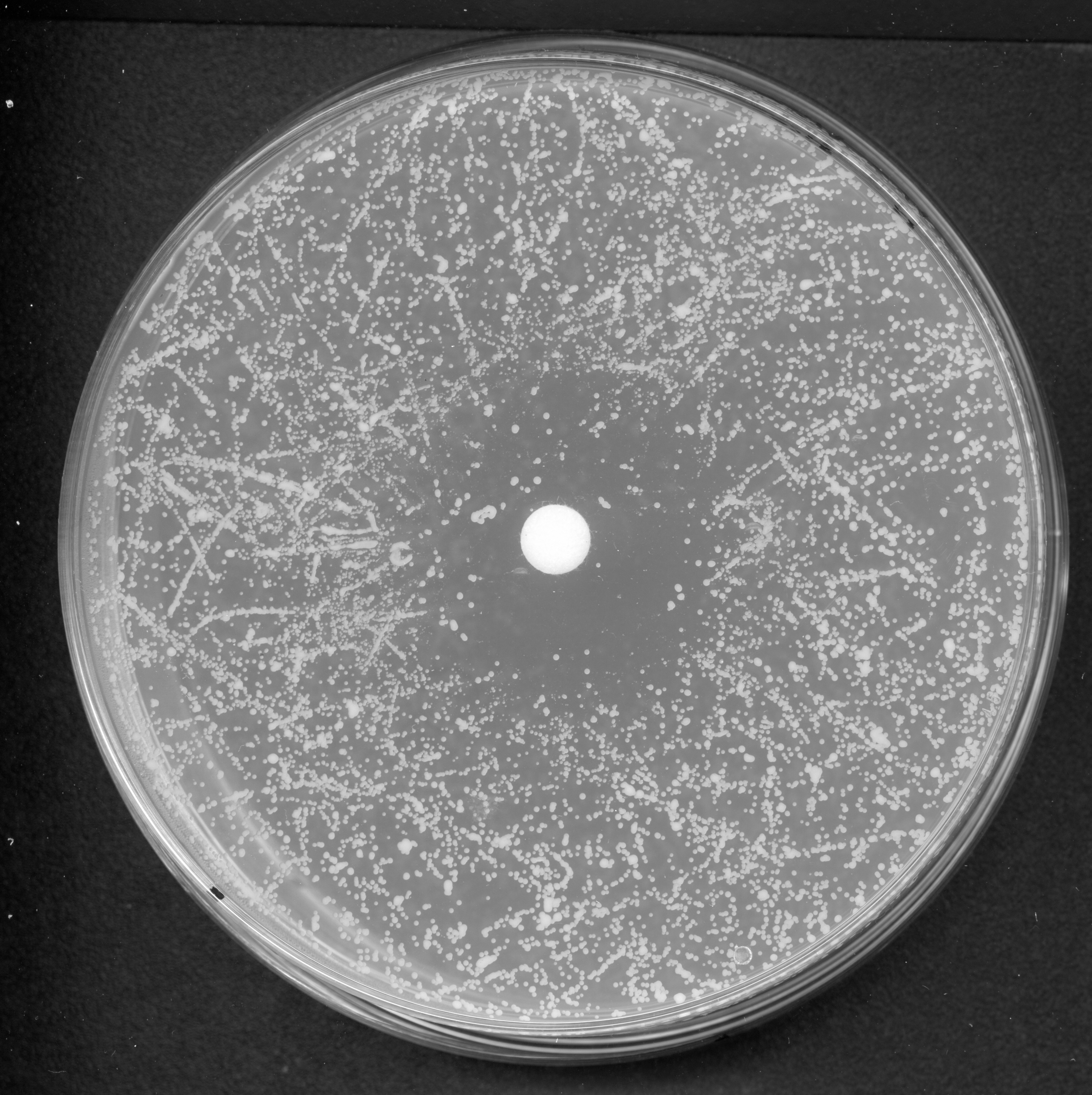

Supplement: Source Data Extended Data Fig. 10 — Unprocessed images for Supplementary Fig. 10d. [file 41564_2022_1072_MOESM21_ESM.zip › Source Data S10/Source Data S10d/MICO_M-_HLUM.jpg]

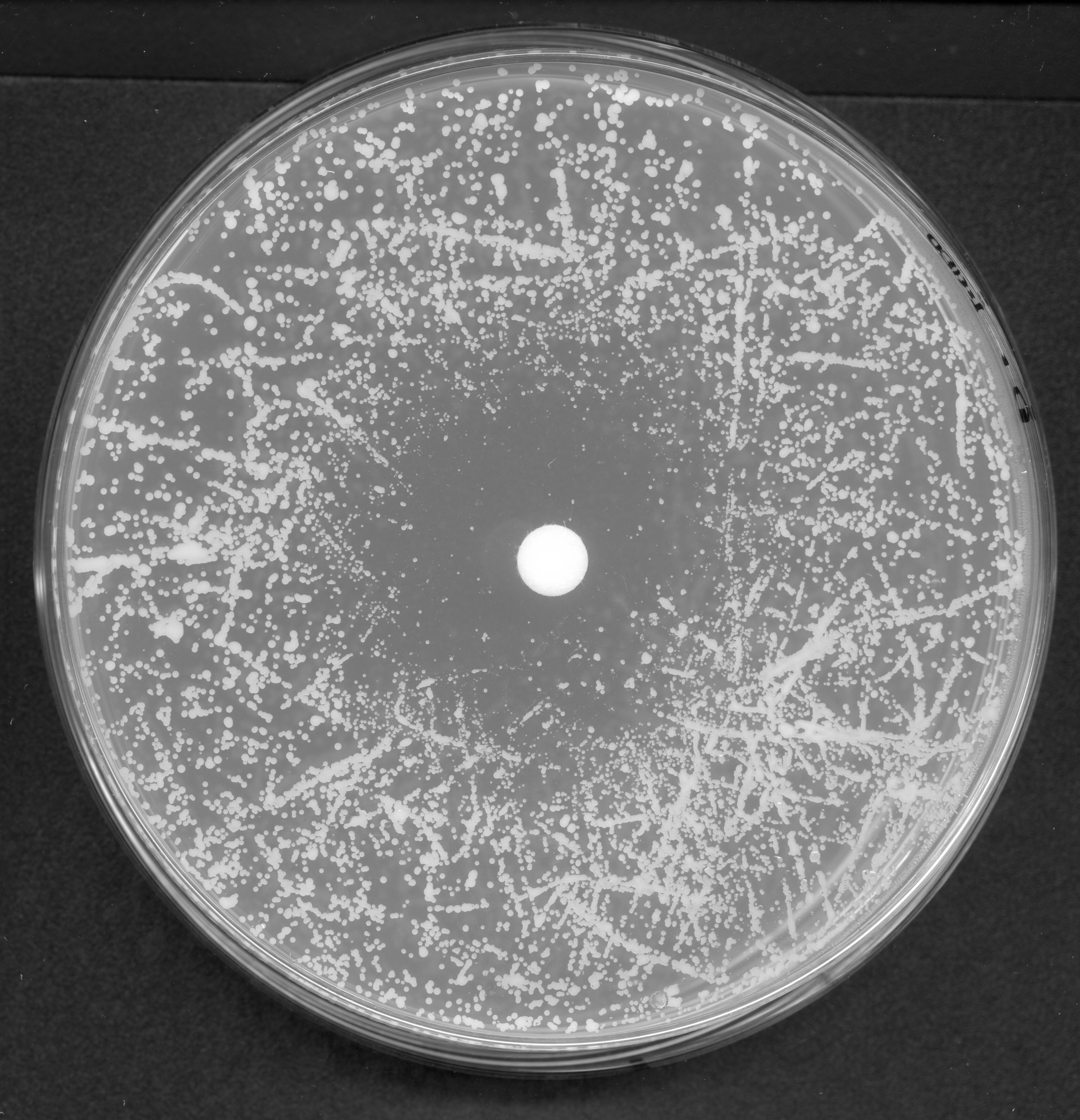

Supplement: Source Data Extended Data Fig. 10 — Unprocessed images for Supplementary Fig. 10d. [file 41564_2022_1072_MOESM21_ESM.zip › Source Data S10/Source Data S10d/MICO_U-_HLUM.jpg]

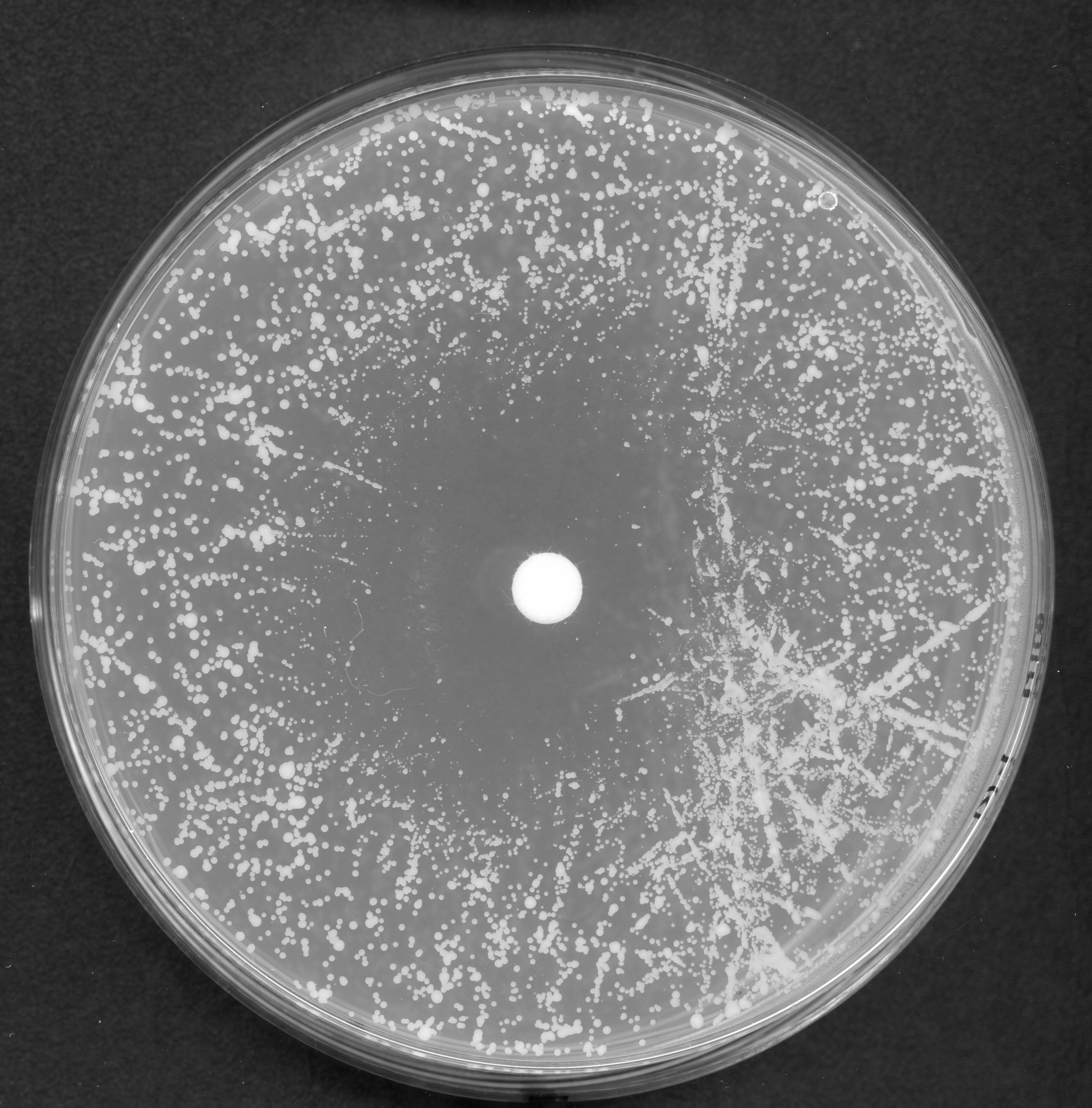

Supplement: Source Data Extended Data Fig. 10 — Unprocessed images for Supplementary Fig. 10d. [file 41564_2022_1072_MOESM21_ESM.zip › Source Data S10/Source Data S10d/MICO_U+_SM.jpg]

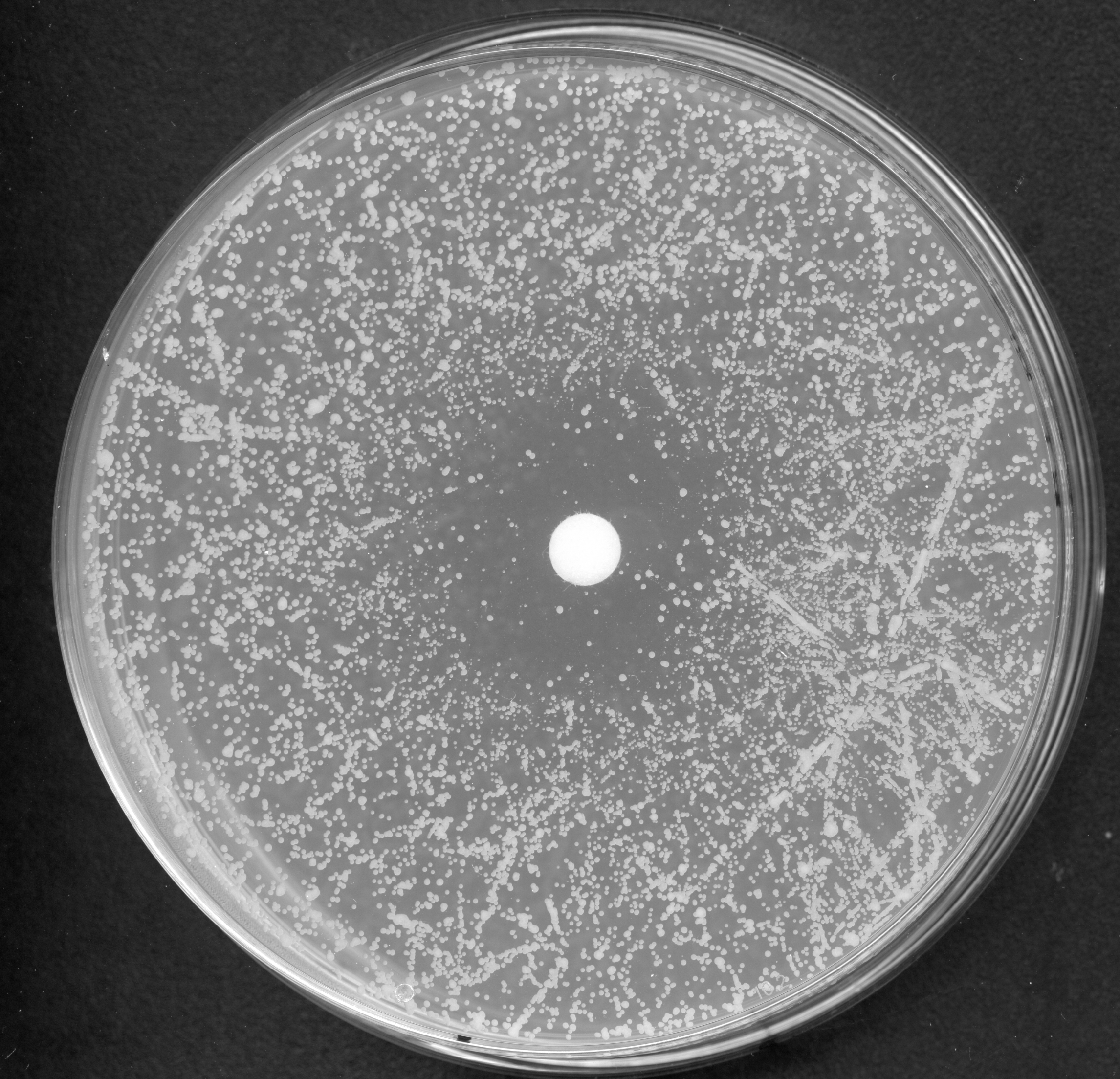

Supplement: Source Data Extended Data Fig. 10 — Unprocessed images for Supplementary Fig. 10d. [file 41564_2022_1072_MOESM21_ESM.zip › Source Data S10/Source Data S10d/MICO_M+_HLUM.jpg]

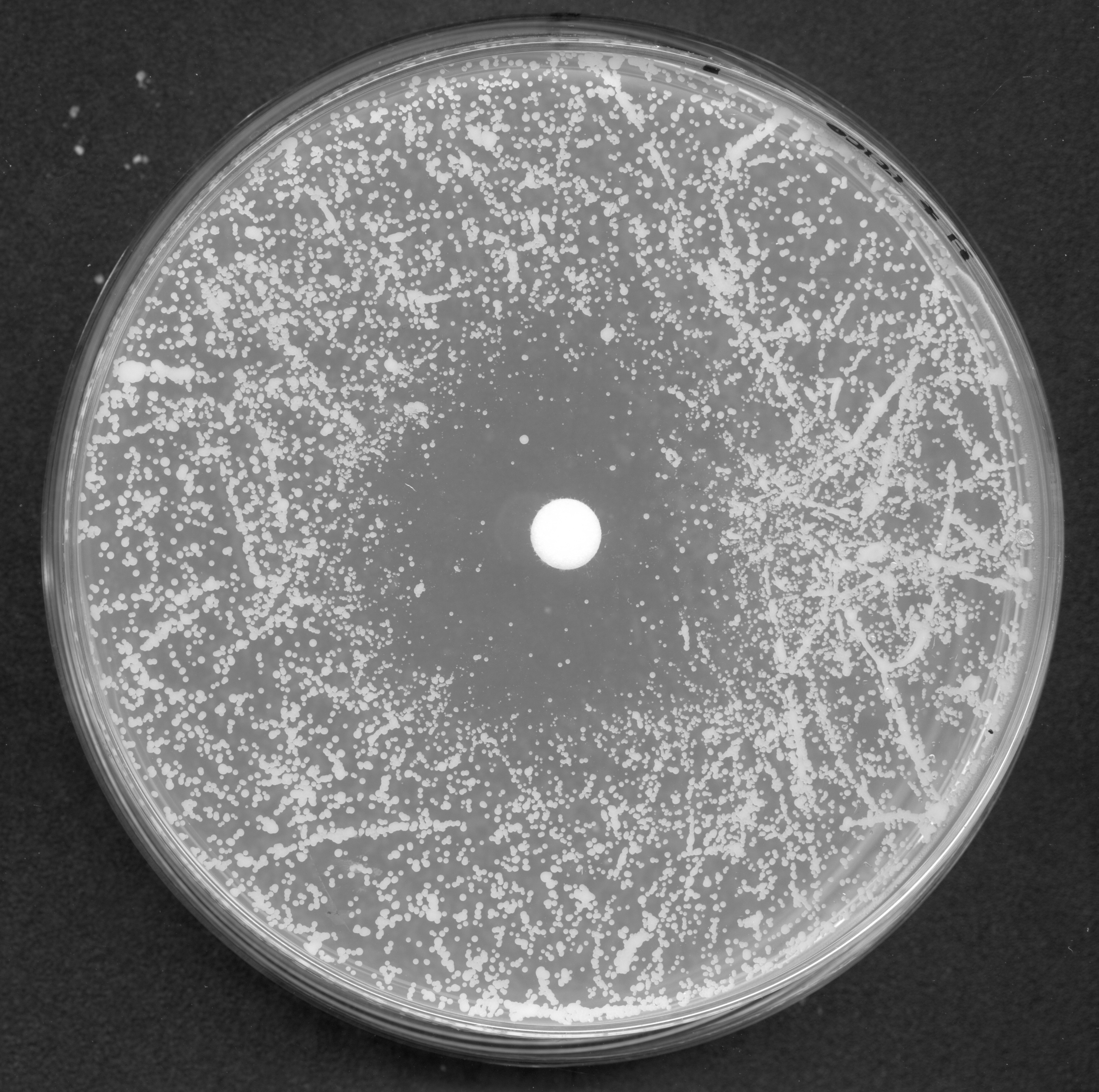

Supplement: Source Data Extended Data Fig. 10 — Unprocessed images for Supplementary Fig. 10d. [file 41564_2022_1072_MOESM21_ESM.zip › Source Data S10/Source Data S10d/MICO_U+_HLUM.jpg]

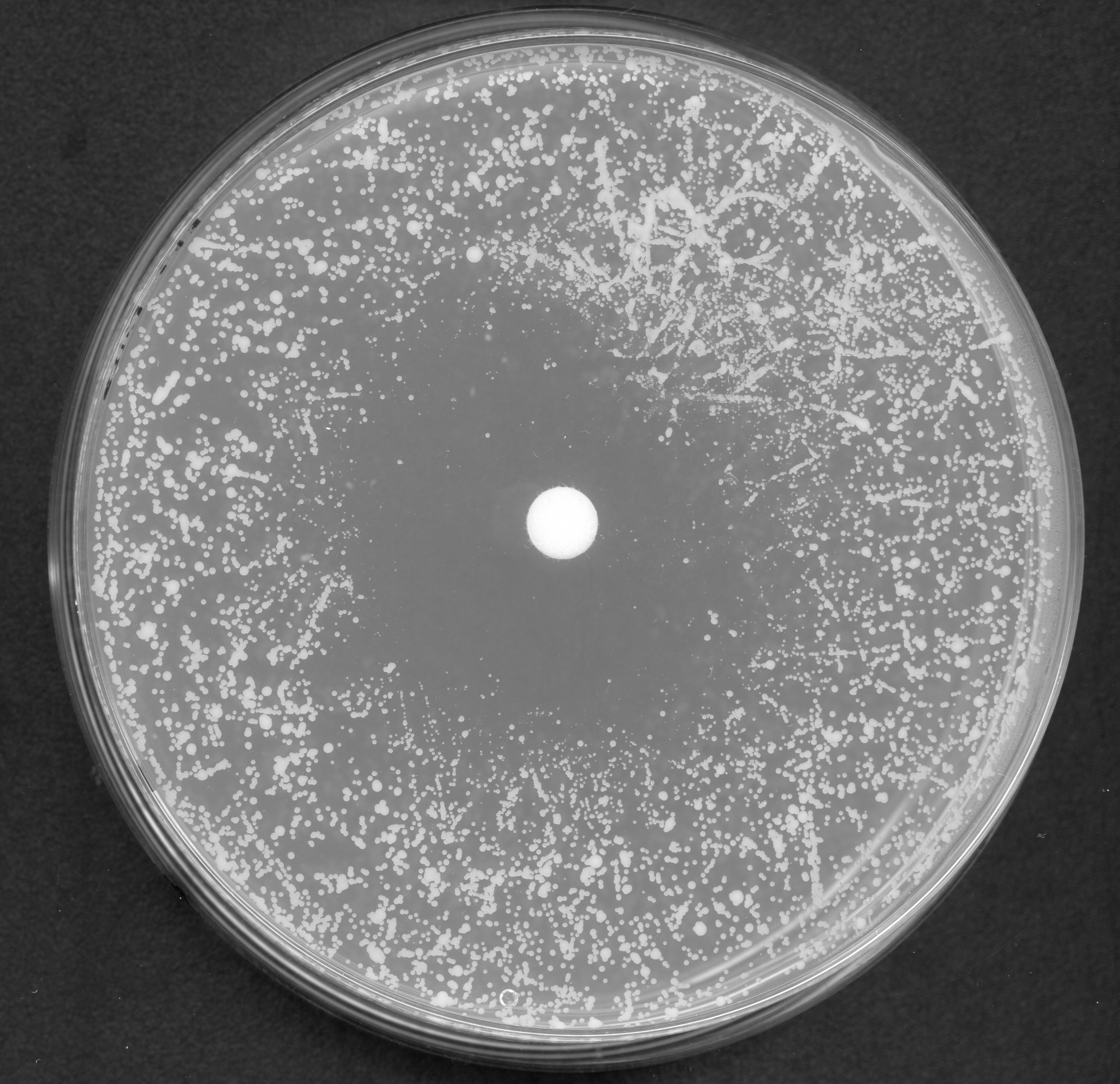

Supplement: Source Data Extended Data Fig. 10 — Unprocessed images for Supplementary Fig. 10d. [file 41564_2022_1072_MOESM21_ESM.zip › Source Data S10/Source Data S10d/MICO_H+_SM.jpg]

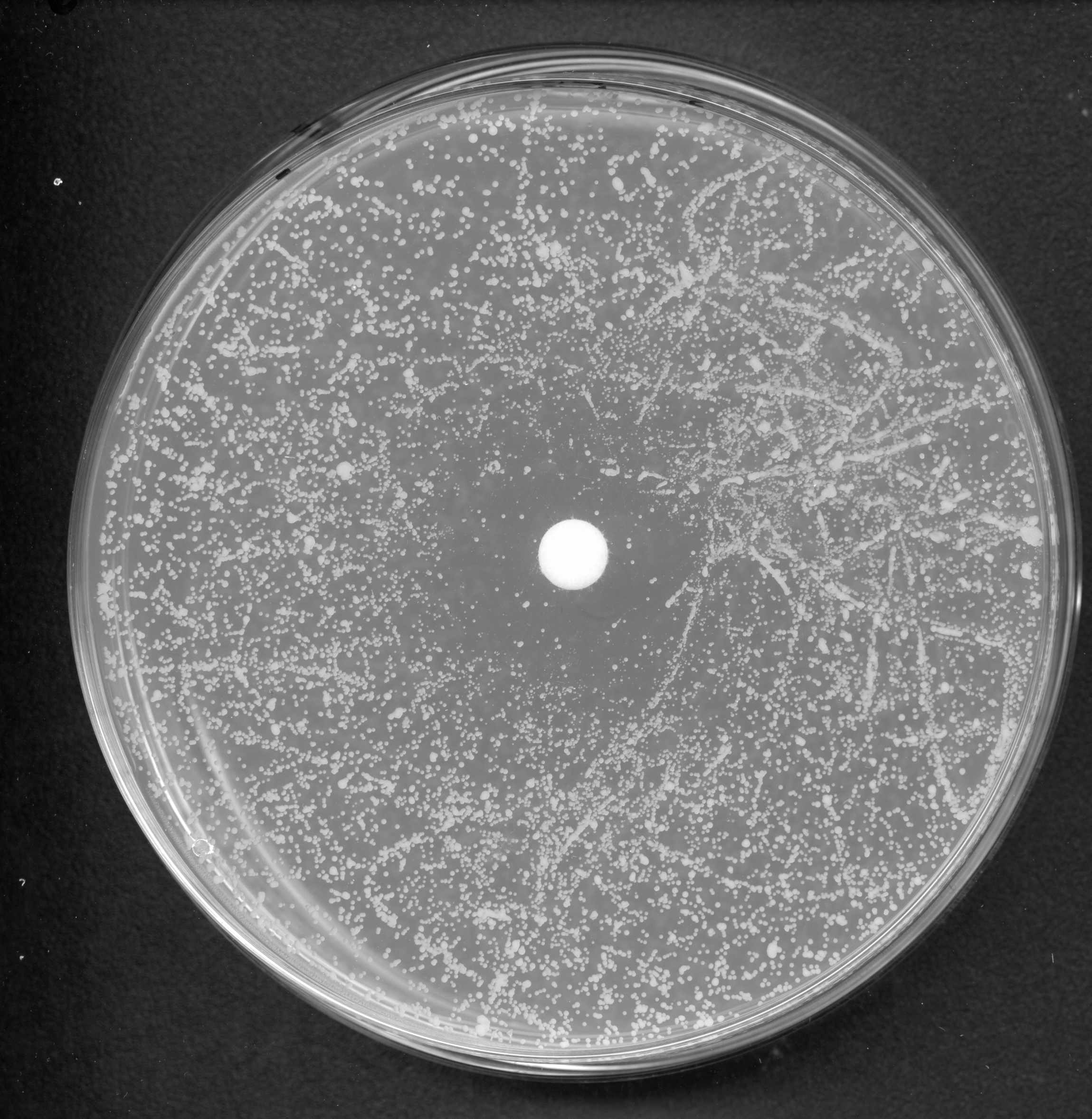

Supplement: Source Data Extended Data Fig. 10 — Unprocessed images for Supplementary Fig. 10d. [file 41564_2022_1072_MOESM21_ESM.zip › Source Data S10/Source Data S10d/MICO_L-_HLUM.jpg]

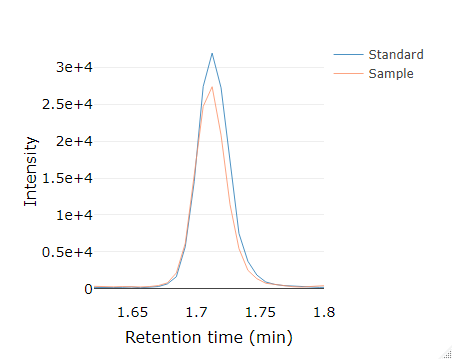

Supplement: Source Data Extended Data Fig. 10 — Unprocessed images for Supplementary Fig. 10d. [file 41564_2022_1072_MOESM21_ESM.zip › Source Data S10/Source Data S10c/tofms_with_label.png]

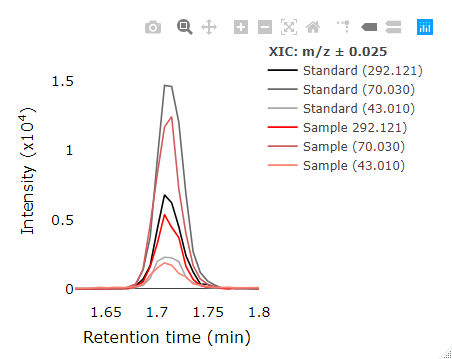

Supplement: Source Data Extended Data Fig. 10 — Unprocessed images for Supplementary Fig. 10d. [file 41564_2022_1072_MOESM21_ESM.zip › Source Data S10/Source Data S10c/product ion_final.png]

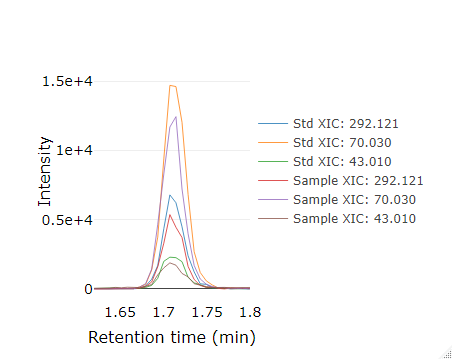

Supplement: Source Data Extended Data Fig. 10 — Unprocessed images for Supplementary Fig. 10d. [file 41564_2022_1072_MOESM21_ESM.zip › Source Data S10/Source Data S10c/product ion_with label.png]
