# Supplementary material for: Microbial communities form rich extracellular metabolomes that foster metabolic interactions and promote drug tolerance
Source: Nat Microbiol. 2022 Mar 21;7(4):542–55. doi: 10.1038/s41564-022-01072-5 (PMC8975748; doi:10.1038/s41564-022-01072-5)
Supplement: Supplementary file 3 — Peer Review Information [file 41564_2022_1072_MOESM3_ESM.pdf]

natureresearch

Peer Review Information

Journal: Nature Microbiology
Manuscript Title: **Microbial communities form rich extracellular metabolomes that foster metabolic interactions and promote drug tolerance**
Corresponding author name(s): Markus Ralser

Reviewer Comments & Decisions:

.

Decision Letter, initial version:

Dear Markus,

Thank you for your patience while your manuscript "Microbial communities form rich extracellular metabolomes that foster cooperation and promote drug tolerance" was under peer review at Nature Microbiology. It has now been seen by our referees, whose expertise and comments you will find at the end of this email. In the light of their advice, we have decided that we cannot offer to publish your manuscript in Nature Microbiology.

From the reports, you will see that while they find your work of some potential interest, the referees raise concerns about the strength of the novel conclusions that can be drawn at this stage. In particular, you will see that referee #1 raises concerns regarding some of the conclusions of the study not being well supported by the data used, and is unclear whether some data is new or previously reported; referee #2 major concern is regarding the use of 'tolerance' while 'resistance' should be used instead; referee #3 raise several major concerns regarding direct comparisons between gut microbes species and the SeMeCo community and how the observations from the SeMeCo community translate to natural microbial communities, among other many other issues. Unfortunately, these criticisms are sufficiently important as to preclude publication of your work in Nature Microbiology.

If you feel that you can address the reviewers' concerns, you can appeal to this decision with a revised manuscript and a point-by-point response. In that case, we could certainly have a second look and reconsider the manuscript if we think the main issues have been addressed.

I am sorry that we cannot be more positive on this occasion, but hope that you find the referees' comments helpful when preparing your paper for resubmission elsewhere.

Yours sincerely,

{redacted}

****
Reviewer Expertise:

Referee #1: metabolic interactions/microbial communities
Referee #2: drug tolerance
Referee #3: microbial ecology/communities

Reviewers Comments:

Reviewer #1 (Remarks to the Author):

This manuscript investigates the role that cross-feeding plays on drug resistance. The authors first present data on resistance to 1,197 drugs for 40 microbes from the human gut. Microbes predicted to be auxotrophic based on genome sequence are found to be less inhibited on average than prototrophic microbes. The authors then show that when co-cultures of prototrophic and auxotrophic yeast are treated with antibiotics it increases the frequency of auxotrophic genotypes. Genome-scale metabolic models predict that co-culturing prototrophs with auxotrophs will lead to increased excretion of metabolites, and changes in the activity of many metabolic pathways. Consistent with this prediction metabolomic and proteomic analysis of yeast co-cultures find significant levels of extracellular metabolites, and some signals of changes in gene expression. Finally, data suggest that auxotrophic cells pump compounds out of cells at

higher rates.

This paper provides extensive interesting data related to the metabolic effects of cross-feeding, and how metabolic interactions alter the impact of antibiotics. However, many of the conclusions drawn from the data do not currently seem warranted (though perhaps many alternative interpretations have been ruled out in ways that are not clear from the writing). A couple of the key issues are:
1) It is not clear to me if the data on resistance for gut microbes is new or summarization of previously reported data.
2) The metabolic modelling approach uses an assumption of group selection that I don't think is warranted in this system. This assumption is the basis for the high levels of metabolite sharing that are predicted.
3) The conclusion that cross-feeding provides a competitive advantage that can not be exploited is inaccurate.
4) The text seems to imply that auxotrophy not only increases survival in the presence of antibiotics, but that auxotrophs also protect prototrophs. I do not understand how this conclusion is drawn from the data.

Specific comments.
Line 93 – The authors might consider softening the language in this sentence to reflect that while most microbes have not yet been isolated in monoculture in the lab, it may be readily possible on the correct medium. Indeed, if all that microbes require is metabolites like amino acids it should be feasible to generate media with the correct supplements for most species.

Line 98 – I have trouble following the logic here. It seems that the previous sentences describe why prototrophs should be outcompeted by auxotrophs, but this sentence seems to mix a benefit for auxotrophs (Black Queen) and a benefit of prototrophy (bet hedging). In the next sentence bet hedging is referred to as a promoting auxotrophy and I don't understand this rationale. Shouldn't maintaining the ability to metabolize many compounds provide a benefit for prototrophy in variable environments?

Line 116 – Auxotrophy might provide a benefit to an auxotrophic cell, but it is not clear to me why it would protect the rest of the community.

Line 148 – The description in the text makes it sound like the average number auxotrophic species across communities, but from the figure I think the percentages actually represent the percent of communities that have more auxotrophs than prototrophs. This needs to be clarified.

Line 152 – Are the 40 species all bacteria or a combination of bacteria, yeast and protists?

Line 159 – First it is not clear if this is new data or simply restating data collected in the previous paper. Second it needs to be made clear that you are taking the AUC with antibiotics relative to AUC without antibiotics. Further it would be helpful to know how AUC in the absence of antibiotics compares for prototrophs and auxotrophs. Is the difference in resistance a function of growth rate rather than auxotrophy per se?

Line 165 – Did the auxotrophs actually outgrow the prototrophs are just suffer a lower reduction in growth relative to no antibiotic conditions. This relates to the point above about knowing the relative growth of auxotrophs and prototrophs in the absence of antibiotics.

Line 173 – I think it is more common to use tolerance to refer to death rate in the presence of antibiotics, and resistance as the ability to grow in the presence of antibiotics. I would recommend at least clearly defining how you are using the term.

Line 194 – I would find it easier to first see a simple bar graph showing the

Line 196 – How were the clusters determined? The figure legend says based on correlation, but correlation of what?

Line 198 – It seems possible that the change in ratio of auxotrophs could also be driven by antibiotics altering the fidelity with which plasmids are transmitted to offspring. How have you ruled out this possibility?

Line 208 – By "vehicle-alone" do you mean the system in the absence of antibiotics?

Line 211 – Maximizing community growth rate assumes that auxotroph and prototroph pairs have been under group selection that has led to the evolution of cooperation. I don't see that this selection was actually imposed in the system, and therefore the assumption does not seem justified. Rather I would expect the auxotroph and prototroph to still be optimizing individual interests. I would predict that release of metabolites is driven by i) suboptimal metabolic behavior as a result of recent gene loss, or ii) is a result of leakiness imposed by amino acid limitation. Suboptimal metabolic behavior could potentially be assessed with Minimization of Metabolic Adjustment (MOMA- DOI: 10.1073/pnas.232349399). I would also strongly encourage using dynamic flux balance analysis with the growth of each strain optimized independently.

Line 237 – It is really interesting that different metabolites are measured in the SeMeCo relative to a wildtype. How is the impact that plasmids have in SeMeCo differentiated from the impact of auxotroph presence? Also what was the abundance of auxotrophs in these experiments? To the extent that auxotrophy alters metabolism (rather than plasmid presence), I wonder if the alteration is due to the loss of a gene or due to the cell being limited by an amino acid (for example if SeMeCo were grown in media supplied with the amino acids would you see the same changes in metabolism?). To reiterate the comment above I do not think that the observation of metabolic shifts in SeMeCo validates the modelling assumption that cells have evolved to cooperate. Rather I think the model is generating a similar observation of metabolite exchange for the wrong reasons.

Line 270 – I don't think that this data demonstrates that prototrophs were responding to the presence of the auxotrophs. It simply indicates that there was a difference in expression between prototrophs and auxotrophs.

Line 281 – I am confused about the comparison that is being made here. Is expression being compared between prototrophs grown with auxotrophs and prototrophs grown with wildtype? Or are prototrophs being compared against wildtype? What are the auxotrophs being compared against? I find the text and figure 3 highly confusing.

Line 294 – It seems like this sentence is implying that auxotrophs make the prototroph grow faster than it would in the absence of the auxotrophs, but the experiment that follows is instead testing the growth rate of the prototroph relative to the auxotroph.

Line 323 – Was cell sized controlled for? If auxotrophic cells are smaller than they would fluoresce less but not because of a change in pumping activity.

Line 336 – It isn't clear to me here or in the supplement how concentration was determined. Were the number of cells in each sample accounted for?

Line 342 – I don't understand didn't you just show that adding amino acids increases the resistance in yeast? Why are you arguing here that the difference is resistance is driven by genotype rather than by media?

Line 394 – This in essence just sets up a mutualism where each genotype is providing a costly benefit rather than just one of the genotypes providing a costly benefit. Mutualisms are still subject to exploitation. A pair of strains that exchange metabolites might grow faster than a pair of strains that don't exchange metabolites. However if you introduced a cheater into the original pair that could use the metabolites and not providing anything in return it should have a growth advantage and be able to outcompete a

cooperating strain. To avoid this you would need a mechanism such as spatial structure that generates a correlation between the amount a genotype cooperates and how much it gets in return.

Line 410 – What is the data supporting this statement? Is it increased uptake or increased excretion that leads to the effect?

Line 414 – It might be interesting to contrast this speculation with the observation in (https://doi.org/10.1371/journal.ppat.1008700).

Line 619 – I don't understand how information on gene essentiality was used. Was this used simply to improve the S. cerevisiae model that was used? Counting a gene as essential because it reduces growth by 10% seems like quite an extreme cutoff. Also S. cerevisiae should be italicized in line 620.

Reviewer #2 (Remarks to the Author):

The authors explore the differential response of auxotrophs and prototrophs under the exposures to various drugs by measuring the growth of each type in communities. First, they show that auxotrophs are enriched in many host-associated communities and measure the enrichment of auxotrophs during growth under various drugs. Building on their previously established SeMeCos system which reaches a stable co-existence of autotrophs and phototrophs, they also measure the enrichment of auxotrophs under various drugs in this system. They find that, similarly to the results in natural communities, auxotrophs growth better, especially under drugs that have a strong effect in reducing the growth such as azole antifungals and statins. The better growth under the drug means that the auxotrophs are more resistant (see comment 1 below). The metabolome data shows that the presence of auxotrophs results in enrichment of the extracellular environment by increased concentrations of amino acids. The authors then attribute the higher resistance of auxotrophs under drugs to the increased concentrations of amino acids. Accordingly, they show that both auxotrophs in SeMeCo and wt communities are also more resistant to azoles when grown in the presence of HLUM supplementation. This suggests that the metabolism involved in the uptake of amino acids results in a higher resistance level. In order to understand this point, the authors use a dye that enables to measure the level of efflux on single cells and show that auxotrophs have a lower level of the dye. They then directly measure the intracellular level of azole and show that it is lowered in auxotroph, explaining their higher resistance.
The results are interesting and add to the body of works showing how microbial communities can lead to different antibiotic susceptibility profiles when grown together. The metabolic effect that they uncover should be interesting to a broad community.
Main comment 1: the authors term the effect that they measure "tolerance" while they measure the impairment in growth. They describe as "tolerant" strains that grow better under the drug. However, tolerance is typically used for cidal treatments. A strain that grows better at the same drug concentration is simply considered as more resistant. Here all data seem to show that in the conditions in which the experiment are performed, the MIC of auxotrophs is higher. The rest of the findings agree well with this view: resistance mechanisms (higher efflux) do lower the effective concentration of the drug (which is reflected in the higher MIC). Replacing all instances of "tolerance" by "resistance" would make this clearer. Otherwise, the authors would need to show that the MIC does not change for example for the wt with and without HLUM supplementation. According to the DDA, the resistance level is fact increased, showing resistance. The findings are no less interesting if amino acid metabolism as seen in auxotrophs is shown to lead to resistance but then the work should be put in the context of induced resistance.

Minor comments:
- It is difficult to compare the results obtained on the microbiome communities and the SemeCos as they are presented in very different formats. It would be helpful to translate the data obtained on the communities (AUC) into the simple parameters used for the SemeCos (for example the enrichment of

auxotrophs as shown in Fig.1)
- Many of the significance tests turn out to be non-significant in Fig. s1. Please mention how the significance was made to take into account the multiple hypotheses tested.

Reviewer #3 (Remarks to the Author):

Microbial communities display a variety of emergent properties not present in individual species, including robustness to challenge by antimicrobials. Communities are often supported by metabolite exchange between individual species, and auxotrophs (species that lack essential metabolic pathways) have been found in a wide range of microbiomes. The mechanisms that allow auxotrophs to persist, despite their reliance on other community members for essential nutrients, remain unclear. Mysteries include why “cheating” by auxotrophs does not tend to destabilize microbial communities and why prototrophs (which can downregulate biosynthetic pathways through feedback mechanisms) coexist with auxotrophs rather than outcompeting them.
In this article the authors show that auxotrophic species are particularly frequent in certain kinds of microbial communities and are more drug resistant than their prototrophic counterparts. They use a model isogenic auxotrophic community of S. cerevisiae yeast (SeMeCo) to experimentally validate the increased drug resistance of auxotrophs. Metabolic reconfiguration by auxotrophs results in system wide changes in flux that alter the intracellular metabolome as well as metabolites exported leading to an enriched communal environment. This enriched environment produces a reciprocal response from prototrophs. Increase efflux and the enriched environment lead to stronger drug tolerance.
Overall the authors use the SeMeCo community to good advantage, in combination with metabolic modeling, to explore why auxotrophs confer antimicrobial resistance in microbial communities. I recommend that the following comments be addressed before publication:

Major Comments:
• Please provide more details on how the auxotrophies were calculated, beyond referring to Reference 38.
• I interpret Figure 1A as all communities examined have auxotrophies, but some have more that prototrophs. Is this correct? Or is there a “0” location on the plot? Do any communities contain no auxotrophs?
• Can you further discuss auxotrophies that appear to be the exception, for example leucine? Also histidine (lines 266-268) Also in Figure 2 why do certain amino acids increase and not others, is it related in any clear way to the different auxotrophies?
• Throughout the authors draw comparisons between single human gut microbiome species tested individually and the SeMeCo community. Is this reasonable?
• Is there any reason to expect that yeast auxotrophs (which have not evolved to have these auxotrophies in specific communities) will behave differently than naturally occurring auxotrophs? This is worth discussing in light of generalizing the paper’s results. Do you expect the same “nutrient overflow” and metabolic enrichment for naturally occurring auxotrophs?
• Would a spent media experiment, or similar, verify that it is the metabolically enriched environment that leads prototrophs towards more uptake and hence enhanced drug resistance?
• Do the tolerance experiments (Figure 3) demonstrate that it is increase uptake that leads prototrophs towards drug tolerance? Could it simply be growth rate differences on supplemental amino acids or other metabolic changes? Is there a way to directly show that increasing uptake leads to drug tolerance, especially in light of the fact that this seems to conflict with the increased export mechanism in auxotrophs?
• Is there anything about the mechanism of action of different drugs, given that the hypothesis here is that efflux is regulated to increase drug tolerance, that would allow one to predict a priori which drugs should affect auxotrophs less than prototrophs? Does this match with the data from the drug libraries screened in this research?
• Would it follow from this work that prototrophs are fitter in the presence of other prototrophs under drug-treatment conditions (due to prototrophs “sucking up” more drug than auxotrophs, leading to an overall lower average dose per cell)? Is this testable or interesting?

• Should I view drug tolerance in microbial communities containing auxotrophs as an emergent property or as a direct function of the number of auxotrophs and hence more drug resistant species in the community? How does this translate to natural microbial communities, and in particular, to resilience of community composition? Would populations of purely cooperative auxotrophs be the most drug resistant? Or is it again a function of the proportion of the most drug resistant auxotroph in the community?

Minor Comments:

• What is shown in Figure 1b? From reading the Figure caption, I think that it is showing the total number (out of 15 auxotrophic gut species) that had a leu auxotrophy, trp auxotrophy, etc. But in the text (Line 155) it seems to suggest that I can get from Figure 1b information about the number of auxotrophies per species. This is how the data is plotted in S1B though, so perhaps that is what you mean.
• Please clarify what is being plotted in Figure 1c. Is this the 40 gut microbiome species from Reference 39 (and indeed, is this data from Reference 39 reused/replotted for this study?)
• How was the drug concentration chosen (Line 190)?
• It seems strange to me to put the inset (e(i)) with the SeMeCos data since (i) is from the gut microbiome mostly bacterial strains and € is the yeast data.
• Figure 1f: Is the heat map reversed? Red means a higher prototroph to auxotroph ratio, therefore fewer auxotrophs? Which seems the opposite of what the Figure is trying to show? I could just be confused. OR should I be comparing color changes across the number of auxotrophies (i.e. less red for orange and purple populations). Also should the description explicitly state that P:A ratio stands for prototroph:auxotroph ratio?
• Line 150: "host-associated"
• Line 268 "shows"
• Line 398-390: "More drug tolerant"
• Line 393 and Lines 400-402: I think it would be helpful to mention the specific metabolites as part of this discussion.
• Line 427: cause vs causes
• Line 436: foster and improve
• In figure 2C it would be easier to compare the figures if the y-axis was the same.
• Line 801: delete "containing"
• Line 819: "prototrophs"
• Line 823-824: "...HLUM as opposed to…"

Author Rebuttal to Initial comments

Response to Reviewers' Comments

We would first like to thank all reviewers for their insightful comments and constructive suggestions that have greatly helped us to improve the manuscript. We have now addressed all of the points raised through additional experiments and/or by editing the text for clarity. A point- by-point response to all comments is provided below.

Reviewer #1 (Remarks to the Author):

This manuscript investigates the role that cross-feeding plays on drug resistance. The authors first present data on resistance to 1,197 drugs for 40 microbes from the human gut. Microbes predicted to be auxotrophic based on genome sequence are found to be less inhibited on average than prototrophic microbes. The authors then show that when co-cultures of prototrophic and auxotrophic yeast are treated with antibiotics it increases the frequency of auxotrophic genotypes. Genome-scale metabolic models predict that co-culturing prototrophs with auxotrophs will lead to increased excretion of metabolites, and changes in the activity of many metabolic pathways.

Consistent with this prediction, metabolomic and proteomic analysis of yeast co-cultures find significant levels of extracellular metabolites, and some signals of changes in gene expression. Finally, data suggest that auxotrophic cells pump compounds out of cells at higher rates.

This paper provides extensive interesting data related to the metabolic effects of cross-feeding, and how metabolic interactions alter the impact of antibiotics. However, many of the conclusions drawn from the data do not currently seem warranted (though perhaps many alternative interpretations have been ruled out in ways that are not clear from the writing).

We thank the reviewer for the detailed suggestions, which have been either implemented or addressed with improved writing:

1) It is not clear to me if the data on resistance for gut microbes is new or summarization of previously reported data.

The reviewer's question concerns only Fig. 1a,b; other figures are based on data that has been recorded in our laboratory specifically for the questions that emerged as part of this study. In Fig. 1a-b, we show results that derive from the re-analysis of large-scale data resources and metabolic models [1,2], namely the Earth Microbiome Project (EMP) [2] and a resource paper by Maier et al [1]. In both cases, we determined which species are auxotrophic and prototrophic, and compared them. These data resources have not been previously used for this purpose, and we believe the use of data available as part of publicly available large-scale datasets was the best and least biased solution to ask the 'general' question that we address in our manuscript.

More specifically, we used the datasets generated in Maier et al. [1], containing growth information on 40 gut microbiome species treated with >1000 drugs, and used the auxotrophy predictor developed by Machado et al [3]. We first asked about frequency and nature of the auxotrophs. Secondly, we asked what were these auxotrophies and if they played a role in drug response.

Furthermore, using the auxotrophy predictor [3] we identified the frequency of auxotrophic species in the EMP [2], comprising thousands of samples and associated metadata spanning diverse environments types, geographies and chemistries. From both analyses, we concluded: 1) auxotrophies are highly prevalent in natural microbial ecosystems, particularly in host-associated communities; 2) there are a number of gut microbiome species auxotrophic for the biosynthesis of amino acids, and 3) that these gut auxotrophic species exhibit better growth under drug stress than their prototrophic counterparts.

We apologize for the lack of clarity in the previous manuscript, and have now revised the text accordingly.

2) The metabolic modelling approach uses an assumption of group selection that I don't think is warranted in this system. This assumption is the basis for the high levels of metabolite sharing that are predicted

We thank the reviewer for bringing this unclarity to our notice. We would like to emphasize that the uptake of histidine, leucine, uracil and methionine by the respective auxotrophs, is not an assumption, but a well established and robust experimental result. The modelling approach we use - namely flux balance analysis - requires an objective function to simulate flux distribution in a system (in this case the SeMeCo community in which the different auxotrophs grow based on metabolite exchange interactions). The FBA model does not assume that other metabolites than the ones for which a subset of the the cells possess the auxotrophies, need to be exchanged for growth.

We use the sum of biomass of the cell types in question as an objective function. While this indeed may be interpreted as a group selection (not in the evolutionary sense though), the purpose of this formulation here is simply to recapitulate the experimental observation that the different cell types co-proliferate in the SeMeCos; the only source for the auxotrophic metabolites (H, L, U and M) within the community are the (prototrophic) cells that can synthesize them [4]. Thus, we do not intend any selection per se but only aim at estimating metabolic fluxes in the system using experimental observations. We have now clarified this in the manuscript. We have also re-written the manuscript, so that the discussion about the ecological-evolutionary implications are separate from the discussion of the actual metabolic properties of auxotrophy-prototrophy interactions inside the community, and hope this avoids further confusion.

We would like to emphasize at this point that the metabolic properties we discover as part of our study, can indeed be explained by the individual cell's optimisation of metabolism (i.e. we do not assume they need to emerge due to auxotroph-prototroph co-evolution). The basis for the metabolite exchange interactions are intrinsic metabolic properties of microbes, in particular their ability to feed-back inhibit their biosynthetic pathways to efficiently uptake and exploit extracellular metabolites once they are available in the extracellular space. Also the reconfigurations in the metabolic network that are responsible for the altered overflow metabolism when cells switch from self-synthesis to the uptake of the amino acids, are explained by the structure of the metabolic network, that is largely due to the thermodynamic and reaction properties of the interconverted

metabolites. To avoid any confusion, we have substantially restructured the introduction and discussion: and now separate the evolutionary implications of our findings, strictly from the operational properties of the communal metabolic network, that we study herein.

3) The conclusion that cross-feeding provides a competitive advantage that can not be exploited is inaccurate.

We agree with the reviewer that this statement was too strongly worded, and also not applicable in the context of, e.g., invading species. We did not mean to say that cells cannot lose exported metabolites to competitors or invaders, but wanted to highlight that our data shows that there is a metabolic cost for a cell to uptake a metabolite from a shared metabolite pool. These costs emerge due to the metabolic reconfiguration that the metabolite uptaking cell needs to undergo. These costs, i.e. the efflux of other metabolites, is due to the topological organization of metabolism, that is represented in the genome scale metabolic network reconstruction that we use in the FBA analysis. The situation that auxotrophs return metabolites to the communities for taking up the metabolites that are essential to them, is something of importance that has not been appreciated before. To correct for our misleading statement, we have now thoroughly re-written the section.

4) The text seems to imply that auxotrophy not only increases survival in the presence of antibiotics, but that auxotrophs also protect prototrophs. I do not understand how this conclusion is drawn from the data.

We apologize if the discussion of our results was confusing. Our results show that not only auxotrophs gain robustness to drug exposure when they uptake specific metabolites, like H,L,U,M, but also prototrophs (Fig. 2 & Fig. 4). Indeed, we expected that all properties we detect in auxotrophs can also be observed in prototrophs; auxotrophs have less, not more, metabolic capacities than prototrophs.

We derive this discussion point based on the following results:

- The presence of auxotrophs increases metabolite concentrations outside of all cells (auxotrophs and prototrophs) in the colonies (Fig. 2)
- An increase in the metabolite concentrations outside of cells decreases the effect of antifungals also on prototrophs (Fig. 2 & Fig. 4).

We have now conducted additional experiments, which illustrate the prototroph phenotype more comprehensively. We titrated the concentration of histidine, leucine, uracil and methionine (HLUM 0-10x relative to the standard media supplementation). This analysis revealed that higher extracellular metabolite concentrations lead to better growth of prototrophic wild-type cells in the presence of the drug, as evidenced by increased growth within (used as a measure of drug tolerance) and shrinkage of the zone of inhibition (used as a measure of resistance) [5] in disk diffusion assays (DDA) performed with miconazole (Supplementary Fig. S4). We also confirmed this using a liquid growth assay where we titrated HLUM supplementation against increasing azole concentration. In agreement with the DDA, we observed increased area under the curve

(AUC), reflecting increased growth, with increasing HLUM supplementation (Fig. 2e). Furthermore, no significant growth changes were observed with increasing concentrations of HLUM in the absence of drug, indicating that the drug tolerance response is independent of the growth promoting effects of metabolite supplementation. We have now included these new data and adapted the text accordingly.

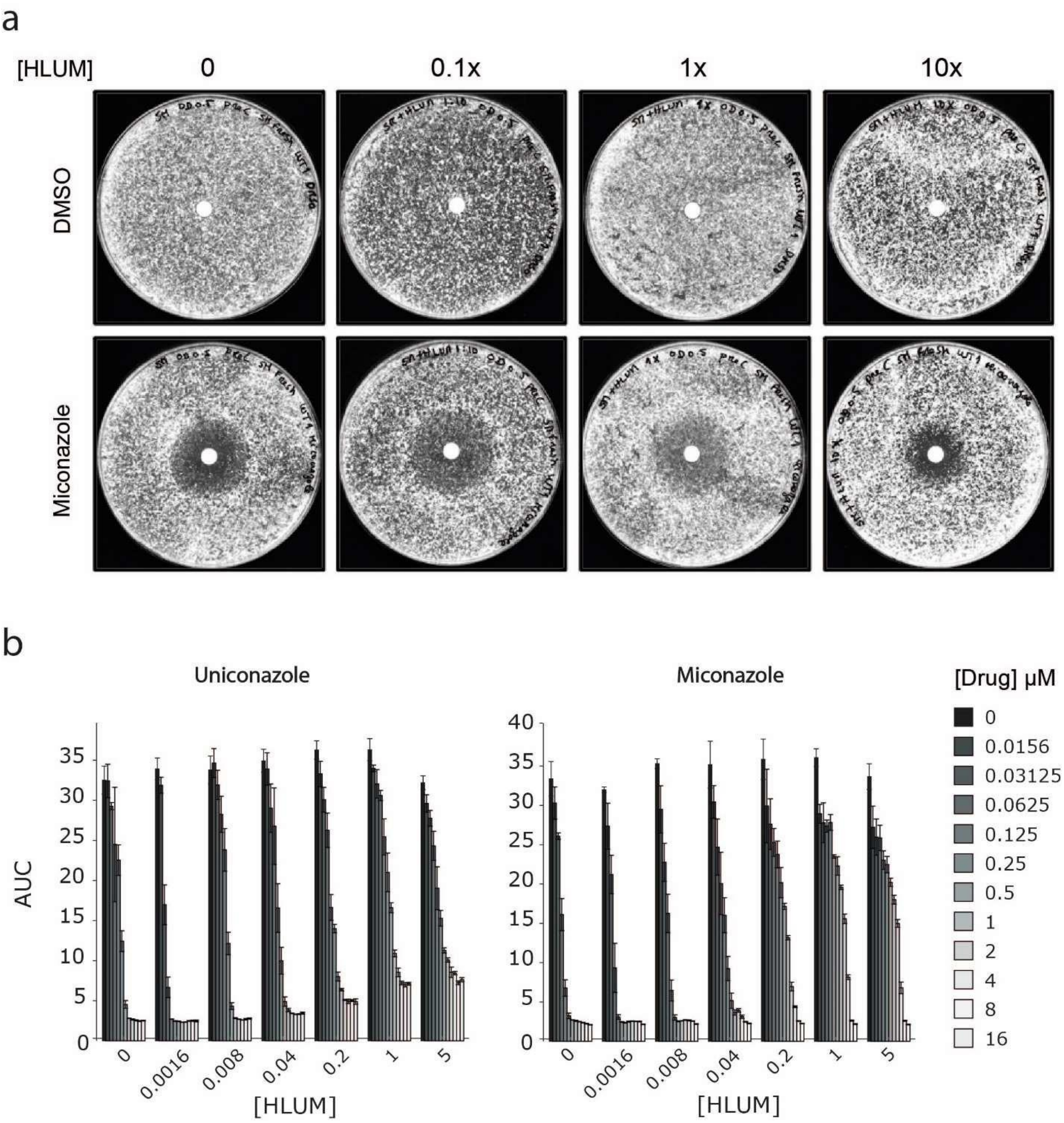

Rich extracellular metabolic environments protect growth of prototrophic yeast against antifungals. (a) Supplementary Fig. S4: Growth response to antifungals, as measured with a disk diffusion assay (DDA) in wild-type colonies in minimal (SM) media supplemented with

increasing concentrations of HLUM, treated with miconazole. Data are n=1 wild-type culture. (b) Fig. 2e: Summary of liquid growth curve data, comparing AUC values when drug concentrations are titered against HLUM concentration.

Specific comments:

5) Line 93 – The authors might consider softening the language in this sentence to reflect that while most microbes have not yet been isolated in monoculture in the lab, it may be readily possible on the correct medium. Indeed, if all that microbes require is metabolites like amino acids it should be feasible to generate media with the correct supplements for most species. 93: Indeed, auxotrophy is one of the main reasons why a large fraction of microorganisms cannot be cultivated in the laboratory outside their community environment.

We completely agree and have revised accordingly.

6) Line 98 – I have trouble following the logic here. It seems that the previous sentences describe why prototrophs should be outcompeted by auxotrophs, but this sentence seems to mix a benefit for auxotrophs (Black Queen) and a benefit of prototrophy (bet hedging). In the next sentence bet hedging is referred to as promoting auxotrophy and I don't understand this rationale. Shouldn't maintaining the ability to metabolize many compounds provide a benefit for prototrophy in variable environments?

The point we tried to make in this section is that our results provide evidence showing that there are different ways on how communities can profit from the presence of auxotrophic cells, particularly by contributing to the metabolic enrichment of the communal metabolic space and conferring increased robustness against a variety of drugs. The Black Queen hypothesis considers the direct metabolic benefits to the auxotroph, bet hedging theories address the advantages of phenotypic diversities in stress situations. Although we do not address the evolution of communities in our study, our observations add to these models and the understanding of auxotrophy-prototrophy interactions. We reveal that cells incur a metabolic cost for obtaining metabolites from producers, which impacts our understanding of prototroph- auxotroph interaction (i.e. models like the Black Queen hypothesis do not consider such costs), and we show that the presence of auxotrophs changes the global metabolic properties of the community, impacting drug resilience.

We hope the revised manuscript reflects this without being confusing. Due to the new structure of the manuscript (to address the reviewer's comment #2), we have separated the evolutionary implications from the discussion about the communal metabolic properties. We discuss the implications for the co-evolution of auxotrophs and prototrophs solely in the discussion, to avoid a confusion with the actual metabolic properties when auxotrophs and prototrophs interact in a community.

7) Line 116 – Auxotrophy might provide a benefit to an auxotrophic cell, but it is not clear to me why it would protect the rest of the community.

Please see above (response #6), the point put to discussion was that prototrophs can exploit an increase in extracellular metabolite concentrations as much as auxotrophs.

8) Line 148 – The description in the text makes it sound like the average number of auxotrophic species across communities, but from the figure I think the percentages actually represent the percent of communities that have more auxotrophs than prototrophs. This needs to be clarified.

We apologize for the lack of clarity, we have now revised the figure and text to be more precise. Indeed, the figure is intended to highlight the percentage of communities where the sum of auxotrophic species abundance exceeds that of the prototrophic species.

9) Line 152 – Are the 40 species all bacteria or a combination of bacteria, yeast and protists?

Thank you for spotting this. The 40 species analysed by Maier et al. are bacteria. We have added this information to the manuscript. We believe it strengthens the generality of findings, that increased growth in the presence of drugs is observed in many prokaryotes and yeast, once they are exposed to more metabolite rich exometabolomes.

10) Line 159 – First it is not clear if this is new data or simply restating data collected in the previous paper.

Please see our response comment #1. The raw data we used for Fig. 1a,b is derived from existing resources, as we believed using public large-scale and community datasets (Earth Microbiome Project [2], Maier et al. [1]) is the least biased way to evaluate the occurrence of auxotrophs in microbial communities and their general response to drugs. All specific data concerning SeMeCos (all other figures except Fig 1a,b) have been generated as part of this submission, and will be made available for further studies in the respective repositories.

11) Second, it needs to be made clear that you are taking the AUC with antibiotics relative to the AUC without antibiotics. Further it would be helpful to know how AUC in the absence of antibiotics compares for prototrophs and auxotrophs. Is the difference in resistance a function of growth rate rather than auxotrophy per se?

We have clarified this now better. AUC values are normalized to the value of 1 for “no growth effect” and we have now mentioned this explicitly in the text. We have added an explicit statement to the revised text and hope it is more clear to the reader.

The differences in drug tolerance are not a function of growth rates, but rather accounted for in the normalized AUC values since growth curves were truncated at the transitions from exponential to stationary phase (see ‘Normalization of growth curves and quantification of growth’ in the Methods section of Maier et al. [1]).

12) Line 165 – Did the auxotrophs actually outgrow the prototrophs are (sic: or) just suffer a lower reduction in growth relative to no antibiotic conditions. This relates to the point above about knowing the relative growth of auxotrophs and prototrophs in the absence of antibiotics.

In the Resource data by Maier et al [1], no absolute data is presented, and hence our re-analysis only indicates that the auxotrophs suffer a lower reduction in growth than the prototrophs. In our yeast experiments conducted with SeMeCos, the auxotrophs outperform the prototrophs in the presence of a large number of drugs, antifungals in particular (Fig. 1e-h, Fig. 4c-e). The main text has been revised for clarity.

13) Line 173 – I think it is more common to use tolerance to refer to the death rate in the presence of antibiotics, and resistance as the ability to grow in the presence of antibiotics. I would recommend at least clearly defining how you are using the term.

The terms tolerance and resistance used throughout the manuscript were based on definitions described in the antifungal context [5], and these may slightly differ to the definition of tolerance and resistance used in studies that have their main focus on bacteria. The reason why definitions diverge has to do with the fact that many antibacterials studied are bactericidal, but many antifungals act mostly as fungistatics.

In order to avoid confusion, we have reduced the use of both terms, in situations where it's not clear if the increased growth is due to resistance or tolerance, or both. Second, we have included a better definition of the two terms, as they are used in our study. The resistance definition we use, is the ability of a strain to grow well at drug concentrations above the MIC. Like others in the fungal field, we measure resistance, which is drug concentration dependent, as the size of the zone of inhibition in disk diffusion assays or as the inhibitory concentration in broth microdilution assays. In the fungal context, tolerance is often defined as the ability of subpopulations of susceptible cells to grow slowly in the drug and is measured as the fraction of growth seen within the zone of inhibition in disk diffusion assays or above the MIC in microbroth dilution assays; it is typically not used in the fungal field to describe dormant or quiescent cells. Another distinction is that for bacteria, tolerance (due to longer lag phase or more quiescence) is measured with bactericidal drugs whereas fungal tolerance is usually measured with (the more commonly used) fungistatic drugs of the azole class. We have also rewritten the text to avoid using these terms as much as possible and referring directly to the growth phenotype observed.

14) Line 194 – I would find it easier to first see a simple bar graph showing the (sic???)

We thank the reviewer for this helpful suggestion. Indeed, the effect is much more visible in the barchart, as shown below. The barplot, now in Fig. 1f, shows the top 42 drugs in the SeMeCo screen that induced the largest change in the auxotroph-prototroph composition based on their Z-score. The sunburst plot categorises these drugs based on their class or primary mode of action, revealing that it is dominated by antimicrobial compounds and particularly of the azole class. However, we also present the PCA (Fig. 1e) alongside since it gives insight into the

hierarchical clustering and contribution of each subpopulation to each principal component which we use to designate drugs as hits.

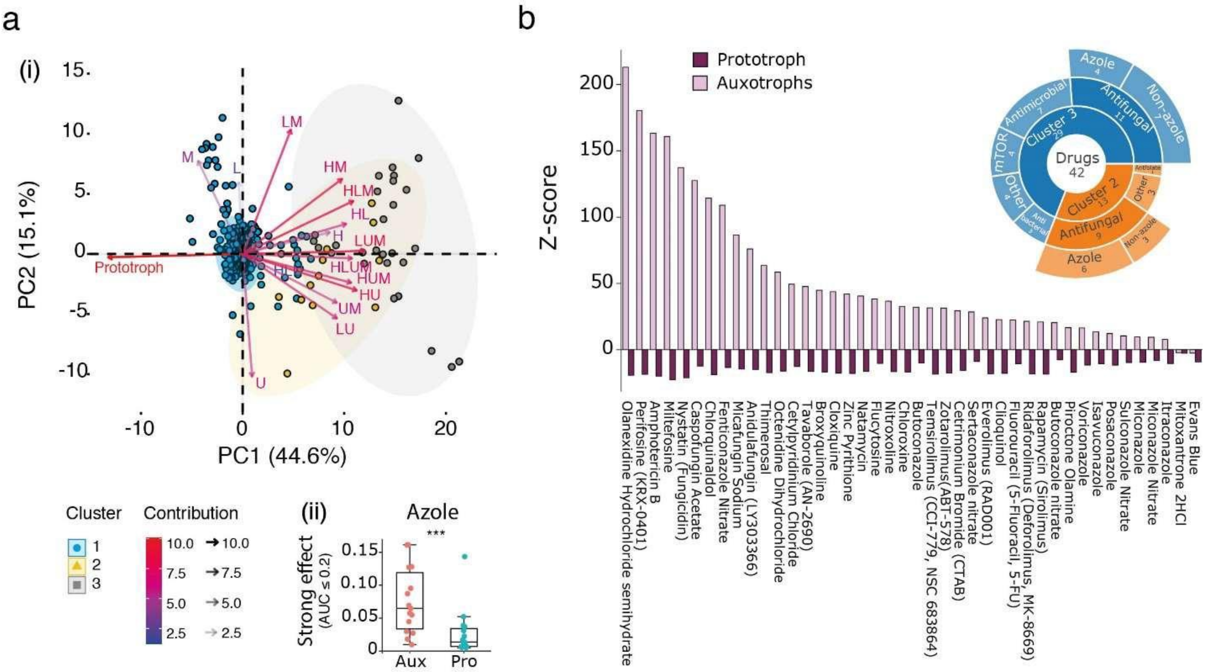

Frequency of auxotrophic populations are increased in the presence of drugs in synthetic SeMeCo communities. Fig. 1(e): (i) SeMeCo colonies were exposed to 900 compounds taken from an FDA approved compound library based on drug categories identified in Fig. 1c. Principal component analysis (PCA) of the Z-scores assessing the impact of drug exposure to auxotrophic composition, available in Supplementary File 3. Several drugs increased the auxotroph composition in the SeMeCo which are a key driver of variance (PC1 and PC2, highlighted by arrows). Two distinct clusters (cluster 2 (yellow and cluster 3 (grey) were identified via hierarchical clustering. Azoles comprise a large proportion of drugs found in clusters 2 or 3 (10/42). (ii) Subset of gut microbiome AUC values for potent azoles found in cluster 2 or 3 where AUC < 0.2 (strong drug effect), also revealed a significant increase in growth of auxotrophs compared to prototrophs. Subset details and P-values are available in Supplementary File 4, n = number of drug-microbe pairs. Statistical significance was determined using Wilcoxon Rank Sum test, *** = P<0.0005. Fig. 1(f): Effect of 42 drugs on the auxotrophic/prototrophic composition of SeMeCos. The 42 drugs were selected from a total of 900 drugs that had the strongest impact on the SeMeCo composition according to the PCA (e). Insert (sunburst plot) classifies these drugs based on their target and known activity.

15) Line 196 – How were the clusters determined? The figure legend says based on correlation, but correlation of what?

We apologise that this was not explicitly mentioned. The clusters were derived from hierarchical clustering on the principal components, which we believe is appropriate as we wanted to identify groups of similar observations in the screen, given certain drugs are known to belong to the same class. As stated in the previous comment (response #14), we have now added the summary barplot and sunburst plot.

16) Line 198 – It seems possible that the change in ratio of auxotrophs could also be driven by antibiotics altering the fidelity with which plasmids are transmitted to offspring. How have you ruled out this possibility?

We have addressed the question by the reviewer in two experiments. In the first experiment, we transformed WT cells with a plasmid carrying the antibiotic nourseothricin (centromeric plasmid similar to the ones used in the SeMeCo system) via the NatMX6 cassette (MitoLoc, Addgene #58980, [6]). Compared to WT (no plasmid) and SeMeCo (4 plasmid) we did not observe a different growth response to uniconazole on both SM and SM+HLUM media, regardless of whether plasmid segregation was permitted or prevented in the presence or absence of nourseothricin (MitoLoc -NAT vs MitoLoc +NAT). In the second experiment, we have repeated the drug tolerance experiment with auxotrophic strains in which three of the four markers were genomically integrated, and obtained a similar result. We are therefore confident that these results rule out the influence of the azole-class antifungals, used to validate our observations, on plasmid segregation and the emergence of auxotrophs.

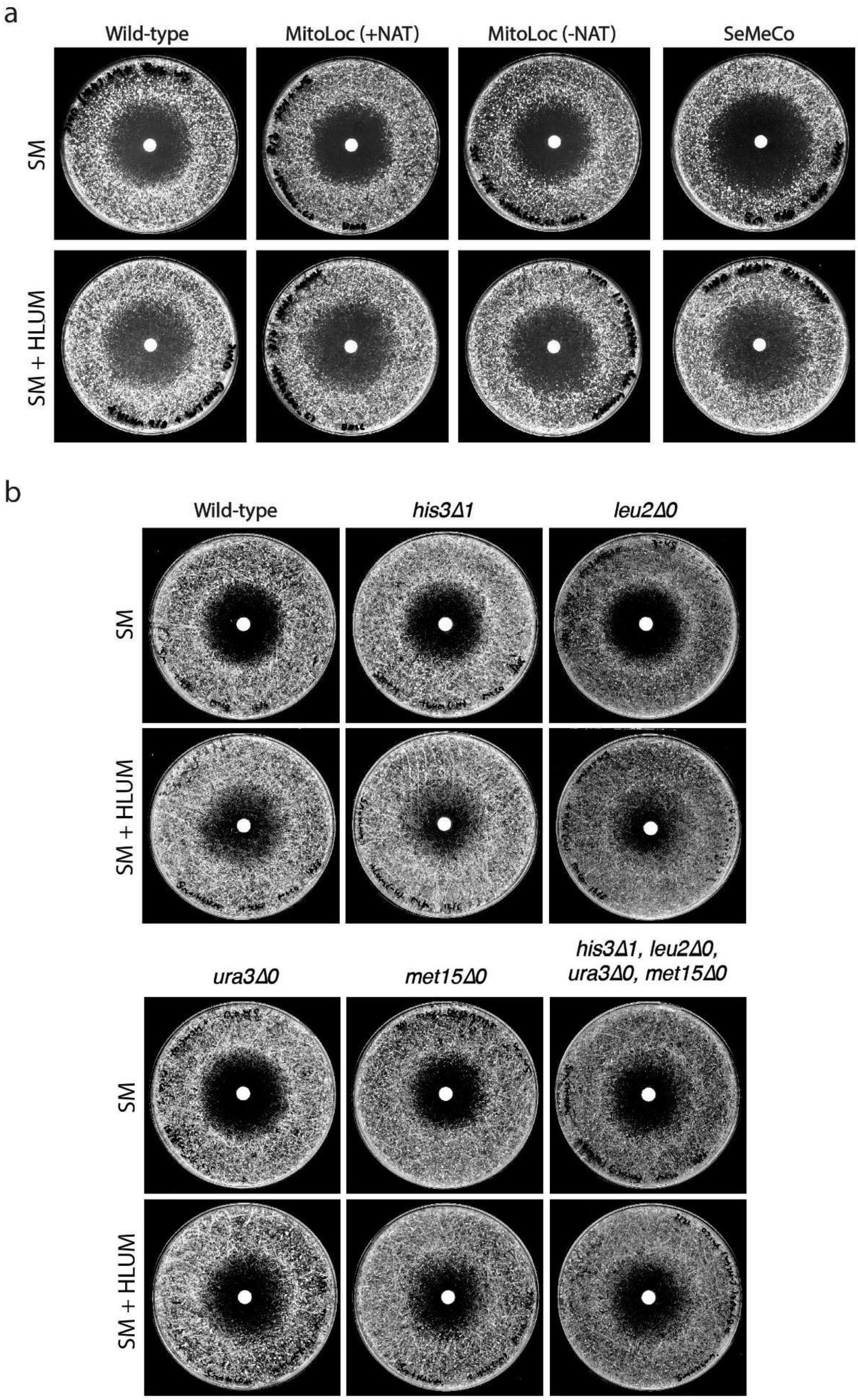
a
Wild-type
MitoLoc (+NAT)
MitoLoc (-NAT)
SeMeCo
SM
SM + HLUM
b
Wild-type
his3Δ1
leu2Δ0
SM
SM + HLUM
ura3Δ0
met15Δ0
his3Δ1, leu2Δ0, ura3Δ0, met15Δ0
SM
SM + HLUM

Drug response of SeMeCos is similar to that of cells containing an antibiotic plasmid selection marker or genomically reconstituted auxotrophs. Supplementary Fig. S2: (a) Drug disk diffusion assays of wild-type (WT) strains without plasmid or transformed with MitoLOC plasmid [6] that encode for nourseothricin resistance (NAT) compared against the SeMeCo strain which carries 4 plasmids when exposed to uniconazole. (b) Disk diffusion assays of WT prototrophic strain compared against singly auxotrophic strains, where the remaining three auxotrophies were repaired by genomic integration, and a quadruple auxotrophic parental strain when exposed to miconazole. SM indicated minimal media condition versus SM+HLUM whereby minimal media was supplemented with the 4 amino acids. Singly auxotrophic strains were supplemented with the respective amino acid to ensure their growth in the absence of genomic or plasmid complementation.

17) Line 208 – By “vehicle-alone” do you mean the system in the absence of antibiotics?

Vehicle-control refers to treatment with just DMSO. The same volume of DMSO and drug is added in all experiments. We have clarified this in the text, and apologize that we had missed that in our first submission.

18) Line 211 – Maximizing community growth rate assumes that auxotroph and prototroph pairs have been under group selection that has led to the evolution of cooperation. I don’t see that this selection was actually imposed in the system, and therefore the assumption does not seem justified. Rather I would expect the auxotroph and prototroph to still be optimizing individual interests.

Please also see our response to comment #2. We actually completely agree with the reviewer and apologize if our choice of language seems to have suggested otherwise. SeMeCos survive by exchanging metabolites, but all underlying mechanisms that facilitate their proliferation are explained by the metabolic properties of the wild-type cell. The yeast cell senses and consumes extracellular metabolites, as the experimental evidence shows, as well as the structure of the metabolic network that we model in the FBA. Hence, we entirely agree with the reviewer, and did not aim to imply otherwise, that neither a priori co-evolution of auxotrophs and prototrophs is directly studied in our manuscript, nor is coevolution necessary to explain our results. Our FBA modeling is based on the experimentally confirmed property of yeast cells that inhibit their metabolic pathways once metabolites become available in the extracellular environment, and the model predicts flux distributions over the topological structure of the metabolic network, which is mostly driven by the chemistry of the metabolites. As discussed in the above mentioned comments, the objective function in the FBA model is accounting for this property, and addresses the metabolic interactions that reflect the flux distribution in our experimental model, the SeMeCos, in which auxotrophs obtain essential metabolites from the prototrophs for growth.

19) Line 237 – It is really interesting that different metabolites are measured in the SeMeCo relative to a wildtype. How is the impact that plasmids have in SeMeCo differentiated from the impact of auxotroph presence? Also what was the abundance of auxotrophs in these experiments? To the extent that auxotrophy alters metabolism (rather than plasmid presence), I wonder if the alteration is due to the loss of a gene or due to the cell being limited by an amino

acid (for example if SeMeCo were grown in media supplied with the amino acids would you see the same changes in metabolism?).

We are glad that the reviewer found this as interesting as we also questioned whether it is the auxotrophy per se or interactions with prototrophs induced by the auxotrophy in the SeMeCo that leads to metabolite overflow and a rich exometabolome. We addressed this point extensively in our previous work [4,7–9]. The majority of the physiological changes comes from the transition from uptake to self-synthesis of a particular metabolite (activation/suppression of the involved metabolic pathways). We can explain a large fraction of the transcriptional changes by the necessary reconfiguration of the metabolic network [7]. Under the conditions we study, cells are not nutrient restricted and show no signs of a starvation response.

We have now added to the manuscript the percentage of auxotrophs vs prototrophs in the communities for which we analysed the intracellular and extracellular metabolite profiles (Fig 2c). In addressing the comment on whether SeMeCos are limited for the amino acids they share, our data shows that these metabolites are abundant in the intracellular and/or extracellular space (Fig. 2c (i)). We also show that the general increase of amino acids and uracil in both fractions occur in SeMeCos where ~75% of the cells were auxotrophic for H, L, U and/or M (Fig. 2c (ii)).

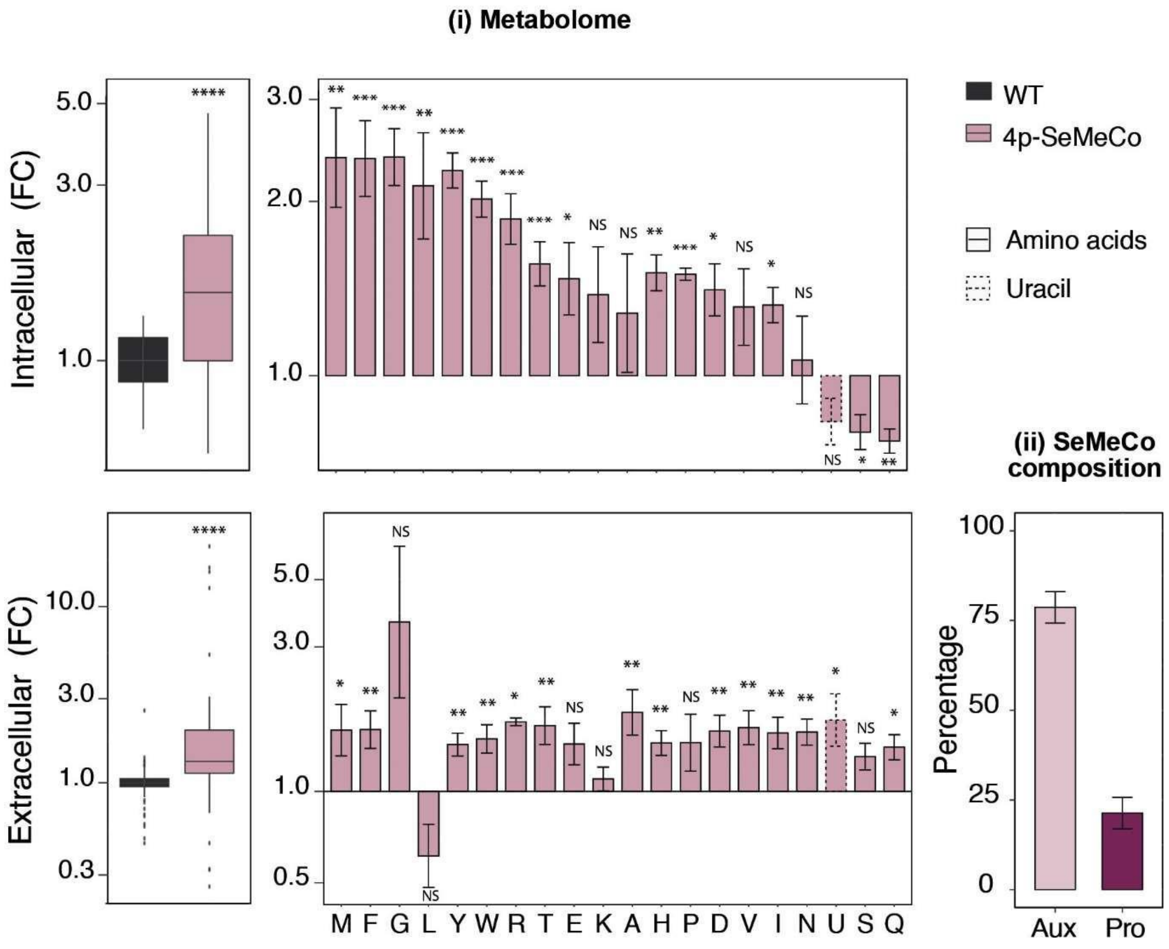

Auxotrophs promote a rich metabolic environment in SeMeCo communities. Fig. 2: (c) (i) Quantification of intracellular and extracellular metabolites (amino acids and uracil), by LC-MS, in exponential growing (24h post-inoculation) SeMeCos compared to wild-type cultures in synthetic minimal (SM) medium. Concentration of each metabolite was normalised by biomass, determined by OD_{600}. Box plots represent median (50% quantile (middle line) and lower and upper quantiles (lower (25% quantile) and upper (75% quantile) respectively) of pooled metabolite levels, and bar plots represent individual metabolite levels as mean±SEM of 4 independent cultures of each strain, performed across 2 independent experiments (total n = 8 cultures/strain). Statistical significance was determined using Wilcoxon Rank Sum test, * = P<0.05; ** = P<0.005; *** = P<0.0005; **** = P<0.00005. (ii) Frequency of auxotrophs and prototrophs in the SeMeCos analysed in (i), calculated by spotting colonies onto selective media (see Material & Methods). Bar plots represent the relative frequency (%) of auxotrophs and prototrophs in SeMeCos as mean±SEM of 3 independent cultures, performed across 2 independent experiments (total n = 6 cultures).

20) Line 270 – I don't think that this data demonstrates that prototrophs were responding to the presence of the auxotrophs. It simply indicates that there was a difference in expression between prototrophs and auxotrophs.

We have addressed this point in combination with the following point.

21) Line 281 – I am confused about the comparison that is being made here. Is expression being compared between prototrophs grown with auxotrophs and prototrophs grown with wildtype? Or are prototrophs being compared against wildtype? What are the auxotrophs being compared against? I find the text and figure 3 highly confusing.

We apologize if our explanation of these experiments was not clear and that our description of them has been confusing. Our analysis indeed contained both comparisons: one between the proteome of prototrophs and auxotrophs growing as part of a SeMeCo, and the other between two different prototrophic proteomes that differ in the presence or absence of co-growing auxotrophs.

We have re-written this part in the main text and heavily revised Fig. 3 in order to avoid this confusion. We agree that this part can be a bit difficult to describe, but we are very excited by the result that the proteome of prototrophs is different when they grow alongside the auxotrophs inside a SeMeCo, compared to when they grow among other prototrophic cells in wild-type colonies.

22) Further comments:
To reiterate the comment above I do not think that the observation of metabolic shifts in SeMeCo validates the modelling assumption that cells have evolved to cooperate. Rather I think the model is generating a similar observation of metabolite exchange for the wrong reasons.

Please see the response to comment #2; we have not attempted to model the co-evolution of prototrophs and auxotrophs; the FBA model addresses the flux changes caused by the exchange of amino acids in SeMeCos, in which strains possess four auxotrophies in all 16 possible combinations and grow as they cross feed each other. So the experimental model community reflects the actual state of a community in which auxotrophs and prototrophs share a common metabolic space (in an isogenic setting), but neither its evolutionary trajectory nor the basis why this property evolved. In order to avoid confusion, we have moved all speculations about the evolutionary consequences to the discussion.

23) How is the impact that plasmids have in SeMeCo differentiated from the impact of auxotroph presence?

To the extent that auxotrophy alters metabolism (rather than plasmid presence), I wonder if the alteration is due to the loss of a gene or due to the cell being limited by an amino acid (for example if SeMeCo were grown in media supplied with the amino acids would you see the same changes in metabolism?).

Please see above, comment to Line 237 (response #19); we have specifically focused on this research question in our previous work [7]. The main physiological changes come from the change of synthesis to uptake of the studied metabolites, which changes the flux distribution in the metabolic network. A majority of transcriptional changes correlated with the changes in flux distribution, under the growth conditions used, we detect no signs of nutrient starvation (proteome, growth rate).

24) Also what was the abundance of auxotrophs in these experiments?

We thank the reviewer for this point and have also addressed this above (response#19). The SeMeCo composition is ~75% auxotrophic. This data has now been included in the figure (Fig. 2c (ii)).

25) Line 294 – It seems like this sentence is implying that auxotrophs make the prototroph grow faster than it would in the absence of the auxotrophs, but the experiment that follows is instead testing the growth rate of the prototroph relative to the auxotroph.

We apologize if the sentence was equivocal. The reviewer is correct, the experiment probes for relative rather than absolute growth rate differences (i.e. its a competitive growth assay). We have corrected it in the manuscript.

26) Line 323 – Was cell size controlled for? If auxotrophic cells are smaller than they would fluoresce less but not because of a change in pumping activity.

Using our SeMeCo screening data, we found that azole-class drugs, used to validate our observations, do not alter the mean cell size when compared to DMSO-treated in our experiments

(Supplementary Fig. S9), however, as expected, some other drugs do alter cell size to differing degrees which might be dependent on their specific mode of action. We thank the reviewer for this suggestion, we have added a sentence explaining that at least for some drugs, altered cell size needs to be taken into account when considering the underlying mechanisms for a change in drug resilience and or transport.

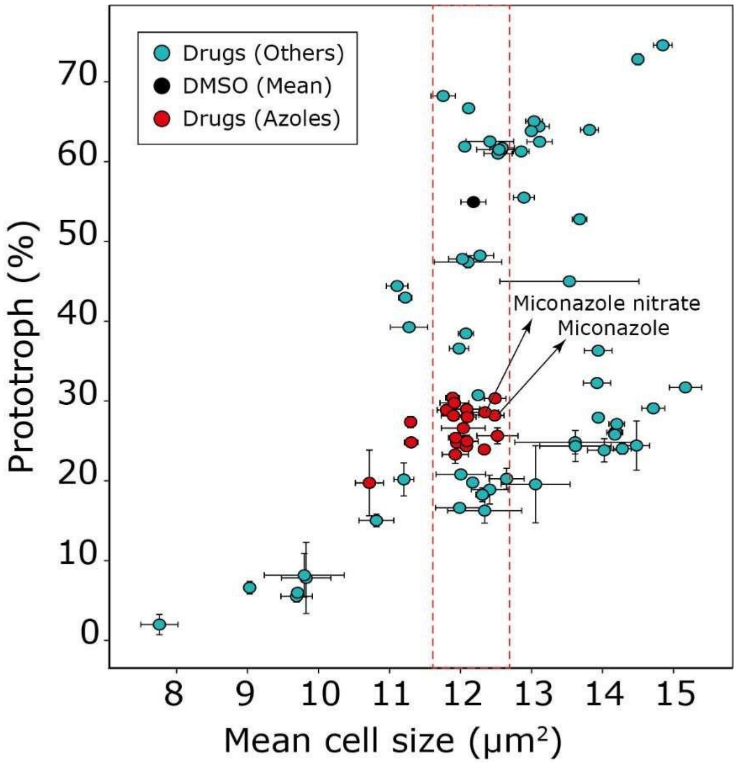

Supplementary Fig. S9: Azole treatment changes the proportion of auxotrophic subpopulations in SeMeCo independent of cell size. Summary of the changes in mean cell size against SeMeCo composition change as measured in the drug screen for the 42 screen hits. Cell size was defined as the pixel area covered by a cell captured via high-throughput microscopy. DMSO (mean) indicates the global mean value from all DMSO treated wells. Drugs (Azoles) indicate azoles within the compound collection, dotted lines indicate range of cell size changes for azole treated cells. Error bars indicate standard deviation of prototroph percentage or cell size from 3 biological replicates.

27) Line 336 – It isn't clear to me here or in the supplement how concentration was determined. Were the number of cells in each sample accounted for?

We apologize that this part was missing in the methods section. Following overnight culture in the presence of 10 µM uniconazole, an equal number of cells were sorted, gated for CFP- (auxotrophic) and CFP+(prototrophic) populations, harvested, and then taken for metabolite extraction followed by LC-MS. The same concentration of miconazole or uniconazole at 10 µM was used throughout all experiments in liquid culture with the exception of Fig. 2e.

28) Line 342 – I don't understand, didn't you just show that adding amino acids increases the resistance in yeast? Why are you arguing here that the difference is resistance is driven by genotype rather than by media?

We felt it is important to reiterate the switch from self-synthesizing a metabolite to uptake which induces drug resilience. The lack of the gene (genotype) can induce these shifts, but also, the non-genetic mechanisms, such as the feedback inhibition that act in the prototrophs. The metabolic consequences are similar, and drug resilience is affected in either case.

29) Line 394 – This in essence just sets up a mutualism where each genotype is providing a costly benefit rather than just one of the genotypes providing a costly benefit. Mutualisms are still subject to exploitation. A pair of strains that exchange metabolites might grow faster than a pair of strains that don't exchange metabolites. However if you introduced a cheater into the original pair that could use the metabolites and not provide anything in return it should have a growth advantage and be able to outcompete a cooperating strain. To avoid this you would need a mechanism such as spatial structure that generates a correlation between the amount a genotype cooperates and how much it gets in return.

We agree with the reviewer, our initial statement was too strong, and we have reworded it. Importantly, however, our data show that each auxotrophs return other metabolites to the extracellular space. This is due to the topological structure of the metabolic network, as our FBA analysis shows, the auxotrophs also incur a metabolic return cost for uptaking the essential metabolite. This is something important and has not been appreciated before. That certainly does not mean exploitation is completely prevented, but it sheds a new light on the cost-benefit of being an auxotroph versus a prototroph.

30) Line 410 – What is the data supporting this statement? Is it increased uptake or increased excretion that leads to the effect?

Both, the increased uptake of the essential metabolite, which results in the increased overflow of several other (non-essential) metabolites that are coupled in flux. We apologize if this was confusing, and have reworded the paragraph.

31) Line 414 – It might be interesting to contrast this speculation with the observation in (https://doi.org/10.1371/journal.ppat.1008700).

We thank the reviewer for bringing this study to our attention. Although Adamovicz et al. did observe that obligate cross-feeding co-cultures that depend on metabolic interactions for survival have a reduced rate in the occurrence of resistance mutations, this does not preclude our argument, but rather reinforces it. A fundamental feature of tolerance is slowed or delayed growth, which confers time for antimicrobial resistance to emerge. In short, resistance emerges nonetheless in obligate cocultures, just at a slower rate. We have now formulated this argument and contrasted this observation in the main manuscript as suggested by the reviewer.

32) Line 619 – I don't understand how information on gene essentiality was used. Was this used simply to improve the S. cerevisiae model that was used? Counting a gene as essential because it reduces growth by 10% seems like quite an extreme cutoff. Also S. cerevisiae should be italicized in line620.

We apologize that the different use of the terminology was potentially confusing. The auxotrophic markers used in the study (his3Δ, leu2Δ, ura3Δ and met15Δ) are very well experimentally characterised, therefore, we have completely removed other predictions from the study, as they were not necessary.

Just to explain what we had referred to in the initial document: "Essential gene" in the context of a genome scale metabolic model typically refers to a gene associated with metabolic reactions required for the model to generate biomass (i.e. that all life essential metabolites are produced at sufficient rate). A gene is considered essential for the metabolic model, if its knockout reduces the growth rate (i.e. biomass formation) by more than a set limit (we use >90%), compared to the wild-type. We initially used the genome-scale metabolic model to predict the auxotrophic marker genes; all four his3Δ, leu2Δ, ura3Δ and met15Δ were correctly predicted by the FBA.

Reviewer #2 (Remarks to the Author):

1) The authors explore the differential response of auxotrophs and prototrophs under the exposures to various drugs by measuring the growth of each type in communities. First, they show that auxotrophs are enriched in many host-associated communities and measure the enrichment of auxotrophs during growth under various drugs. Building on their previously established SeMeCos system which reaches a stable co-existence of autotrophs and phototrophs, they also measure the enrichment of auxotrophs under various drugs in this system. They find that, similarly to the results in natural communities, auxotrophs grow better, especially under drugs that have a strong effect in reducing the growth such as azole antifungals and statins. The better growth under the drug means that the auxotrophs are more resistant (see comment 1 below). The metabolome data shows that the presence of auxotrophs results in enrichment of the extracellular environment by increased concentrations of amino acids. The authors then attribute the higher resistance of auxotrophs under drugs to the increased concentrations of amino acids. Accordingly, they show that both auxotrophs in SeMeCo and wt communities are also more resistant to azoles when grown in the presence of HLUM supplementation. This suggests that the metabolism involved in the uptake of amino acids results in a higher resistance level. In order to understand this point, the authors use a dye that enables them to measure the level of efflux on single cells and show that auxotrophs have a lower level of the dye. They then directly measure the intracellular level of azole and show that it is lowered in auxotroph, explaining their higher resistance.The results are interesting and add to the body of work showing how microbial communities can lead to different antibiotic susceptibility profiles when grown together. The metabolic effect that they uncover should be interesting to a broad community.

We thank the reviewer for their thorough and positive report.

2) Main comment 1: the authors term the effect that they measure "tolerance" while they measure the impairment in growth. They are described as "tolerant" strains that grow better under the drug.

However, tolerance is typically used for cidal treatments. A strain that grows better at the same drug concentration is simply considered as more resistant. Here all data seem to show that in the conditions in which the experiment are performed, the MIC of auxotrophs is higher. The rest of the findings agree well with this view: resistance mechanisms (higher efflux) do lower the effective concentration of the drug (which is reflected in the higher MIC). Replacing all instances of "tolerance" by "resistance" would make this clearer. Otherwise, the authors would need to show that the MIC does not change for example for the wt with and without HLUM supplementation. According to the DDA, the resistance level has increased, showing resistance. The findings are no less interesting if amino acid metabolism as seen in auxotrophs is shown to lead to resistance but then the work should be put in the context of induced resistance.

The reviewer refers to a debate in the field that is frequently shrouded in confusion due to the definition of resistance and tolerance that are subtly different between bacterial and fungal fields (also see reviewer #1, response #13). These differences have a scientific origin: studies on bacterial tolerance have been conducted primarily with bactericidal drugs, while fungal tolerance has been studied with fungistatic drugs. Fungal tolerance typically circumscribes slow growth in drug concentrations beyond the MIC over time, while bacterial tolerance is often used to describe longer lag phases or more of a quiescent state during drug exposure. We apologize that we have not explained this debate in our previous manuscript version, and try to avoid any confusion now by providing the specific definition on which basis we work. We have also rephrased in many places to remove the usage of either tolerance or resistance and refer directly to the observed growth phenotype.

We found that the best way to illustrate the difference between tolerance and resistance (in fungal cells) is to consider the disk diffusion assay. Cells which grow within the zone of inhibition are defined as tolerant, whereas the diameter of the zone indicates the degree of resistance (both assays are also outlined in the susceptibility test interpretive criteria (STIC) as stipulated by both the FDA and EMA to define fungal resistance [10,11]). A more resistant strain would have a smaller zone of inhibition, whereas a tolerant strain would be able to form subpopulations that grow within the zone regardless of its size. It follows that a high or low MIC is not indicative of greater/less tolerance, it is more the fraction of growth within the zone in the presence of a drug that it would have achieved relative to no drug. Indeed although there are slight changes to the halo size indicating a change in resistance/drug sensitivity the dominant effect of supplementation in yeast communities is one of increased tolerance.

To avoid confusion regarding the use of terminology, we have put greater thought into the specific instances that are appropriate for their use in the revision and hope this is now clearer both to the reviewer and general readership.

3) Minor comments:

It is difficult to compare the results obtained on the microbiome communities and the SeMeCos as they are presented in very different formats. It would be helpful to translate the data obtained on the communities (AUC) into the simple parameters used for the SeMeCos (for example the enrichment of auxotrophs as shown in Fig.1). Many of the significance tests turn out to be non-significant in Supplementary Fig. S1. Please mention how the significance was made to take into account the multiple hypotheses tested.

We agree that although the reviewer's suggestion would indeed ease comparisons between the two sets of data, it is difficult to do so as they measure fundamentally different aspects of the drug response. For the microbiome communities, the AUC reflects the change in growth of auxotrophs and prototrophs in the presence of a drug. Conversely, the SeMeCo drug screen measures the composition of the community and how this changes in the presence of a drug. One is a growth parameter attributed to specific subpopulations whereas the other is a drug response parameter reflecting a community response. We generally find that drugs that promote better growth (higher AUC) in auxotrophs tend to also promote a more auxotrophic composition in the SeMeCo screen (e.g. Azoles, Fig. 1e(ii)), and are therefore comparable.

Regarding significance testing, one-sided Wilcoxon rank sum test was used to calculate p-values and the False Discovery Rate (FDR) method was applied to correct for multiple hypothesis testing. We have added this information to Supplementary File 4 for detailed reporting of corrected and uncorrected p-values. Indeed not all drug categories showed significant differences between auxotrophs and prototrophs, this follows the expectation that not every single drug is better tolerated by an auxotroph. Drug categories that did not show significant changes from Fig. 1c are shown in Supplementary Fig. S1. Of note, we did not find a single drug category in which the prototrophs did better than the auxotrophs.

<u>Reviewer #3 (Remarks to the Author):</u>

1) Microbial communities display a variety of emergent properties not present in individual species, including robustness to challenge by antimicrobials. Communities are often supported by metabolite exchange between individual species, and auxotrophs (species that lack essential metabolic pathways) have been found in a wide range of microbiomes. The mechanisms that allow auxotrophs to persist, despite their reliance on other community members for essential nutrients, remain unclear. Mysteries include why "cheating" by auxotrophs does not tend to destabilize microbial communities and why prototrophs (which can downregulate biosynthetic pathways through feedback mechanisms) coexist with auxotrophs rather than outcompeting them. In this article the authors show that auxotrophic species are particularly frequent in certain kinds of microbial communities and are more drug resistant than their prototrophic counterparts. They use a model isogenic auxotrophic community of S. cerevisiae yeast (SeMeCo) to experimentally validate the increased drug resistance of auxotrophs. Metabolic reconfiguration by auxotrophs results in system wide changes in flux that alter the intracellular metabolome as well as metabolites exported leading to an enriched communal environment. This enriched environment produces a reciprocal response from prototrophs. Increased efflux and the enriched environment lead to stronger drug tolerance. Overall the authors use the SeMeCo community to good advantage, in combination with metabolic modeling, to explore why auxotrophs confer

antimicrobial resistance in microbial communities. I recommend that the following comments be addressed before publication:

We thank the reviewer for the positive comments and for recommending our manuscript for publication. We are especially pleased that the reviewer highlights the link to tolerance is not to auxotrophy per se, but more to the metabolic changes that occur upon auxotroph-prototroph interaction that also enhances drug export. We note that the issue of definitions regarding tolerance and resistance has again been raised, and kindly refer to the responses made to the above reviewers for further clarification (Reviewer #1: comment #13, Reviewer #2: comment #2).

Major Comments:

2) Please provide more details on how the auxotrophies were calculated, beyond referring to Reference 38.

We thank the reviewer for pointing this out and apologise that it was not clear in our first submission. We have now revised the method section and described in more detail how the auxotrophies were calculated, by using the auxotrophy predictor by Machado et al [3]. In brief, in order to calculate auxotrophies, genome-scale models were first used to calculate the auxotrophies of all reference species. Then, with 100 stochastic character mapping, posterior probabilities of the auxotrophic ancestral state were calculated using functions from the phytools R package [12].

3) I interpret Figure 1A as all communities examined have auxotrophies, but some have more that prototrophs. Is this correct? Or is there a "0" location on the plot? Do any communities contain no auxotrophs?

The reviewer's interpretation of the figure is correct. There is no location "0" on the plot because the x-axis is log-scaled and log(0) is undefined. Only 6 out of 12,538 communities in the dataset contained zero amino acid auxotrophs, while only 1 out of 12,538 communities in which we didn't detect a fully amino acid prototroph. We have added this information to the main text.

4) Can you further discuss auxotrophies that appear to be the exception, for example leucine? Also histidine (lines 266-268) Also in Figure 2 why do certain amino acids increase and not others, is it related in any clear way to the different auxotrophies?

The reviewer refers to i) the metabolome data (where leucine levels decrease while many other metabolites increase), and to ii) the proteome data (where HIS3p was below detection limit.) To answer the first point i) the concentration of a metabolite outside is determined by its export rate by cells, and the import rate by other cells. Intuitively (i.e. without the metabolic reconfigurations we detect as part of our study), one might have expected that the presence of auxotrophs would reduce the concentration of a metabolite that they require, outside the cell, and this is what we detect for leucine. This indicates that, compared to wild type cells, the cells that produce leucine (12 of the 16 genotypes are leucine prototrophs) in aggregate produce less

leucine, as the cells in a wild-type community. However in most other cases, we observe an increase in the metabolite concentration. This indicates that cells that interact in a SeMeCo, produce more of these metabolites, compared to a wild-type community. An explanation has been added to the main text.

ii) The proteomic analysis: Our LC-SWATH-MS method detects about 1,500 of the 4,000-5,000 proteins that are expressed in a typical yeast cell. This is the state of the art of high-throughput proteomic technology [13]). Leu2p, Ura3p, and Met15p are highly expressed in cells, and fall in the group of detected proteins, while His3p falls into the group of lower abundant proteins that is not detected. Hence, the lack of His3p from the proteomic results is purely for technical reasons. As the quantification of Leu2p, Ura3p, and Met15p has been used only as an extra control to independently show the FACS sorting efficiency, the lack of His3p from the proteomes has no influence on the conclusions. We have revised the paragraph for clarity.

5) Throughout the authors draw comparisons between single human gut microbiome species tested individually and the SeMeCo community. Is this reasonable?

We believe the SeMeCo models are indeed excellent to study prototroph-auxotroph interactions, in particular, because our study required an isogenic system to efficiently study the metabolic changes an auxotroph undergoes, in comparison to its community members. Our results show that there are many parallels between the auxotrophy-prototrophy interactions in microbial communities and the laboratory model, the most important one being the increased drug resistance of the auxotrophs in both cases.

However, we agree with the reviewer's point that SeMeCos might not reproduce other properties of communal cells. We have added a discussion point, that we use SeMeCos specifically to model auxotroph-prototroph interactions.

6) Is there any reason to expect that yeast auxotrophs (which have not evolved to have these auxotrophies in specific communities) will behave differently than naturally occurring auxotrophs? This is worth discussing in light of generalizing the paper's results. Do you expect the same "nutrient overflow" and metabolic enrichment for naturally occurring auxotrophs?

Undoubtedly, 'evolved' auxotrophs will carry secondary adaptations. However, we know that cells that are members of natural communities overflow large amounts of metabolites. An example is a community of lactic acid bacteria and yeast that we studied previously [14]. Indeed, auxotrophs possess the same basic metabolic network structure as prototrophs, and the interconnectivity in this metabolic network explains the increased overflow of a broad range of unrelated metabolites, when cells shift from amino acid self-synthesis to uptake. Indeed, we see that in the presence of the metabolite outside of cells, wild-type cells uptake metabolites like the auxotrophs [4], and throughout our experiments, we see that their metabolism is reconfigured accordingly. From this situation we derive that the ability to uptake metabolites for efficient use of the exometabolome is a property of the prototroph, and hence needs no adaptation to come into effect in the first place. Indeed, the discovery of the increased drug tolerance was made in the 'evolved' auxotrophs (i.e.

genetic auxotrophs) and was replicated in the ‘induced’ auxotrophs. This is discussed in the main text.

7) Would a spent media experiment, or similar, verify that it is the metabolically enriched environment that leads prototrophs towards more uptake and hence enhanced drug resistance?

This is a very good suggestion, and addressed by the experiments that we have done to reply to Reviewer #1, comment #4 for questions (Supplementary Figure S4). Wild-type cells increase tolerance and resistance once they are supplied with H, L, U, M. Please see our reply on pages 3-4, which upon further investigation has strengthened the observations and conclusions of this study.

8) Do the tolerance experiments (Figure 3) demonstrate that it is increased uptake that leads prototrophs towards drug tolerance? Could it simply be growth rate differences on supplemental amino acids or other metabolic changes? Is there a way to directly show that increasing uptake leads to drug tolerance, especially in light of the fact that this seems to conflict with the increased export mechanism in auxotrophs?

Again, very good points from the reviewer. We have addressed the concern regarding the increased uptake that leads to prototrophs becoming more drug tolerant in the previous point and in more detail in response to reviewer #1 comment #4. Although increased supplementation does increase drug tolerance, this is independent of its growth promoting effects, as AUC values did not significantly change in the untreated controls. We have highlighted this point in the manuscript.

Another way we have shown this was by performing DDAs using prototrophic wild-type cells at different growth phases, reflecting the growth cells at different rates, specifically in early exponential phase and late exponential phase, as measured by OD_{600} of 0.5 or 1.5 respectively, grown in minimal media (SM) or media supplemented media with HLUM (SM+HLUM). We observed that the protective effect of a richer metabolic environment (SM+HLUM) on growth of cells under drug treatment was independent of growth phase. These observations have now been included in Supplementary Fig. S4.

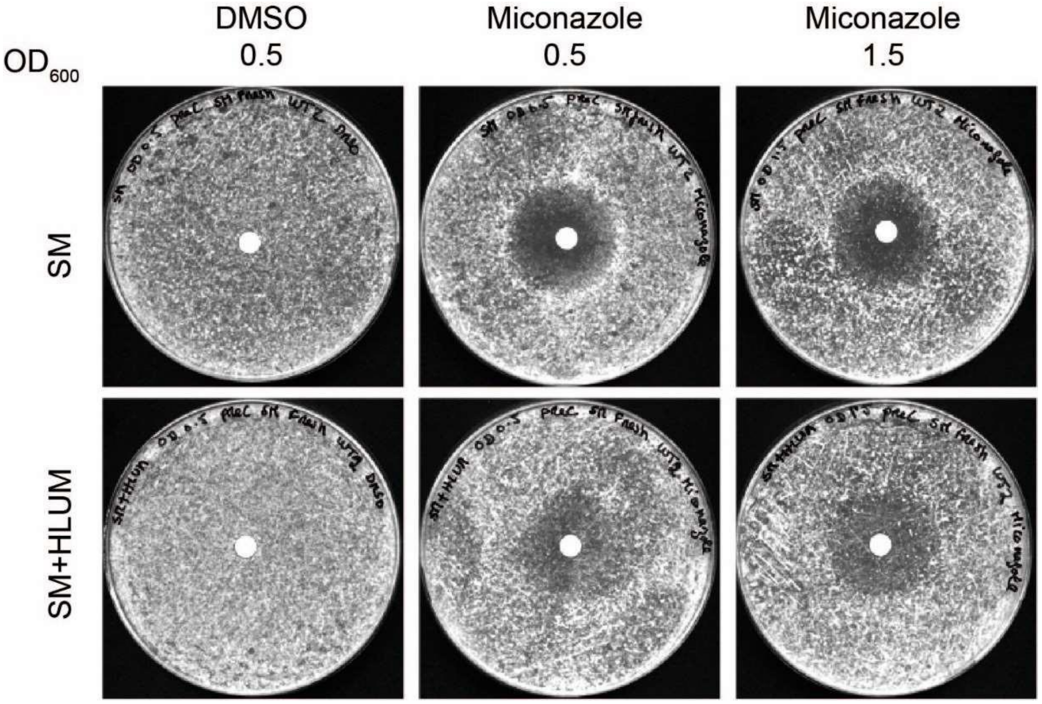

Rich extracellular metabolic environments protect growth of prototrophic yeast against antifungals independently of growth phase. Supplementary Fig. S4b: Growth response to antifungals, as measured with a disk diffusion assay (DDA) in wild-type colonies in minimal (SM) media supplemented at different growth phases: initial exponential phase (OD_{600} = 0.5) and late exponential phase (OD_{600} = 1.5) treated with miconazole. Data are n=1 wild-type culture.

9) Is there anything about the mechanism of action of different drugs, given that the hypothesis here is that efflux is regulated to increase drug tolerance, that would allow one to predict a priori which drugs should affect auxotrophs less than prototrophs? Does this match with the data from the drug libraries screened in this research?

Azoles and statins target the ergosterol biosynthetic pathway, inhibiting the production of ergosterol by targeting Erg11p and Hmg1/2p respectively. However, both in the microbiome and SeMeCo screen, we observe that the effects are target independent; i.e. a broad range of drugs are less effective in the auxotrophs. So the reviewer is correct, if one could predict if and how a drug is affected by the general efflux transporters, the very same predictor might indeed indicate its efficacy in auxotrophs. However, predicting the association of drugs with specific efflux pumps is a complex protein structure problem in a different field of study, and hence outside of scope of the manuscript; but as the idea of the reviewer might stimulate further studies, we have added it to the discussion.

10) Would it follow from this work that prototrophs are fitter in the presence of other prototrophs under drug-treatment conditions (due to prototrophs "sucking up" more drug than auxotrophs, leading to an overall lower average dose per cell)? Is this testable or interesting?

This is an interesting thought of the reviewer, however In a typical setting, that seems unlikely, as the inner cell volume is much smaller, by orders of magnitude, as the volume outside cells in a community. Hence as long as the cells just take up the drug, and do not metabolize to non-toxic compounds (i.e. as long as there is no flux that removes the drug from the system) they would not be able to detoxify their surroundings for significant amounts. It adds that the drug concentration differences between metabolite consumers and producers are within one order of magnitude (Figure 4c). In a simple DDA, the cell density did not markedly affect resistance or tolerance, as shown by no difference in the size of and growth within the zone of inhibition.

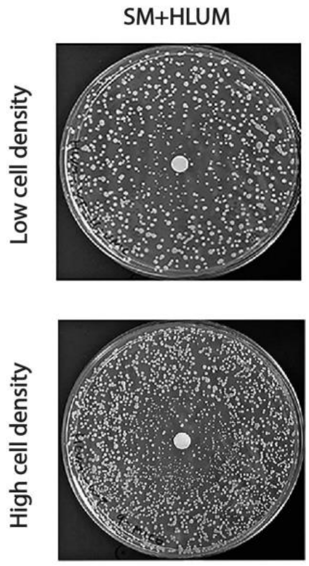

Cell plating density does not affect the drug tolerance response. DDA of cells plated at low and high densities on SM+HLUM agar and incubated for 72 h.

11) Should I view drug tolerance in microbial communities containing auxotrophs as an emergent property or as a direct function of the number of auxotrophs and hence more drug resistant species in the community? How does this translate to natural microbial communities, and in particular, to resilience of community composition? Would populations of purely cooperative auxotrophs be the most drug resistant? Or is it again a function of the proportion of the most drug resistant auxotroph in the community?

This is a nice point. Our data indicates that the increased drug tolerance is an emergent property that comes as a consequence of metabolite exchange interactions, where the degree of metabolite exchange is more complex than can be determined just by the number of auxotrophs present. Our metabolome data, FBA model (Figure 2) and proteome data (Figure 3) show that each of the different metabolites that we use (H, L, U, M), to model auxotroph-prototroph interactions, has a different impact on a broad range of metabolites and proteomes, and each of the metabolite titrations had a different quantitative effect on drug levels and resistance (Figure

4). Thus, the increase of tolerance is a function of the metabolite exchange activity between cells, which is stimulated by the presence of auxotrophs according to their degree of interactions with other cells. We have added this paragraph to the discussion.

Minor points

12) What is shown in Figure 1b? From reading the Figure caption, I think that it is showing the total number (out of 15 auxotrophic gut species) that had a leu auxotrophy, trp auxotrophy, etc. But in the text (Line 155) it seems to suggest that I can get from Figure 1b information about the number of auxotrophies per species. This is how the data is plotted in S1B though, so perhaps that is what you mean.

We apologize for the mistake on the labeling, that resulted from moving panels from the main figure to supplementary in order to make the figures easier to read. The reviewer is correct, Fig. 1b shows the auxotrophy counts per amino acids, while Supplementary Fig. S1b shows the auxotrophy count per species. This has been corrected in the manuscript.

13) Please clarify what is being plotted in Figure 1c. Is this the 40 gut microbiome species from Reference 39 (and indeed, is this data from Reference 39 reused/replotted for this study?)

Growth data as AUC values used to construct this figure are derived from the Maier et al., 2018 [1] resource dataset and correspond to the gut microbiome species. Maier et al. did not distinguish between autotrophic and prototrophic species, and we achieved this by integrating the Machado et al., 2020 [3] auxotrophy predictors (please see detailed explanation above in response to similar comment raised by Reviewer #1: comment #1).

14) How was the drug concentration chosen (Line 190)?

The drug concentration used for screening was chosen to be at 10 µM for every drug. The reason for this concentration is that this is the typical starting concentration used in many pharmacological screens [15]. We have added this information to the manuscript.

15) It seems strange to me to put the inset (e(i)) with the SeMeCos data since (i) is from the gut microbiome mostly bacterial strains and this is the yeast data.

We apologize that this figure was confusing. We have changed the figure and now show the bacterial and yeast data in separate panels. The original intention was to show that azoles were found to induce higher AUC values and a higher auxotrophic composition in the bacterial and yeast drug screens respectively, lending weight to the idea that both are correlated, at least with respect to this particular drug class.

16) Figure 1f: Is the heat map reversed? Red means a higher prototroph to auxotroph ratio, therefore fewer auxotrophs? Which seems the opposite of what the Figure is trying to show? I could just be confused. OR should I be comparing color changes across the number of

auxotrophies (i.e. less red for orange and purple populations). Also should the description explicitly state that P:A ratio stands for prototroph:auxotroph ratio?

We thank the reviewer for bringing this to our attention. Red indicated an increase in the specific population which would therefore have a higher auxotroph to prototroph (A:P) ratio. We have now changed the figure, agree this can be confusing, and make it explicit what we mean by this both in figure and text.

17) • Line 150: "host-associated" • Line 268 "shows" • Line 398-390: "More drug tolerant" • Line 393 and Lines 400-402: I think it would be helpful to mention the specific metabolites as part of this discussion. • Line 427: cause vs causes • Line 436: foster and improve • In figure 2C it would be easier to compare the figures if the y-axis was the same. • Line 801: delete "containing" • Line 819: "prototrophs" • Line 823-824: "...HLUM as opposed to…"

We thank the reviewer for identifying these small issues and have addressed them accordingly within the text and figures.

References:

1. Maier, L. et al. Extensive impact of non-antibiotic drugs on human gut bacteria. Nature 555, 623–628 (2018).
2. Thompson, L. R. et al. A communal catalogue reveals Earth's multiscale microbial diversity. Nature 551, 457–463 (2017).
3. Machado, D. et al. Polarization of microbial communities between competitive and cooperative metabolism. (2020).
4. Campbell, K. et al. Self-establishing communities enable cooperative metabolite exchange in a eukaryote. Elife 4, e09943 (2015).
5. Berman, J. & Krysan, D. J. Drug resistance and tolerance in fungi. Nat. Rev. Microbiol. 18, (2020).
6. Vowinckel, J., Hartl, J., Butler, R. & Ralser, M. MitoLoc: A method for the simultaneous quantification mitochondrial network morphology and membrane potential in single cells. Mitochondrion (2015) doi:10.1016/j.mito.2015.07.001.

7. Alam, M. T. et al. The metabolic background is a global player in Saccharomyces gene

expression epistasis. Nat Microbiol 1, 15030 (2016).

8. Campbell, K., Vowinckel, J. & Ralser, M. Cell-to-cell heterogeneity emerges as consequence of metabolic cooperation in a synthetic yeast community. Biotechnol. J. (2016).

9. Olin-Sandoval, V. et al. Lysine harvesting is an antioxidant strategy and triggers underground polyamine metabolism. Nature 572, (2019).

10. M27Ed4: Broth dilution antifungal susceptibility, Yeasts. https://clsi.org/standards/products/microbiology/documents/ m27/.

11. M44: Antifungal disk diffusion susceptibility, Yeasts. https://clsi.org/standards/products/microbiology/documents/m44/.

12. Revell, L. J. & Revell, M. L. J. Package 'phytools'. Website: https://cran. r-proje ct. org/web/packa ges/phytools (2014).

13. Messner, C. B. et al. Ultra-fast proteomics with Scanning SWATH. Nat. Biotechnol. (2021) doi:10.1038/s41587-021-00860-4.

14. Ponomarova, O. et al. Yeast Creates a Niche for Symbiotic Lactic Acid Bacteria through Nitrogen Overflow. Cell Syst 5, 345–357.e6 (2017).

15. Hughes, J. P., Rees, S., Kalindjian, S. B. & Philpott, K. L. Principles of early drug discovery. Br. J. Pharmacol. 162, 1239 (2011).

Decision Letter, first revision:

Dear Markus,

Thank you for your patience while your manuscript "Microbial communities form rich extracellular metabolomes that foster cooperation and promote drug tolerance" was under peer-review at Nature

Microbiology. It has now been seen by 3 referees, whose expertise and comments you will find at the end of this email. Although they find your work of some potential interest, they have raised a number of concerns that will need to be addressed before we can consider publication of the work in Nature Microbiology.

In particular, you will see that referee #1 still raises some concerns. The reviewer asks if efflux pumps are upreuglated in auxotrophs. The referee also asks to perform further flux balance analyses with a single auxotroph to rule out alternative explanations for auxotroph metabolite excretion.

Should further experimental data allow you to address these criticisms, we would be happy to look at a revised manuscript. Unless these concerns are clarified, we won't be able to proceed with publication.

We are committed to providing a fair and constructive peer-review process. Please do not hesitate to contact us if there are specific requests from the reviewers that you believe are technically impossible or unlikely to yield a meaningful outcome.

We strongly support public availability of data. Please place the data used in your paper into a public data repository, if one exists, or alternatively, present the data as Source Data or Supplementary Information. If data can only be shared on request, please explain why in your Data Availability Statement, and also in the correspondence with your editor. For some data types, deposition in a public repository is mandatory - more information on our data deposition policies and available repositories can be found at https://www.nature.com/nature-research/editorial-policies/reporting-standards#availability-of-data.

Please include a data availability statement as a separate section after Methods but before references, under the heading "Data Availability”. This section should inform readers about the availability of the data used to support the conclusions of your study. This information includes accession codes to public repositories (data banks for protein, DNA or RNA sequences, microarray, proteomics data etc…), references to source data published alongside the paper, unique identifiers such as URLs to data repository entries, or data set DOIs, and any other statement about data availability. At a minimum, you should include the following statement: “The data that support the findings of this study are available from the corresponding author upon request”, mentioning any restrictions on availability. If DOIs are provided, we also strongly encourage including these in the Reference list (authors, title, publisher (repository name), identifier, year). For more guidance on how to write this section please see: http://www.nature.com/authors/policies/data/data-availability-statements-data-citations.pdf

If revising your manuscript:

* Include a “Response to referees” document detailing, point-by-point, how you addressed each referee comment. If no action was taken to address a point, you must provide a compelling argument. This response will be sent back to the referees along with the revised manuscript.

* If you have not done so already we suggest that you begin to revise your manuscript so that it conforms to our Article format instructions at http://www.nature.com/nmicrobiol/info/final-submission. Refer also to any guidelines provided in this letter.

* Include a revised version of any required reporting checklist. It will be available to referees (and, potentially, statisticians) to aid in their evaluation if the manuscript goes back for peer review. A revised checklist is essential for re-review of the paper.

When submitting the revised version of your manuscript, please pay close attention to our href="https://www.nature.com/nature-research/editorial-policies/image-integrity">Digital Image

Integrity Guidelines. and to the following points below:

-- that unprocessed scans are clearly labelled and match the gels and western blots presented in figures.
-- that control panels for gels and western blots are appropriately described as loading on sample processing controls
-- all images in the paper are checked for duplication of panels and for splicing of gel lanes.

Finally, please ensure that you retain unprocessed data and metadata files after publication, ideally archiving data in perpetuity, as these may be requested during the peer review and production process or after publication if any issues arise.

Please use the link below to submit a revised paper:

{redacted}

Note: This url links to your confidential homepage and associated information about manuscripts you may have submitted or be reviewing for us. If you wish to forward this e-mail to co-authors, please delete this link to your homepage first.

Nature Microbiology is committed to improving transparency in authorship. As part of our efforts in this direction, we are now requesting that all authors identified as 'corresponding author' on published papers create and link their Open Researcher and Contributor Identifier (ORCID) with their account on the Manuscript Tracking System (MTS), prior to acceptance. This applies to primary research papers only. ORCID helps the scientific community achieve unambiguous attribution of all scholarly contributions. You can create and link your ORCID from the home page of the MTS by clicking on 'Modify my Springer Nature account'. For more information please visit please visit www.springernature.com/orcid.

If you wish to submit a suitably revised manuscript we would hope to receive it within 6 months. If you cannot send it within this time, please let us know. We will be happy to consider your revision, even if a similar study has been accepted for publication at Nature Microbiology or published elsewhere (up to a maximum of 6 months).

In the meantime we hope that you find our referees' comments helpful.

Yours sincerely,

{redacted}

****
Reviewer Expertise:

Referee #1: metabolic interactions/microbial communities
Referee #2: drug tolerance
Referee #3: microbial ecology/communities

Reviewer Comments:

Reviewer #1 (Remarks to the Author):

This manuscript contains some really interesting data, and I appreciate the extensive additional work that the authors have done to clarify their work. I still have a couple of questions.

I believe that the mechanisms that the authors are proposing are that:
1) Auxotrophic cells express efflux pumps at higher levels during growth and this high level of efflux protects them from antibiotics.
2) Auxotrophs secrete high levels of amino acids, and these extracellular amino acids then also protect protrophs in the community from antifungals.

My first question is whether efflux pumps are indeed upregulated in auxotrophs? I believe the authors have this data from the proteome work and reporting the activity of genes involved in efflux could significantly strengthen the argument.

In figure 2Aiv I believe that most of the export from the auxotroph is the result of optimizing the community growth rather than a single genotype. I would encourage the authors to also do FBA with a single auxotroph in SM amended with the amino acid it requires. I suspect that this would not show excretion of any amino acids by the auxotroph. If this is true then it might argue that amino acid uptake by an auxotroph is not sufficient to cause the observed excretion of metabolites from the auxotroph. This then does not answer the question of why amino acid export is observed in SeMeCo experiments. My intuition is that the amino acid excretion by auxotrophs is the result of maladaptive gene expression in cells that have recently lost a metabolic enzyme. Indeed, amino acid excretion is commonly seen in auxotrophic bacteria for this reason. If this is true then running a Minimization of Metabolic Adjustment (https://doi.org/10.1073/pnas.232349399) on an auxotroph might provide even better fit between the metabolic model and the observed expression.

I think that the definitions of resistance and tolerance that have now been included are quite helpful. Resilience is also repeatedly used and a definition could be useful.

Page 7 line 8 – group "of" drugs.

Page 12 line 25 – Aren't the pathways that had lower flux in prototrophs (relative to auxotrophs), by definition the exact same as the pathways that had higher flux in auxotrophs? Perhaps I misunderstand what is being compared?

Page 16, line 10 – My understanding of the Black Queen Hypothesis is that cells leak costly benefits. Producers and consumers can be stabilized because a Snowdrift game arises, such that when producers are rare they have an advantage over consumers. In this scenario it is not that excretions are necessarily costless but rather that cells are incapable of privatizing their resources that drives the exchange. I offer this only as a perspective however.

Reviewer #2 (Remarks to the Author):

No further comments.

Reviewer #3 (Remarks to the Author):

In this article, the authors use measurements of the SeMeCo community to good advantage in combination with metabolic modeling to explore why auxotrophs confer antimicrobial resistance in microbial communities. This is complemented with analysis of existing microbiome data (for auxotroph frequency) as well as drug-resistance for members of the human microbiome community (growth in the presence of drugs). There are some parallel findings between the analysis of the previously published data and what the authors find in the SeMeCo yeast community for azole drugs.
As I look back on my prior review, most of my comments involved confusion regarding interpretation

and some questions driven by my own curiosity with answers that fall outside of the scope of this article. My comments regarding interpretation have been addressed with clarifications within the text. My curiosities have been brought into the discussion. My experimental comments which required some addition work have been answered with new data (Supplemental Figure 4). Therefore I believe that the authors have satisfactorily addressed my previous review.

Author Rebuttal, first revision:

Reviewer Comments:

Reviewer #1 (Remarks to the Author):

This manuscript contains some really interesting data, and I appreciate the extensive additional work that the authors have done to clarify their work. I still have a couple of questions.

I believe that the mechanisms that the authors are proposing are that:
1) Auxotrophic cells express efflux pumps at higher levels during growth and this high level of efflux protects them from antibiotics.
2) Auxotrophs secrete high levels of amino acids, and these extracellular amino acids then also protect prototrophs in the community from antifungals.

We thank the reviewer for their positive comments.

My first question is whether efflux pumps are indeed upregulated in auxotrophs? I believe the authors have this data from the proteome work and reporting the activity of genes involved in efflux could significantly strengthen the argument.

We thank the Reviewer for suggesting to study the expression levels of membrane exporters. This is a reasonable question to ask, however, we would like to point out that the activity of membrane exporters in eukaryotes is only partially regulated hierarchically, i.e. via their expression levels [1,2]. Furthermore, standard proteomic workflows are not optimized for the capture of membrane-bound transporters, as they are of low solubility in the aqueous phase. To address the reviewer's question, we have hence made use of a transcriptome dataset that we have recorded for auxotrophs in a study we have previously published previously [3]. We find that two out of the three most relevant plasma membrane ATP-binding cassette (ABC) transporters with antifungal activity [4–6], are expressed at higher levels across many auxotrophs, in comparison to prototrophs. Indeed, some of the strongest responses we observe are in the pleiotropic drug resistance 5 (PDR5), an ABC transporter that is known to also export antifungal drugs [4]. We thank the reviewer for the suggestion, these results further strengthen our study, and they have been included as a Supplementary Fig. S9.

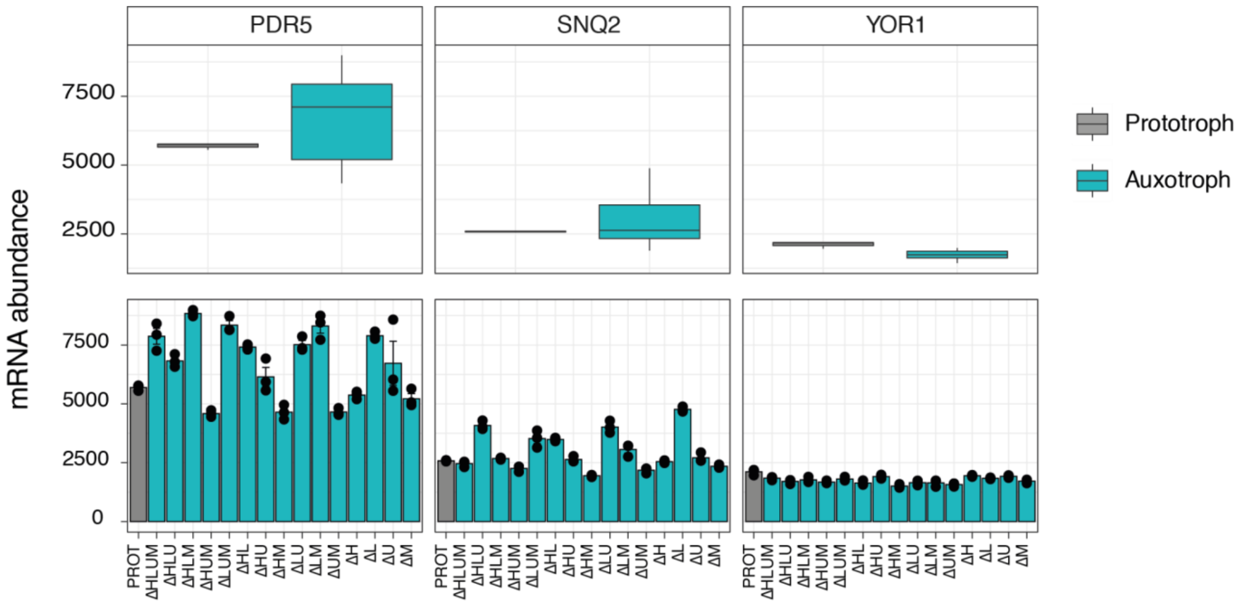

Expression of multidrug plasma membrane ABC transporters in H,L, U and M auxotrophs and prototrophs. Supplementary Fig. S9: mRNA expression profiles for the ABC transporters *PDR5, SNQ2,* and *YOR1* relevant for antifungal activity [4–6] were obtained from RNASeq expression data of 16 strains (1 prototroph and 15 auxotrophs in all possible combinations of HLUM) in triplicate of exponentially grown cultures, at similar cell density (optical density at 600 nm (OD_{600}) of 0.8), followed by mRNA sequencing [3]. Analysis was performed either by **(top)** grouping prototrophs and auxotrophs normalised mRNA expression levels (box plots represent median (50% quantile (middle line) and lower and upper quantiles (lower (25% quantile) and upper (75% quantile) or **(bottom)** by each background strain (bar plots represent mean±SEM of 3 independent cultures per strain, dots refer to individual cultures normalised mRNA abundance). The raw data (gene-wise read counts for gene expression estimation) was then processed using '*DEseq2*' [7] in R for normalization.

In figure 2Aiv I believe that most of the export from the auxotroph is the result of optimizing the community growth rather than a single genotype. I would encourage the authors to also do FBA with a single auxotroph in SM amended with the amino acid it requires. I suspect that this would not show excretion of any amino acids by the auxotroph. If this is true then it might argue that amino acid uptake by an auxotroph is not sufficient to cause the observed excretion of metabolites from the auxotroph. This then does not answer the question of why amino acid export is observed in SeMeCo experiments. My intuition is that the amino acid excretion by auxotrophs is the result of maladaptive gene expression in cells that have recently lost a metabolic enzyme. Indeed, amino acid excretion is commonly seen in auxotrophic bacteria for this reason. If this is true then running a Minimization of Metabolic Adjustment (https://doi.org/10.1073/pnas.232349399) on an auxotroph might provide even better fit between the metabolic model and the observed expression.

The reviewer makes an important point. First, we would like to take the opportunity to reiterate however that the we use the FBA model in a hypothesis generating way: that means, the the

focus of the modeling our study was to make use of the model for pointing us in the right direction (that did eventually allow us to formulate a mechanism that connects metabolic interactions and drug resilience). Hence, being critical like the reviewer about complex metabolic models (they never capture all biological properties), we extensively rely on a combination of multiple experimental techniques (proteomics, microscopy, growth assays, FACS, disk diffusion assays, as well as re-analysis of existing datasets) to test these hypotheses. The FBA analysis is part of that overall strategy, but it is not standing alone for us to come to our conclusions.

Specifically, the Reviewer points to a limitation of the FBA analysis first noted (and solved) by Segrè et al [8] in situaionts when a cell (outside of a community) is rendered auxotrophic by the knock-out of a gene. In that case, the FBA predicts that the microbial cell would reconfigure its metabolism to optimize and accelerate its growth rate. However, most single auxotrophs are growing at the same rate or slower than the wild-type, as we and others have shown previously
[9]. The MOMA approach as presented by Segre *et al* [8] overcomes this specific problem, by making the assumption that metabolic fluxes undergo a minimal redistribution with respect to the flux configuration of the wild type, when a single gene is deleted.

As suggested by the reviewer, we have now run simulations (FBA and MOMA approaches), in which a single metabolite is provided to the yeast cell. In agreement with the work of Segre et al with knockouts [8], the MOMA provided a better agreement with the experimental observations in this scenario. The FBA predicted a faster growth rate of the auxotrophs in this scenario (which we do not observe), while the MOMA predicted an increase in metabolite excretion. Although we have not addressed wild-type yeast cell supplementations with a single metabolite in our study, these results are hence supportive to our conclusion, as they show the metabolic re-configuration that follows a switch from self-synthesis to uptake results in increased efflux also in a MOMA analysi, also in the single gene knock-out. We have included these results as a Supplementary Fig. 3d. We have also slightly changed the order of the manuscript, and now present the results of the growth rate experiment next to the metabolic models.

As discussed previously, the MOMA approach was however difficult to model the metabolite exchange interactions between auxotrophs and prototrophs inside a SeMeCo. The MOMA method requires the inclusion of a metabolite supplementation constraint to the modeling which is not present in SeMeCos.

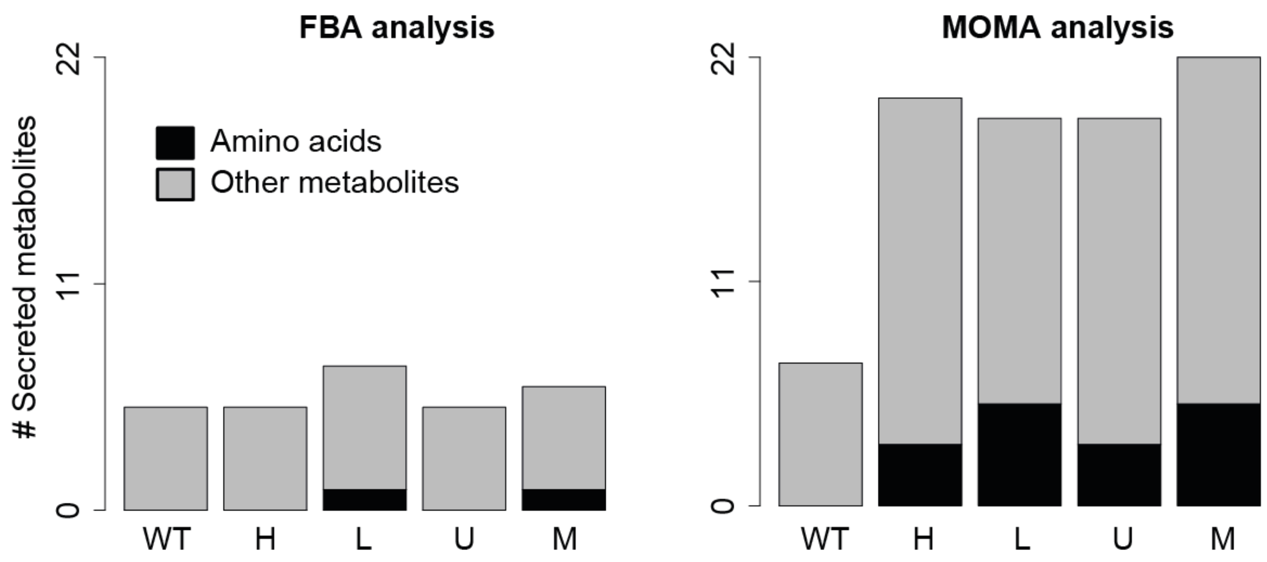

Supplementary Fig. S3d. Number of secreted metabolites in prototrophic (wild-type) and auxotrophic models in minimal media supplemented with required metabolites using FBA and MOMA simulation approaches, respectively. The MOMA predicts an increase in metabolite excretion in single-metabolite supplemented wild-type yeast, compared to an FBA analysis.

I think that the definitions of resistance and tolerance that have now been included are quite helpful. Resilience is also repeatedly used and a definition could be useful.

We agree with the Reviewer. Resilience is used in our paper when referring to cells or communities that demonstrate improved growth under treatment due to both resistance and tolerance mechanisms. We have now clearly defined this in the text upon first use.

Page 7 line 8 – group "of" drugs.

This has been corrected.

Page 12 line 25 – Aren't the pathways that had lower flux in prototrophs (relative to auxotrophs), by definition the exact same as the pathways that had higher flux in auxotrophs? Perhaps I misunderstand what is being compared?

Response: We apologise if this paragraph is still confusing. It might be intuitive that there is a reciprocal response, but it is reassuring that the data shows this. We have revised the paragraph for clarity.

Page 16, line 10 – My understanding of the Black Queen Hypothesis is that cells leak costly benefits. Producers and consumers can be stabilized because a Snowdrift game arises, such that when producers are rare they have an advantage over consumers. In this scenario it is not

that excretions are necessarily costless but rather that cells are incapable of privatizing their resources that drives the exchange. I offer this only as a perspective however.

We have taken the suggestion of the Reviewer into account, and agree that the Black Queen Hypothesis can be interpreted this way. We have hence revised our discussion and added the constraint that cells incapable of privatizing their resources can drive metabolite exchange.

Reviewer #2 (Remarks to the Author):

No further comments.

Reviewer #3 (Remarks to the Author):

In this article, the authors use measurements of the SeMeCo community to good advantage in combination with metabolic modeling to explore why auxotrophs confer antimicrobial resistance in microbial communities. This is complemented with analysis of existing microbiome data (for auxotroph frequency) as well as drug-resistance for members of the human microbiome community (growth in the presence of drugs). There are some parallel findings between the analysis of the previously published data and what the authors find in the SeMeCo yeast community for azole drugs.
As I look back on my prior review, most of my comments involved confusion regarding interpretation and some questions driven by my own curiosity with answers that fall outside of the scope of this article. My comments regarding interpretation have been addressed with clarifications within the text. My curiosities have been brought into the discussion. My experimental comments which required some additional work have been answered with new data (Supplemental Figure 4). Therefore I believe that the authors have satisfactorily addressed my previous review.

We thank the Reviewer for highlighting the novel aspects of our study and for supporting our manuscript for publication. We are glad that the interpretation of the data is now clear and hope that this somewhat satisfies their curiosity, which we have tried to incorporate as much as possible into the discussion.

References

1. Czuba, L. C., Hillgren, K. M. & Swaan, P. W. Post-translational modifications of transporters. *Pharmacology & Therapeutics* vol. 192 88–99 (2018).

2. Egner, R., Mahé, Y., Pandjaitan, R. & Kuchler, K. Endocytosis and vacuolar degradation of the plasma membrane-localized Pdr5 ATP-binding cassette multidrug transporter in Saccharomyces cerevisiae. *Mol. Cell. Biol.* **15**, 5879–5887 (1995).
3. Alam, M. T. *et al.* The metabolic background is a global player in Saccharomyces gene expression epistasis. *Nat Microbiol* **1**, 15030 (2016).
4. Suzuki, Y. *et al.* Knocking out multigene redundancies via cycles of sexual assortment and fluorescence selection. *Nat. Methods* **8**, 159–164 (2011).
5. Kolaczkowska, A., Kolaczkowski, M., Goffeau, A. & Moye-Rowley, W. S. Compensatory activation of the multidrug transporters Pdr5p, Snq2p, and Yor1p by Pdr1p in Saccharomyces cerevisiae. *FEBS Lett.* **582**, 977–983 (2008).
6. Paumi, C. M., Chuk, M., Snider, J., Stagljar, I. & Michaelis, S. ABC Transporters in Saccharomyces cerevisiae and Their Interactors: New Technology Advances the Biology of the ABCC (MRP) Subfamily. *Microbiol. Mol. Biol. Rev.* **73**, 577 (2009).
7. Love, M. I., Huber, W. & Anders, S. Moderated estimation of fold change and dispersion for RNA-seq data with DESeq2. *Genome Biol.* **15**, 550 (2014).
8. Segrè, D., Vitkup, D. & Church, G. M. Analysis of optimality in natural and perturbed metabolic networks. *Proc. Natl. Acad. Sci. U. S. A.* **99**, 15112–15117 (2002).
9. Mülleder, M. *et al.* A prototrophic deletion mutant collection for yeast metabolomics and systems biology. *Nat. Biotechnol.* **30**, 1176–1178 (2012).

Decision Letter, second revision:

Dear Markus,

Thank you for submitting your revised manuscript "Microbial communities form rich extracellular metabolomes that foster metabolic interactions and promote drug tolerance" (NMICROBIOL-21030711B). It has now been seen by referee #1 and their comments are below. The reviewers find that the paper has improved in revision, and therefore we'll be happy in principle to publish it in

Nature Microbiology, pending minor revisions to satisfy the referees' final requests and to comply with our editorial and formatting guidelines.

We are now performing detailed checks on your paper and will send you a checklist detailing our editorial and formatting requirements in about a week. Please do not upload the final materials and make any revisions until you receive this additional information from us.

Thank you again for your interest in Nature Microbiology Please do not hesitate to contact me if you have any questions.

Sincerely,

{redacted}

Reviewer #1 (Remarks to the Author):

I greatly appreciate the authors' attention to my suggestions. As noted in emails to both the editors and the authors, upon reflection I realized that my comments were likely to be less helpful than intended.

I appreciate the authors' inclusion of the transcriptomic data on top of the elegant work that they already did with fluorescent proteins and dyes. I also appreciate the added FBA analyses. The authors have previously shown that amino acid uptake is sufficient to alter excretion profiles, and I completely agree with the authors that the FBA results are not foundational to their arguments in the current paper. I do think the additional analyses provide some additional intuition as to why the model is bolstering laboratory observations, however.

This is a highly interesting paper that I think is likely to drive advancement in the field.

Decision Letter, Final checks:

Dear Markus,

Thank you for your patience as we've prepared the guidelines for final submission of your Nature Microbiology manuscript, "Microbial communities form rich extracellular metabolomes that foster metabolic interactions and promote drug tolerance" (NMICROBIOL-21030711B). Please carefully follow the step-by-step instructions provided in the attached file, and add a response in each row of the table to indicate the changes that you have made. Please also check and comment on any additional marked-up edits we have proposed within the text. Ensuring that each point is addressed will help to ensure that your revised manuscript can be swiftly handed over to our production team.

We would like to start working on your revised paper, with all of the requested files and forms, as soon as possible (preferably within two weeks). Please get in contact with us if you anticipate delays.

When you upload your final materials, please include a point-by-point response to any remaining reviewer comments.

If you have not done so already, please alert us to any related manuscripts from your group that are under consideration or in press at other journals, or are being written up for submission to other journals (see: https://www.nature.com/nature-research/editorial-policies/plagiarism#policy-on-duplicate-publication for details).

In recognition of the time and expertise our reviewers provide to Nature Microbiology's editorial process, we would like to formally acknowledge their contribution to the external peer review of your manuscript entitled "Microbial communities form rich extracellular metabolomes that foster metabolic interactions and promote drug tolerance". For those reviewers who give their assent, we will be publishing their names alongside the published article.

Nature Microbiology offers a Transparent Peer Review option for new original research manuscripts submitted after December 1st, 2019. As part of this initiative, we encourage our authors to support increased transparency into the peer review process by agreeing to have the reviewer comments, author rebuttal letters, and editorial decision letters published as a Supplementary item. When you submit your final files please clearly state in your cover letter whether or not you would like to participate in this initiative. Please note that failure to state your preference will result in delays in accepting your manuscript for publication.

Cover suggestions

As you prepare your final files we encourage you to consider whether you have any images or illustrations that may be appropriate for use on the cover of Nature Microbiology.

Covers should be both aesthetically appealing and scientifically relevant, and should be supplied at the best quality available. Due to the prominence of these images, we do not generally select images featuring faces, children, text, graphs, schematic drawings, or collages on our covers.

We accept TIFF, JPEG, PNG or PSD file formats (a layered PSD file would be ideal), and the image should be at least 300ppi resolution (preferably 600-1200 ppi), in CMYK colour mode.

If your image is selected, we may also use it on the journal website as a banner image, and may need to make artistic alterations to fit our journal style.

Please submit your suggestions, clearly labeled, along with your final files. We'll be in touch if more information is needed.

Nature Microbiology has now transitioned to a unified Rights Collection system which will allow our Author Services team to quickly and easily collect the rights and permissions required to publish your work. Approximately 10 days after your paper is formally accepted, you will receive an email in providing you with a link to complete the grant of rights. If your paper is eligible for Open Access, our Author Services team will also be in touch regarding any additional information that may be required to arrange payment for your article.

Please note that you will not receive your proofs until the publishing agreement has been received through our system.

Please note that <i>Nature Microbiology</i> is a Transformative Journal (TJ). Authors may publish their research with us through the traditional subscription access route or make their paper immediately open access through payment of an article-processing charge (APC). Authors will not be

required to make a final decision about access to their article until it has been accepted. Find out more about Transformative Journals

<B>Authors may need to take specific actions to achieve compliance with funder and institutional open access mandates. For submissions from January 2021, if your research is supported by a funder that requires immediate open access (e.g. according to Plan S principles) then you should select the gold OA route, and we will direct you to the compliant route where possible. For authors selecting the subscription publication route our standard licensing terms will need to be accepted, including our self-archiving policies. Those standard licensing terms will supersede any other terms that the author or any third party may assert apply to any version of the manuscript.

For information regarding our different publishing models please see our Transformative Journals page. If you have any questions about costs, Open Access requirements, or our legal forms, please contact ASJournals@springernature.com.

Please use the following link for uploading these materials:
{redacted}

If you have any further questions, please feel free to contact me.

{redacted}

Reviewer #1:
Remarks to the Author:
I greatly appreciate the authors’ attention to my suggestions. As noted in emails to both the editors and the authors, upon reflection I realized that my comments were likely to be less helpful than intended.

I appreciate the authors’ inclusion of the transcriptomic data on top of the elegant work that they already did with fluorescent proteins and dyes. I also appreciate the added FBA analyses. The authors have previously shown that amino acid uptake is sufficient to alter excretion profiles, and I completely agree with the authors that the FBA results are not foundational to their arguments in the current paper. I do think the additional analyses provide some additional intuition as to why the model is bolstering laboratory observations, however.

This is a highly interesting paper that I think is likely to drive advancement in the field.

Final Decision Letter:

Dear Markus,

I am pleased to accept your Article "Microbial communities form rich extracellular metabolomes that foster metabolic interactions and promote drug tolerance" for publication in Nature Microbiology. Thank you for having chosen to submit your work to us and many congratulations.

Over the next few weeks, your paper will be copyedited to ensure that it conforms to Nature Microbiology style. We look particularly carefully at the titles of all papers to ensure that they are relatively brief and understandable.

Once your paper is typeset, you will receive an email with a link to choose the appropriate publishing options for your paper and our Author Services team will be in touch regarding any additional information that may be required. Once your paper has been scheduled for online publication, the Nature press office will be in touch to confirm the details.

After the grant of rights is completed, you will receive a link to your electronic proof via email with a request to make any corrections within 48 hours. If, when you receive your proof, you cannot meet this deadline, please inform us at rjsproduction@springernature.com immediately. You will not receive your proofs until the publishing agreement has been received through our system

Due to the importance of these deadlines, we ask you please us know now whether you will be difficult to contact over the next month. If this is the case, we ask you provide us with the contact information (email, phone and fax) of someone who will be able to check the proofs on your behalf, and who will be available to address any last-minute problems.

Acceptance of your manuscript is conditional on all authors' agreement with our publication policies (see https://www.nature.com/nmicrobiol/editorial-policies). In particular your manuscript must not be published elsewhere and there must be no announcement of the work to any media outlet until the publication date (the day on which it is uploaded onto our website).

Please note that <i>Nature Microbiology</i> is a Transformative Journal (TJ). Authors may publish their research with us through the traditional subscription access route or make their paper immediately open access through payment of an article-processing charge (APC). Authors will not be required to make a final decision about access to their article until it has been accepted. Find out more about Transformative Journals

<B>Authors may need to take specific actions to achieve compliance with funder and institutional open access mandates. For submissions from January 2021, if your research is supported by a funder that requires immediate open access (e.g. according to Plan S principles) then you should select the gold OA route, and we will direct you to the compliant route where possible. For authors selecting the subscription publication route our standard licensing terms will need to be accepted, including our self-archiving policies. Those standard licensing terms will supersede any other terms that the author or any third party may assert apply to any version of the manuscript.

If you have any questions about our publishing options, costs, Open Access requirements, or our legal

forms, please contact ASJournals@springernature.com

An online order form for reprints of your paper is available at https://www.nature.com/reprints/author-reprints.html. All co-authors, authors' institutions and authors' funding agencies can order reprints using the form appropriate to their geographical region.

We welcome the submission of potential cover material (including a short caption of around 40 words) related to your manuscript; suggestions should be sent to Nature Microbiology as electronic files (the image should be 300 dpi at 210 x 297 mm in either TIFF or JPEG format). Please note that such pictures should be selected more for their aesthetic appeal than for their scientific content, and that colour images work better than black and white or grayscale images. Please do not try to design a cover with the Nature Microbiology logo etc., and please do not submit composites of images related to your work. I am sure you will understand that we cannot make any promise as to whether any of your suggestions might be selected for the cover of the journal.

You can now use a single sign-on for all your accounts, view the status of all your manuscript submissions and reviews, access usage statistics for your published articles and download a record of your refereeing activity for the Nature journals.

To assist our authors in disseminating their research to the broader community, our SharedIt initiative provides you with a unique shareable link that will allow anyone (with or without a subscription) to read the published article. Recipients of the link with a subscription will also be able to download and print the PDF.

As soon as your article is published, you will receive an automated email with your shareable link.
